# Manifold learning for olfactory habituation to strongly fluctuating backgrounds

**DOI:** 10.1101/2025.05.26.656161

**Published:** 2025-05-30

**Authors:** François X. P. Bourassa, Paul François, Gautam Reddy, Massimo Vergassola

**Affiliations:** a Joseph Henry Laboratories of Physics, Princeton University, Princeton, NJ 08544, USA; b Department of Physics, McGill University, 3600 rue University, Montréal, QC, H3A 2T8, Canada; c Département de biochimie et médecine moléculaire, Université de Montréal, 5155 Chemin de la Rampe, Montréal, QC, H3T 1J4, Canada; d MILA Québec, 6666, rue Saint-Urbain, Montréal, QC, H2S 3H1, Canada; e Laboratoire de Physique de l’Ecole normale supérieure (ENS), Université PSL, CNRS, Sorbonne Université, Université de Paris, F-75005 Paris, France; f Department of Physics, University of California, San Diego, 9500 Gillman Drive, La Jolla, CA 92093, USA

**Keywords:** olfaction, fluctuating environments, habituation, manifold learning, theoretical neuroscience

## Abstract

Animals rely on their sense of smell to survive, but important olfactory cues are mixed with confounding background odors that fluctuate due to atmospheric turbulence. It is unclear how the olfactory system habituates to such stochastic backgrounds to detect behaviorally important odors. Here, we explicitly consider the high-dimensional nature of odor coding, the natural statistics of odor fluctuations and the architecture of the early olfactory pathway. We show that their combination favors a manifold learning mechanism for olfactory habituation over alternatives based on predictive filtering. Manifold learning is implemented in our model by a biologically plausible network of inhibitory interneurons in the early olfactory pathway. We demonstrate that plasticity rules based on IBCM or online PCA are effective at implementing this mechanism in turbulent conditions and outperform previous models relying on mean background subtraction. Interneurons with an IBCM plasticity rule acquire selectivity to independently varying odors. This manifold learning mechanism offers a path towards distinguishing plasticity rules in experiments and could be leveraged by other biological circuits facing fluctuating environments.

## Introduction

Most of us have experienced an odor fading to imperceptibility after prolonged exposure. Habituation is a basic building block of sensory cognition, allowing us to pay attention to weak but important cues relevant for survival [1, 2]. Across sensory modalities, numerous mechanisms for sensory adaptation and habituation filter out irrelevant information; these mechanisms must be considered in light of the statistical features of natural scenes [3-7]. Though olfactory habituation to regular stimuli is behaviorally well-characterized [8], less is known about its computational basis in neural circuits facing naturalistic environments.

**Fig. 1.**
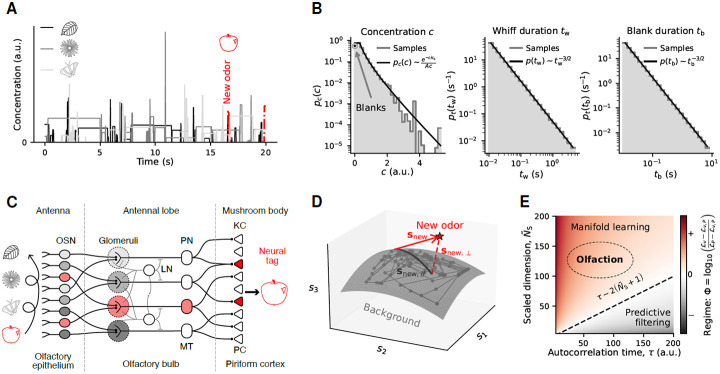
Olfactory systems face stochastic, turbulent odor mixture inputs, for which manifold learning may be an optimal habituation strategy. (**A**) Illustration of background and new odor concentrations time series, strongly fluctuating in a series of whiffs and blanks, according to the turbulent atmosphere statistics derived in [9]. (**B**) Stationary probability distributions of whiff concentrations and whiff and blank durations. (**C**) Structure of early layers in the olfactory network, annotated with fly (top) and mouse (bottom, when different from fly) anatomical regions and cell types. (**D**) Illustration of a hypothetical low-dimensional subspace spanned by background odors in the space of OSN activities $s_{i}$, with sample mixtures generated by log-normal odor concentrations. A new odor, $s_{\text{new}}$, generally has a component, $s_{\text{new},⊥}$, lying outside of the background manifold. (**E**) Log-ratio of the difference in loss functions for new odor recognition between manifold learning (${ℒ}_{P}$), predictive filtering (${ℒ}_{v}$), or the combination of both strategies (${ℒ}_{v,P}$). For a sensory system tasked to detect new odors within fast background fluctuations in a high-dimensional space, as it is the case with olfaction, manifold learning is the dominant strategy (loss ${ℒ}_{P}\approx {ℒ}_{v,P}$). The olfactory space dimensionality is rescaled by the relative variance of background and new odors: ${\tilde{N}}_{S}=N_{S}\sigma^{2}∕\sigma_{\text{new}}^{2}$.

The physics of odor transport poses a difficult habituation problem, challenging simple models such as mean background subtraction. Unlike in vision and audition, olfactory signals are transported by a physical medium that is turbulent at the spatial scales relevant for behavior. Wind velocities in such environments have complex spatial and temporal fluctuations, which segregate air into patches of odor and clean air (Fig. 1A). An olfactory sensory apparatus thus receives a highly intermittent sensory signal, where clumps of intense odor detections (‘whiffs’) are separated by seconds to minutes of relatively clean air (‘blanks’) (Fig. 1B) [9-11]. These strongly non-Gaussian statistics make it non-trivial for olfactory circuits to identify new odors mixed with a dominant, fluctuating background.

The neurobiology of early olfaction outlines the underlying circuit structure solving this habituation problem across animal species (Fig. 1C). In insects, odors are first detected by olfactory receptors (ORs) located on olfactory sensory neurons (OSNs) in the antennae. Each OSN often expresses one olfactory receptor type (among ~ 50 different receptor types in the fruit fly). Axons from OSNs expressing the same receptor project to distinct locations called glomeruli in the antennal lobe. Projection neurons (PNs) integrate signals from a few glomeruli and project to higher order processing centers, including the mushroom body (where associations are formed) and the lateral horn (which drives innate behaviors) [12, 13]. A strikingly similar architecture is present in mammals: glomeruli are located in the olfactory bulb where OSN input is processed and broadcast to diverse subcortical and cortical regions [14].

A characteristic feature of ORs is their broad tuning to many odor molecules, such that each OSN is activated by multiple odors [15]. Thus, OSN activity induced by a behaviorally relevant odor appearing in a naturalistic environment is masked by possibly many background odors [16]. Biophysical mechanisms for adaptation in a single OSN allow for adapting the dynamic range of spiking output to the statistics of receptor activity [17-20]. However, these single-neuron mechanisms do not, on their own, disentangle contributions from a new and relevant odor from those of irrelevant backgrounds, suggesting that background subtraction occurs at the neural population coding level, in a later stage of the olfactory pathway [21-24].

The antennal lobe (AL) in insects and the olfactory bulb (OB) in mammals are likely candidate regions for olfactory habituation. Since most olfactory receptors are promiscuous, the population activity of glomeruli in these regions represents an efficient combinatorial code for odors [25, 26]. The AL and OB further contain extensive local networks of inhibitory interneurons that mediate inter-glomerular crosstalk before signals are broadcast to downstream processing centers [27-30]. Consistent with this picture, plasticity mechanisms in *Drosophila* antennal lobe inhibitory interneurons (called lateral neurons (LNs)) have been linked to the formation of olfactory memories during habituation [6, 8, 22, 31-35]. Building on these results, Shen *et al.* proposed a neural model for olfactory habituation where LNs learn and subtract a time-averaged background signal by integrating glomerular activity over timescales of minutes [36]. While a mean filtering mechanism is plausible when backgrounds are stable, it cannot filter out the strong fluctuations of naturalistic olfactory scenes (Supp. Materials, sec. 3), hinting that other mechanisms are at play.

Here, we propose a conceptually distinct model for olfactory habituation to broadly activating backgrounds that fluctuate on physically relevant timescales. As in previous proposals, we assume that background subtraction is mediated by plasticity mechanisms in inhibitory interneurons within the AL and OB. Our model is motivated by the fact that the representation of a fluctuating odor traverses a one-dimensional manifold (*i.e.,* a curve) in a much higher-dimensional glomerular activity space [23]. Consequently, a mixture of backgrounds spans a manifold of dimensionality equal to the number of independently varying odors in the mixture. A network that learns this low-dimensional manifold can thereby subtract out background activity by projecting instantaneous activity to the low-dimensional background manifold, highlighting components that are orthogonal to it (Fig. 1D). Our proposed ‘manifold projection’ mechanism thus relies on the high-dimensional nature of olfactory coding, and is still applicable when odors fluctuate on timescales comparable to timescales of neural signal propagation. Hence, the characteristics of olfactory stimuli and circuits outline a distinct habituation mechanism at the level of neural population codes, which could also be leveraged by other biological circuits facing fluctuating backgrounds in high-dimensional input spaces.

The structure of the paper is as follows. We first use a minimal mathematical model to delineate the physical and sensory coding regimes where a manifold projection strategy outcompetes a predictive filtering mechanism for new signal recognition among strong background fluctuations. Next, we propose a biologically plausible model for manifold learning implemented by inhibitory interneurons in the early olfactory system. We consider two local plasticity rules (IBCM and BioPCA), which find the linear subspace spanned by the background odors. Interneurons equipped with either rule perform considerably better against fluctuating backgrounds than prior models and perform comparably against each other. A detailed mathematical analysis of the IBCM rule shows that interneurons acquire selectivity to independent odors in the mixture. Finally, we show that both plasticity rules are robust across a range of physiologically relevant physical and computational regimes.

## Results

### Regimes of predictive filtering and manifold learning.

We begin by delineating the physical and computational regimes in which a *manifold learning* strategy for habituation outperforms a *predictive filtering* strategy. We consider an olfactory system habituating for time T to a fluctuating background, b(t). The components of these vectors represent the glomerular activations corresponding to each OR type, and thus reflect the coordinates of an odor in an $N_{S}$-dimensional olfactory coding space. As in the rest of the paper, the backgrounds b(t) are mixtures of $N_{B}$ odor vectors,

(1) $$b(t)=\sum_{\gamma =1}^{N_{B}}c_{\gamma }(t){\hat{s}}_{\gamma },$$

where the concentration of the γth odor at time t is $c_{\gamma }(t)$. Here, we have assumed additive odor mixtures to keep our analysis tractable; our conceptual argument should also extend to non-additive odor mixture coding [17, 37-39] when combined with algorithms for curved manifolds (*Discussion*). The system subsequently responds at time T to a mixture of the fluctuating background b(T) and a new, behaviorally relevant target odor $s_{\text{new}}$ which the target aims to recognize; the total input is $b(T)+s_{\text{new}}$. An idealized circuit subtracts a vector $u_{T}$ from the target-background mixture, where

(2) $$u_{T}=\sum_{j=1}^{T-1}v_{j}b(T-j)+P(b(T)+s_{\text{new}})\;.$$

The first term represents a weighted average over the background’s history, corresponding to the predictive filtering strategy, where scalar coefficients $v_{j}$ are set by the second-order statistics of background fluctuations. The second term corresponds to a simplified manifold learning strategy, where the matrix P projects the current stimulus to the subspace spanned by the background. The parameters $v_{j}$ and P are learned during habituation such that the target odor $s_{\text{new}}$ is recovered (in the mean squared error sense) by subtracting $u_{T}$ from the current input.

We analytically optimized $v_{j}$ and P to delineate how the two strategies contribute to recovering the target odor $s_{\text{new}}$ from the mixture with b(T), which depends on background statistics (Supp. Materials, sec. 1). Fig. 1E illustrates the optimal reconstruction error for different autocorrelation timescales of background fluctuations (τ), and dimensionalities of the olfactory coding space ($N_{S}$, Fig. S1A-B). While the combined strategy is by construction always better than each individual strategy, manifold learning alone explains all the performance when fluctuations are fast and the dimensionality is large (small τ, large $N_{S}$).

The spectrum of turbulent fluctuations is dominated by brief whiffs and blanks that can reach down to ~10 ms (Fig. 1B); predictive filtering, since it acts as a change detector, would constantly respond to these whiffs. Taking the filtering time step to be the smallest olfactory delay functionally perceptible to mice and human (30-60 ms) [40, 41], we estimate that τ<100 for typical turbulent backgrounds (Fig. S1C). The number of OR types spans from a few tens to thousands across different animals. The olfactory space is high-dimensional compared to the typical number of independent odor sources that might prevail in a natural landscape. Thus, physics and neurobiology together indicate that olfaction lies within the regime where a manifold learning strategy is most effective.

### Models of manifold learning in the early olfactory circuit to improve new odor recognition.

**Fig. 2.**
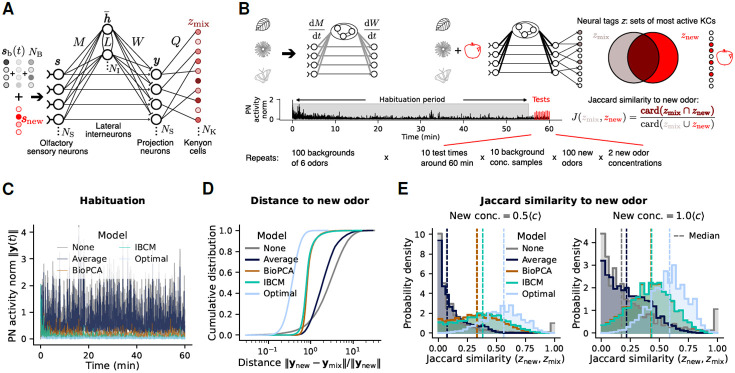
Recognition of new odors after habituation to a background with different learning models. (**A**) Mathematical description of the olfactory network. (**B**) Schematic of the numerical experiments performed to assess habituation performance. We generate a set of background odors randomly, then integrate the network’s synaptic plasticity equations for 60 minutes of habituation to a simulated background time series, where odor concentrations fluctuate according to the turbulent stochastic process illustrated in Fig. 1A-B (see Supp. Materials sec. 2 for simulation methods). We then compute the network’s response to a new odor $s_{\text{new}}$ mixed with the background. The recognition performance is quantified by the Jaccard similarity between the neural tag of the mixture, $z_{\text{mix}}$, and the neural tag (pre-habituation) of the new odor alone, $z_{\text{new}}$. This procedure is repeated for several test times, random backgrounds, samples of each background, random new odors, and different new odor concentrations, for each habituation model (none, average subtraction, BioPCA, IBCM). Parameter values are listed in the Methods. (**C**) Sample time series of the norm of PN activity, to illustrate the extent of habituation (decrease in PN activity when exposed to the background) in each model. “Optimal”: response with the optimal manifold learning matrix P (no predictive filtering) derived for Fig. 1E. (**D**) Cumulative distribution of the Euclidean distance between new odors $s_{\text{new}}$ and each model’s PN response to new odors mixed with the background, $y_{\text{mix}}$, after habituation, across all background, odors, and new odor concentrations tested. (**E**) Distribution of Jaccard similarities $J(z_{n},z_{\text{mix}})$ of the various models, across all backgrounds and odor samples tested.

We now develop biologically plausible models of olfactory habituation that rely on manifold learning. Following [36, 42], we formulate a mathematical description (Fig. 2A) of the early olfactory circuit (Fig. 1C). We use *Drosophila* cell types for conciseness, but the model generalizes to other organisms. The key component for habituation is a layer of $N_{I}$ lateral interneurons (LN) which receive inputs s from olfactory sensory neurons (OSN) via synaptic weights M and are coupled with lateral connections L, thus having activities $\bar{h}=LMs$. OSNs excite projection neurons (PNs) with unit synaptic weights and LNs inhibit PNs with synaptic weights W. The net activity of the PNs is thus $y=s-W\bar{h}$.

Intuitively, in our model, interneurons learn to project inputs onto the low-dimensional subspace of background odors, and subtract these projections from PNs. Interneurons can therefore perform (linear) manifold learning with projection matrix WLM. Lastly, PN activities are projected on a large layer of $N_{K}$ Kenyon cells (KC) by sparse random connections (fixed, not learned). The condition $N_{K}\gg N_{S}$ ensures that the 5 % most active KCs represent a distinct neural tag z for each possible odor. This dimensional expansion from PNs to KCs implements locality-sensitive hashing of input identity [42].

Next, we consider biologically realistic synaptic plasticity rules for weights M, L, and W to achieve adequate manifold learning within this network. The optimal manifold projection matrix P derived for Fig. 1E involves non-local terms and moments of the new odor distribution inaccessible to the network (eq. 9). Instead, we postulate a simple, unsupervised, local learning rule for the inhibitory weights W: they evolve during habituation to minimize the norm of the PN activity y by using interneuron activities $\bar{h}$. This optimization principle results in simple Hebbian dynamics (see Methods),

(3) $$\frac{dW_{ij}}{dt}=\alpha y_{i}{\bar{h}}_{j}-\beta W_{ij}$$

with learning rate α and a regularization rate β. This simple update rule allows us to compare different models for the projection weights M and L. We consider two models: (a) the Intrator, Bienenstock, Cooper, and Munro (IBCM) model of synaptic plasticity [43-46], and (b) a biologically plausible online implementation of principal components analysis (BioPCA) [47] (for full model definitions, see Methods). The IBCM model was proposed to explain neuronal selectivity to specific stimulus components [43]. Its connections with independent component analysis (ICA) [48, 49] suggest that it could provide a biologically meaningful basis for learning the manifold of non-Gaussian backgrounds.

We perform initial numerical simulations, outlined in Fig. 2B, to assess the performance of different habituation schemes. We compare these rules to the average subtraction mechanism proposed in [36] (“Average”), as well as with the absence of habituation (“None”) and the optimal manifold learning matrix P derived in the previous section (see Methods). The dynamical equations for each plasticity rule are integrated in the presence of a background as in Eq. (1), with concentrations fluctuating according to the turbulent stochastic process of Fig. 1A-B. After this habituation period, we present the network with mixtures of the background and new odors, $s_{\text{mix}}=s_{b}(t)+s_{\text{new}}$. To assess how well that odor is decoded from the mixture, we compare its output with the neural tag $z_{\text{new}}$ of the new odor alone.

We find that while average subtraction cannot inhibit the strong fluctuations of turbulent backgrounds (Supp. Materials, sec. 3), both the IBCM and BioPCA networks significantly reduce PN activity in response to the background (Fig. 2C) comparably to the optimal manifold learning matrix P. This confirms that both models provide adequate projections on the background subspace, allowing the Hebbian rule for W (Eq. (3)) to achieve its function of minimizing PN activity.

Moreover, we compare the models’ performance for new odor recognition after habituation at the level of PN activities (Fig. 2D) and neural tags (Fig. 2E). For both metrics, average subtraction provides a very limited improvement compared to recognition without habituation, due to the strong background fluctuations caused by turbulence. In contrast, manifold learning implementations significantly improve odor recognition, with both IBCM and BioPCA networks performing similarly well. With respect to the distance in PN activity between the new odor alone and mixed with the background (Fig. 2D), these models result in a ~ 3-fold improvement, but fall short of the optimum by a similar factor. This is not surprising, since P is fine-tuned for the distribution of new odors, which is unknown to the IBCM and BioPCA networks. Nonetheless, in terms of the Jaccard similarity between neural tags of the mixture and the new odor (Fig. 2B, right), the IBCM and BioPCA networks perform within 15 % similarity of the optimum (Fig. 2E), producing responses much more similar to the new odor than to background odors (Fig. S2). These models thus recover, from a mixture with the background, roughly 50 % of the KCs which are most activated by the new odor alone and define its identity, even when the new odor is present at just half the average whiff concentration (Fig. 2E, left). These results prompt us to investigate in more detail how the IBCM and BioPCA models learn the background manifold.

### Analysis of background habituation by the IBCM model.

**Fig. 3.**
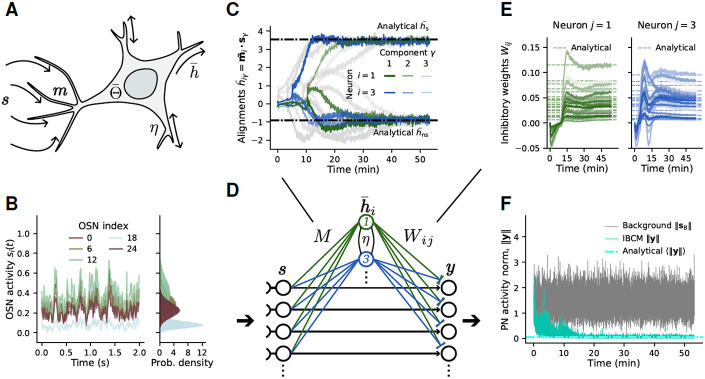
The IBCM model of synaptic plasticity learns olfactory background components. (**A**) Illustration of an IBCM neuron’s inputs from OSNs, s, synaptic weights m, activity $\bar{h}$, and internal threshold $\bar{\Theta }$. (**B**) OSN activities for a simple background process with 3 odors and weakly non-Gaussian concentration fluctuations. (**C**) Time series of the IBCM neurons’ alignment with the different background odors (two neurons are highlighted in green and blue, with line widths indicating different odors γ). Each neuron predominantly aligns with one odor (large dot product value ${\bar{h}}_{s}$ for one odor, small value ${\bar{h}}_{\text{ns}}$ for the others). This steady-state alignment matches our analytical predictions (dashed lines, Supp. Materials sec. 4). (**D**) Location, in the olfactory network model, of the different weights and neurons illustrated in other panels. (**E**) Time series of the IBCM neurons’ inhibitory synaptic weights, W, for the neurons highlighted in (C). The steady-state values of these weights can also be derived analytically (dashed lines) and align well with the simulations. (**F**) Norm of the response of projection neurons (PN) to the background process during habituation. The analytical prediction (dashed line) over-estimates the actual inhibition, since it neglects the contribution of fluctuations in M and W.

We first focus on the IBCM model, since its mechanisms are less intuitive than PCA and have classically been characterized for visual input processes alternating between a fixed set of vectors [44, 50]. In its simplest form (Fig. 3A), the IBCM model describes the slow variation of a neuron’s synaptic input weights m (a row in matrix M) as

(4) $$\frac{dm}{dt}=\mu h(h-\Theta )s(t)\;\;\text{where}\;h=m\cdot s(t)\;\frac{d\Theta }{dt}=\frac{1}{\tau_{\Theta }}(h^{2}-\Theta )$$

where h is the activity in response to input s(t), μ is the learning rate, and Θ is an internal threshold converging to a temporal average $\Theta =\langle h^{2}\rangle (\langle \cdot \rangle$ denotes averages over the input fluctuations). In practice, we include lateral mean-field inhibition between interneurons with coupling parameter η, a mild nonlinearity to h preventing excessively large activations, and a small (ε≪1) decay term −εμm. For simulations with turbulent backgrounds, we use a variant of the model from [45] where the learning rate is divided by Θ to speed up convergence (see Methods).

The m equation introduces a competition between input patterns: inputs that cause h>Θ are further reinforced, while sub-threshold ones are further depressed; consequently, a neuron responds specifically to some inputs and does not respond to others [43]. This mechanism works when the threshold time scale $\tau_{\Theta }$ is slow enough to average over fast input fluctuations s(t), yet still fast compared to the learning rate μ. This separation of time scales is to ensure m does not vary much while Θ averages over fluctuations; oscillations arise in the synaptic weights [51] without the separation $\tau_{\Theta }\ll 1∕\mu$. This criterion can nonetheless be achieved during olfactory habituation over the course of 30-60 minutes.

To gain insight into how IBCM neurons learn the background subspace, we examine the fixed point equations of the model averaged over fast input fluctuations (Supp. Materials, sec. 4), finding exact expressions for these solutions in terms of the background concentration moments. From a linear stability analysis (Supp. Materials, sec. 4F and Fig. S3), we find that the only stable fixed points are those where the alignment of the synaptic weights with background odor vectors, $h_{\gamma }=m\cdot {\hat{s}}_{\gamma }$, take a large positive value $h_{\text{sp}}$ (“specific”) for *one* odor γ and a small, possibly negative value $h_{\text{ns}}$ (“non-specific”) for all other background components. Hence, an IBCM neuron learns to selectively respond to one background odor: the classical specificity property of this model [44] thus extends to quite general input stochastic processes of the form given in eq. 1. From the solutions for m and h, we also derive analytical expressions for the fluctuation-averaged inhibitory W weights at steady-state (Supp. Materials, sec. 4G). Overall, our results show that a network of IBCM neurons performs manifold learning by having each neuron selectively suppress one background odor. Lateral inhibitory coupling between IBCM neurons (matrix L) help to push each neuron towards a different odor component [52].

To confirm our analysis, we perform numerical simulations with a simpler, weakly non-Gaussian background (Fig. 3B, see Methods). As expected, each IBCM neuron evolves over time to align with one background component (Fig. 3C,D), with steady-state average values of the dot products ${\bar{h}}_{\gamma }$ closely matching our analytical predictions $h_{\text{sp}}$, $h_{\text{ns}}$ (dashed lines). Different IBCM neurons (labeled by colors) become specific to different odors (line transparencies). The W weights also converge to steady-state average values matching our analytical results (Fig. 3E). The network of IBCM neurons performs habituation effectively, reducing both the mean and standard deviation (fluctuations) of the PN activity norm below ~ 10% of the input levels. Characterizing further the learning dynamics, we observe that the selectivity of IBCM neurons is acquired in two phases, first approaching a saddle point before converging to a selective fixed point (Supp. Materials sec. 6, Fig. S4, and Fig. 3C). This selectivity is driven by skewness (non-zero third moment) in the background statistics [44, 53] (Fig. S5).

**Fig. 4.**
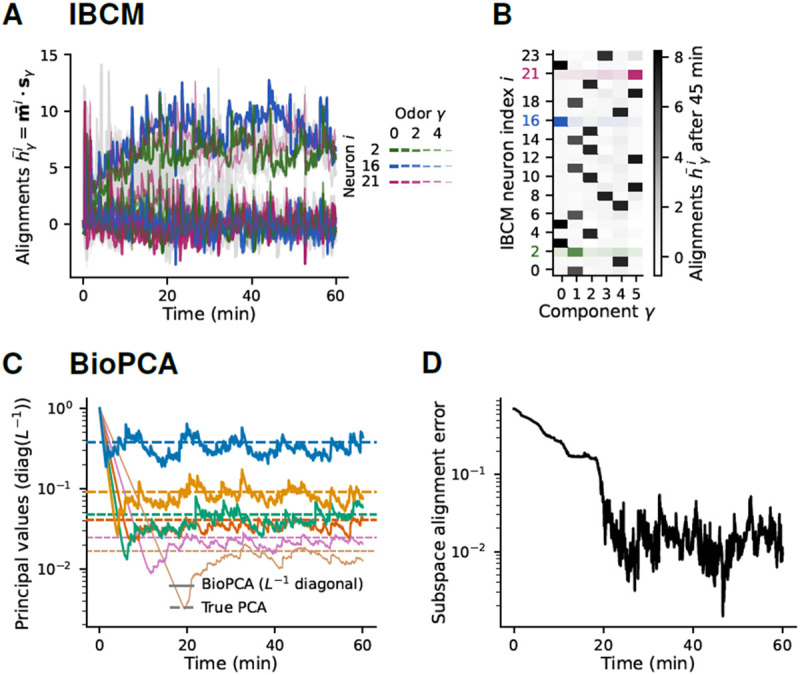
Habituation of IBCM and BioPCA neurons to turbulent olfactory backgrounds. (**A**) Time series of the IBCM neurons’ synaptic weight alignment with background odor during habituation to a six-odor background with the turbulent concentration stochastic process illustrated in Fig. 1A-B. Three neurons are highlighted with colors. (**B**) Table of each neuron’s alignment after habituation, showing that IBCM neurons becomes selective for one odor even in this strongly fluctuating background. (**C**) Time series of the principal values learned by lateral interneurons obeying the BioPCA model during habituation to the same turbulent background. These principal values are stored in the inverse of the self-coupling weights $L_{ii}^{-1}$ (inverse of the diagonal entries in the LN coupling matrix L). The principal values learned by the first $N_{B}$ neurons converge to averages equal to the $N_{B}$ non-zero eigenvalues of a PCA decomposition of the background (dashed horizontal lines). (**D**) Alignment error between the background subspace and the principal components learned by the BioPCA LNs (the rows of the LM matrix should be the principal components), confirming the model does learn the PCA decomposition of the background.

Importantly, these properties of IBCM neurons are robust across different background statistics. We observe similar specificity and learning dynamics in IBCM neurons exposed to log-normal background fluctuations (Fig. S6) and in the simulations of Fig. 2 with the full turbulent statistics (Fig. 4A-B). In the latter case, averaging over long whiffs and blanks requires slower learning rates $1∕\tau_{\Theta }$ and μ (see Methods and Table S2); despite stronger fluctuations, IBCM neurons align with a single background odor each.

### Comparison of IBCM and BioPCA learning.

As a point of comparison, we also analyze how the BioPCA network learns the background manifold in fluctuating environments. Despite strongly non-Gaussian statistics, the network converges to the expected PCA decomposition fixed point (see Methods, Supp. Materials, sec. 5 and sec. 6C). The matrix L becomes nearly diagonal, containing the principal values (Fig. 4C, Fig. S6B), while the rows of M converge to (scaled versions of) the principal component vectors, as evidenced by the error on their alignment decreasing to ~ 1 % (Fig. 4D, Fig. S6C, Fig. S7).

On the whole, both models achieve similar habituation levels within ~ 30 minutes. However, they rely on distinct mechanisms and converge to different vector bases for the background manifold. BioPCA neurons learn principal components: linear combinations of the true odors, distinguished by their variance. IBCM neurons, in contrast, rely on higher statistical moments of the inputs to select individual odor sources. Both models require in principle one neuron per background dimension, $N_{I}=N_{B}$, to span the background subspace. Superfluous neurons have little effect for the BioPCA model, where they reach principal values $L_{ii}\approx 0$. For the IBCM network, extra neurons are helpful, increasing the probability that each background odor will be selected by at least one of them. Notwithstanding these differences, both models produce very similar habituation and odor recognition performances in Fig. 2. They are also similar in their robustness to OSN noise (Fig. S8), and in their performance when combined with alternate Hebbian rules for the W weights based on different $L^{P}$ norms (Supp. Materials sec. 7 and Fig. S9).

### Performance in various olfactory space conditions.

**Fig. 5.**
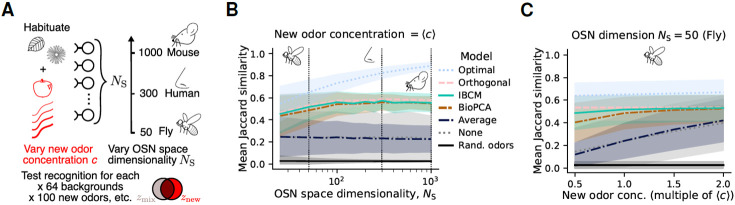
Recognition performance as a function of olfactory space dimensionality and new odor concentration. (**A**) For various olfactory space dimensionalities $N_{S}$ (*i.e.*, number of OSN types) and new odor concentrations $c_{n}$ at test times, we perform simulations like those in Fig. 2: the model habituates to a six-odor turbulent background for ~60 minutes before a new odor is introduced in the mixture. Each ($N_{S}$, $c_{\text{new}}$) condition is tested across 64 backgrounds, 100 new odors, 5 test times post-habituation, and 4 background samples at each test time. (**B**) New odor recognition performance, quantified by the Jaccard similarity between the new odor and the response to the mixture after habituation, as a function of $N_{S}$, for different manifold learning models. Results shown here are for the new odor $c_{\text{new}}$ equal to the average concentration of background odors, 〈c〉 (see Fig. S10 for all concentrations). “Optimal”: manifold learning matrix W derived in Fig. 1E. “Orthogonal”: similarity between the entire new odor and its component orthogonal to the background. “Rand. odors”: similarity between two randomly selected odors (*i.e.,* similarity by chance). The shaded area represents one standard deviation across replicates. (**C**) Recognition performance as a function of the concentration at which the new odor is presented (multiples of 〈c〉), for the fly case ($N_{S}=50$; see Supp. Materials, Fig. S10A for all dimensions). Same legend as (B).

To understand the similar performance of the IBCM and BioPCA versions of manifold learning, we investigate the effect of various olfactory space parameters on them. We perform numerical simulations analogous to those of Fig. 2B for increasingly large olfactory space dimensions ($N_{S}$) and for a wider range of new odor concentrations (Fig. 5A). We consider dimensionalities ranging from half (25) that of the fruit fly (50) up to human (300) and mouse (1000) levels. While the performance of the optimal manifold learning algorithm increases with $N_{S}$ up to a nearly perfect score, the IBCM and BioPCA networks reach a very similar plateau at $N_{S}∼100$ (Fig. 5B). Remarkably, this plateau corresponds to the similarity between the new odor tag, $z_{\text{new}}$, and the tag $z_{\text{new},⊥}$ of the new odor component orthogonal to the background, $y_{\text{new},⊥}$ (“orthogonal” pink line, Fig. 5B). This observation clarifies why both models perform similarly well: the local rules for W (Eq. (3)), based on minimizing PN activity, cause the parallel component of the new odor to be subtracted at the same time as the background. As long as the M weights provide complete projections of the inputs, the network produces a response $y_{\text{mix}}\approx s_{\text{new},⊥}$. In comparison, the optimal matrix P preserves some of the new odor’s parallel component, thus maximizing the recognition of $s_{\text{new}}$.

Still, the levels of odor recognition reached by the IBCM and BioPCA models are significant, several standard deviations above chance similarity (black line, Fig. 5B). They also perform well above the average subtraction model, which was similar to no habituation: in that model, new odor signals are masked by strong fluctuations away from the average. For higher new odor concentrations, manifold learning provides a more modest improvement (Fig. 5C and Fig. S10), because habituation is not as crucial for very strong new odor whiffs which dominate the background. Overall, both IBCM and BioPCA interneurons subtract the background manifold from olfactory inputs while preserving new odor signals in regimes that are physically and computationally relevant for biological systems.

## Discussion

Sensory adaptation to olfactory backgrounds is particularly challenging due to strong fluctuations generated by turbulent mixing in naturalistic conditions. We showed that predictive filtering strategies, which act on individual stimulus features, cannot adequately distinguish between changes in activity due to new odors and changes in activity due to fluctuations in the background. An alternative class of habituation strategies, manifold learning, could better identify new odors by learning to subtract projections of the instantaneous inputs onto the low-dimensional background manifold. We propose that inhibitory interneurons, which modulate the activity of principal neurons in early olfactory pathways, implement a manifold learning strategy for habituation. We explore two classes of synaptic plasticity rules, each of which combines a Hebbian-like rule and a linear projection learning rule (IBCM or BioPCA). Our analysis shows that these simplified linear manifold learning strategies are near-optimal for a range of physiologically relevant parameters, including when background odors display strong fluctuations such as those encountered in turbulent environments. Both plasticity rules show comparable performance on a habituation task, but learn distinct stimulus features. Notably, IBCM neurons select biologically relevant projections corresponding to independently varying components in the background mixture.

The biological underpinnings of our proposed model for olfactory background manifold projection are supported by previous experimental and theoretical studies. All connections in our network structure (OSN to PN, OSN to LN, LN to PN) are abundant in the connectome [12]. Habituation on the time scale of minutes has been shown to occur predominantly at the level of PNs in flies [22, 54] or M/T cells in mice [29]. Several studies found lateral inhibitory signals (GABA, glutamate) and their receptors for such signals (GABA-A, NDMA) to be essential to habituation [6, 27, 34, 35]. Our model relies on odor-specific PN inhibition by PN-to-LN plasticity for habituation, as observed in *Drosophila* [8] and honeybees [55]. Of note, we neglected feedback of PNs on LNs [31] and instead considered a feedforward network for mathematical simplicity, to illustrate our concept of manifold learning. Moreover, recent theoretical work has argued that the PN-LN connectivity pattern reflects correlations in PN activity, suggesting that the PN-LN circuit whitens odor representations in the antennal lobe [56]. However, the authors focused on hardwired computations preadapted to a given set of odors (*i.e.,* offline), whereas we addressed a different problem altogether, showing that online PCA is one plausible set of synaptic plasticity rules to achieve background subtraction in fluctuating environments.

Our theory provides salient predictions that could be tested experimentally. The simplest observable feature is the decrease in both the mean and variance of PN (or M/T cells in mice) activity after 20-60 minutes of exposure to turbulent odor mixtures (Fig. 2C and 3F). This phenomenon has already been observed for simpler backgrounds in *Drosophila* [8], which motivated our study. It could be directly tested in mice by calcium fluorescence imaging of glomeruli [37]. PN or glomerular activity should however be restored in response to new odors orthogonal to the learned background. In comparison, temporal average filtering would fail to reduce PN activity (Fig. 2C), while filtering based on recent samples (as in eq. 2) would rapidly suppress the response to new odors as well.

A more subtle feature in our proposed model is that lateral interneuron activity (LN) should, conversely, closely track background stimuli in real time to keep inhibiting PN responses (Fig. S8D-E). It may be experimentally challenging, however, to single out interneurons and record their fast fluctuations. A corollary of this model feature (which might be easier to measure) is that, since LN activity reflects projections on the learned background manifold, these neurons should become silent if the stimulus is suddenly switched to new odors with null projections on the previous subspace. Their activity should slowly recover on the time scale of habituation as they learn the new background.

A third feature of habituation by manifold learning is that new odor recognition performance decreases with the distance to the background subspace (*i.e.,* as the norm of the orthogonal component $s_{n,⊥}$ decreases; Fig. S2C-D). This dependence would not be as strong in predictive filtering strategies. This correlation between odor recognition and distance from new odors could be tested in behavioral experiments.

Further theoretical work will also be necessary to refine our proposed implementation of manifold learning in olfactory circuits, and to assess how this strategy may be coupled with other odor recognition mechanisms. Schemes more sophisticated than a Hebbian rule would be necessary to reach the optimal performance promised by manifold learning (Figure 5) or to fully exploit the biologically relevant projections learned by IBCM neurons. Also, in our study, we focused on linear background manifolds (eq. 1) that could be decomposed into linear projections (h=LMs); while manifold projection also applies conceptually to non-additive odor mixtures, this extension will require olfactory circuit implementations of algorithms for curved manifold learning, such as manifold tiling [57]. Moreover, by choosing to focus on early olfactory processing, we neglected neuromodulatory inputs [58-60] and feedbacks from higher-level cognitive functions [61, 62]. For instance, while we assumed background and new odors are merely defined by their order of presentation, long-term memory of odors and other computations in the piriform cortex [63] likely help mammals focus their attention on relevant cues rather than on uninformative odors for, *e.g.*, odor trail tracking [64, 65]. Future investigation on this aspect could draw upon recent advances on attention mechanisms in artificial learning models [66]. Conversely, the concept of background manifold projection could prove useful for algorithms performing figure-ground segregation in time-varying signals, such as in video object detection [67].

Beyond olfaction, the interplay between habituation and attention also arises in other biological systems performing chemodetection in fluctuating environments [68]. For instance, in T cell antigen recognition [69], both (immune) memory and (T cell receptor) signal processing networks play important roles for pathogen detection amid a sea of irrelevant (self) antigens. Overall, we hope that our proposed model of habituation via manifold learning will motivate further theoretical and experimental efforts to clarify how living systems meet the challenge of adaptation to fluctuating backgrounds.

## Materials and Methods

### Odor vectors and concentrations.

In our models, odors have a fixed, unit-normed direction, and an amplitude along that axis set by their (fluctuating) concentration: $s(t)=c\hat{s}$. Except for the idealized setup of Fig. 1E, vectors for background (${\hat{s}}_{\gamma }$) and new (${\hat{s}}_{\text{new}}$) odors are drawn from the same distribution ${𝒫}_{\hat{s}}$, by sampling i.i.d. exponential elements, then normalizing each vector. New odors are tested at fixed concentrations $c_{\text{new}}$. Background concentrations $c_{\gamma }(t)$ follow a stochastic process, usually (Figs. 2, 5) the turbulent process illustrated in Fig. 1A-B. We simulate each odor concentration as a telegraph-like process, alternating blanks and whiffs with stochastic durations and whiff concentrations. The power-law distribution of whiffs and blanks durations ($t_{w}$, $t_{b}$) have a lower cutoff at 10 ms and upper cutoffs at 5 s (whiffs) or 8 s (blanks), respectively. The whiff concentration distribution has a scale $c_{0}=0.6$. We also considered weakly non-Gaussian (Fig. 3) and log-normal (Fig. S6) background concentrations, by simulating a multivariate Ornstein-Uhlenbeck {$g_{\gamma }(t)$}, then transforming these variables as $c_{\gamma }(t)=g_{\gamma }(t)+\nu g_{\gamma }{\left(t\right)}^{2}$ or $c_{\gamma }(t)=10^{g_{\gamma }(t)}$, respectively. We used a short autocorrelation time τ=20ms, an average $\langle g\rangle =1∕\sqrt{N_{B}}$. and standard deviation $\sigma_{g}=0.3$. For the non-Gaussian case, we set ν=0.2. Details of the stochastic simulation methods are provided in Supp. Materials sec. 2.

### Optimizing predictive filtering and manifold learning regimes.

In Eq. (2), we introduced an idealized inhibitory network response combining manifold learning and predictive filtering. The objective of this network is to minimize the squared distance between its response, $b(T)+s_{n}-u(T)$, and the target odor alone, $s_{n}$. The corresponding loss is

(5) $${ℒ}_{v,P}=\langle {\left\|b(T)-\sum_{j=1}^{T-1}v_{j}b(T-j)-P(b(T)+s_{\text{new}})\right\|}^{2}\rangle ,$$

where the average is taken across samples (concentrations) from a given background and across new odors. Our goal was to determine the ideal performance of such a network, and the contribution of each habituation strategy depending on olfactory space parameters. We therefore solved for the optimal scalar coefficients $v_{j}$ and the optimal $N_{S}\times N_{S}$ matrix P, as an upper bound on the mechanisms that real networks could learn during habituation. We minimized the loss by solving $\frac{\partial ℒ}{\partial v_{j}}=\frac{\partial ℒ}{\partial P_{ij}}=0$. Supp. Materials sec. 1 details the calculation and the result; we obtained a general solution for any background mixture, as defined in Eq. (1), considering zero-mean and statistically independent concentrations.

For Fig. 1E, we considered a simpler particular case where background odors are orthogonal, new odors are drawn uniformly from the unit hypersphere, and background concentrations have an exponential autocorrelation function with time constant τ, $\langle c_{\gamma }(t)c_{\rho }(t+s)\rangle =\sigma^{2}\delta_{\gamma \rho }e^{-|s|∕\tau }$. The minimized loss is then

(6) $${ℒ}_{v,P}=N_{B}\sigma^{2}\left(1-e^{-2∕\tau }\right)∕(1+{\tilde{N}}_{S}(1-e^{-2∕\tau }))$$

where ${\tilde{N}}_{S}=(\sigma^{2}∕\sigma_{n}^{2})N_{S}$ and $\sigma_{n}^{2}$ is the new odor concentration variance. In the figure, we compared ${ℒ}_{v,P}$ with the limiting cases of pure predictive filtering (${ℒ}_{v}$, setting P=0) and pure manifold learning (${ℒ}_{P}$, setting $v_{j}=0$), which respectively give losses

(7) $${ℒ}_{v}=N_{B}\sigma^{2}(1-e^{-2∕\tau })\;,\;{ℒ}_{P}=N_{B}\sigma^{2}∕(1+{\tilde{N}}_{S})\;.$$

For the “optimal” strategy in Figures 2 and 5, we needed to derive the general solution (for non-orthogonal background and new odors drawn from ${𝒫}_{\hat{s}}$) for pure manifold learning (v=0) when the background has a non-zero average (as was the case in our simulations). In that case, the optimal projection matrix is

(8) $$P=(\langle {\mathrm{bb}}^{⊺}\rangle +\langle b\rangle \;{\langle s_{\text{new}}\rangle }^{⊺})\;M^{+}$$

where $M^{+}$ is the Moore-Penrose pseudo-inverse (or the usual matrix inverse, when it exists) of

(9) $$M=\langle {\mathrm{bb}}^{⊺}\rangle +\langle s_{\text{new}}s_{\text{new}}^{⊺}\rangle +\langle b\rangle \;{\langle s_{\text{new}}\rangle }^{⊺}+\langle s_{\text{new}}\rangle \;{\langle b\rangle }^{⊺}\;.$$

We evaluated numerically the moments $\langle {\mathrm{bb}}^{⊺}\rangle$, $\langle s_{\text{new}}s_{\text{new}}^{⊺}\rangle$, etc., by sampling unit vectors $s_{\gamma }$, $s_{\text{new}}$ from ${𝒫}_{\hat{s}}$ and background concentrations $c_{\gamma }$ from the stationary distribution of the background process at hand – for Figs. 2 and 5, the turbulent statistics shown in Fig. 1A-B.

### Mathematical model of the olfactory network.

We model the instantaneous response of the olfactory network (Fig. 2A) to a stimulus s(t) received at time t as the following set of neural activities in its different layers:

(10) $$\bar{h}(t)=\phi (LMs(t))\;\;(\text{interneurons})$$

(11) $$y(t)=s(t)-W\bar{h}(t)\;\;(\text{PNs})$$

(12) $$z(t)=5\%\;\text{most active in}\;R_{\theta }(Qy(t))\;\;(\text{KCs})$$

where ϕ is an element-wise nonlinearity and $R_{\theta }$ clips elements below threshold θ. We used $\phi (x)=A_{\text{sat}}\;\tanh(x∕A_{\text{sat}})$ for IBCM neurons to saturate their activity at a large $A_{\text{sat}}=50$ for numerical stability; most of the time, $x\ll A_{\text{sat}}$ is in the linear part of this function, so y≈s−WLMs. We did not apply a nonlinearity for the BioPCA network, nor for the IBCM model on simpler backgrounds (Fig. 3).

Then, the neural tag z is computed as in [36]. First, PN activities are projected to the KC layer by the sparse $N_{K}\times N_{S}$ binary matrix Q. Then, $R_{\theta }$ clips Kenyon cells (KCs) with activity below threshold; we set $\theta =\frac{1}{60}\times fN_{S}\times \langle s_{i}\rangle$, where $\langle s_{i}\rangle$ is the average OSN activity in the current input s(t) and f=6∕50, the fraction of PNs forming a synapse with one KC in *Drosophila.* Finally, the neural tag z is the set of all non-zero KCs with activity above the 95th percentile of all KC activities.

The matrix Q is generated by randomly picking $fN_{S}$ PNs to project to each KC (*i.e.,* picking $fN_{S}$ non-zero elements in Q’s row for that KC). We generated a new Q for each background tested in the numerical experiments in Figs. 2 and 5. When varying the olfactory space dimensionality $N_{S}$ in Fig. 5, we preserved the relative size of PN and KC layers $N_{S}∕N_{K}=50∕2000$ found in *Drosophila*; hence, for mice with $N_{S}=1000$ OR types, we used $N_{K}=40,000$ cortical cells (KC equivalent), and the Q matrix had $fN_{S}=120$ M/T cells (PN equivalent) projecting to each cortical cell. This ratio aligned with experimental estimates in mice giving ~ 200 glomeruli connected to a cortical cell, or 10 % sparsity in Q [70-72]. The other matrices (M, W, L in BioPCA) are slowly updated according to synaptic plasticity rules during a habituation run.

### Hebbian learning rule for W.

The W weights are learned according to the Hebbian rule in Eq. (3). This rule derives from minimizing the average squared PN activity with $L^{2}$ regularization on the $W_{ij}$:

(13) $${ℒ}_{W}=\frac{1}{2}\;\langle y^{2}\rangle +\frac{\beta }{2\alpha }\sum_{i,j}W_{ij}^{2}$$

where we recall that $y=s-W\bar{h}$. Taking the W dynamics to be a gradient descent on ${ℒ}_{W}$ with rate α, $\frac{dW_{ij}}{dt}=-\alpha \frac{\partial {ℒ}_{W}}{\partial W_{ij}}$, yields the aforementioned Hebbian rule. The average 〈〉 is replaced by time averaging over a time window 1∕α using a slow rate α to implement online averaging of fast background fluctuations.

### Average subtraction model.

The “negative image” subtraction model proposed in [36] is effectively a mean filtering or average subtraction model. It can be recast in the form of our network structure by having $N_{I}=1$ interneuron with fixed activity h=1, without M or L weights. The W weights are then a vector $w_{\text{avg}}$, which is subtracted from the PN response input since h=1: $y(t)=s(t)-w_{\text{avg}}$. The Hebbian rule above is then

(14) $$\frac{dw_{\text{avg}}}{dt}=\alpha (s(t)-w_{\text{avg}})-\beta w_{\text{avg}}\;,$$

which makes $w_{\text{avg}}$ align, at steady-state, with the average of the background over a time window, $w_{\text{avg}}=\frac{\alpha }{\alpha +\beta }\langle s\rangle$ (Supp. Materials, sec. 3 for details).

### Network of IBCM neurons.

Equation 4 presents the simplest form of the IBCM model for a single neuron. In our olfactory network, we consider $N_{I}$ IBCM neurons with constant mean-field lateral inhibitory coupling, as proposed in [44], corresponding here to a matrix L with 1 on the diagonal and −η off-diagonal. Consequently, the reduced activity ${\bar{h}}_{i}$ of neuron i (*i.e.*, element i of $\bar{h}=\phi (LMs)$) is

(15) $${\bar{h}}_{i}=\phi \left(m_{i}\cdot s(t)-\eta \sum_{j\neq i}m_{j}\cdot s(t)\right)=\phi ({\bar{m}}_{i}\cdot s(t)),$$

where we defined the inhibited weights ${\bar{m}}_{i}=m_{i}-\sum_{j\neq i}m_{j}$, and where ϕ is the tanh nonlinearity introduced in Eq. (10). The complete dynamical equation for the synaptic weights $m_{i}$ incoming into IBCM neuron i has additional terms due to this coupling,

(16) $$\frac{dm_{i}}{dt}=\mu_{{\bar{\Theta }}_{i}}{\bar{h}}_{i}\;({\bar{h}}_{i}-{\bar{\Theta }}_{i})\;\phi^{\prime }({\bar{m}}_{i}\cdot s(t))s(t)\;\;-\eta \sum_{j\neq i}\mu_{{\bar{\Theta }}_{j}}\;({\bar{h}}_{j}-{\bar{\Theta }}_{j})\;\phi^{\prime }({\bar{m}}_{j}\cdot s(t))s(t)-\varepsilon \mu m_{i}\;,$$

where $\phi^{\prime }$ is the derivative of the nonlinearity. We have also added a small decay term $-\varepsilon \mu m_{i}$ to eliminate any component orthogonal to the background manifold in the random initial weights. For simulations with turbulent background statistics, we scaled the learning rate in the first two terms as

(17) $$\mu_{{\bar{\Theta }}_{i}}=\frac{\mu }{{\left({\bar{\Theta }}_{i}\right)}^{2}+k_{\theta }^{2}}\;.$$

This form is similar to the variant introduced in [45], but we added a constant $k_{\Theta }$ in the denominator to prevent blowups at t=0, where we initialize $\bar{\Theta }=0$. For simpler backgrounds (Figs. 3, Fig. S6), we did not include this variant and simply used $\mu_{\Theta }=\mu$. Moreover, the internal threshold of each neuron, ${\bar{\Theta }}_{i}$, evolves as

(18) $$\frac{d{\bar{\Theta }}_{i}}{dt}=\frac{1}{\tau_{\Theta }}({\left({\bar{h}}_{i}\right)}^{2}-{\bar{\Theta }}_{i})$$

such that it tracks the reduced neuron activity ${\bar{h}}_{i}$ averaged on an intermediate time scale $\tau_{\Theta }$.

### Network of BioPCA neurons.

We could use the “inverse-free PSP” version of the biologically plausible online PCA (BioPCA) proposed in [47] directly for the M and L weights of the interneuron layer. The model converges to a fixed point where the L matrix is diagonal with the principal values in it, and where the matrix LM contains the principal vectors in its rows, with norms specified by the pre-defined diagonal matrix $\underline{\Lambda }$ [47, Lemma 3]. The model specifies dynamical update rules for M and $L^{\prime }=L^{-1}$, the inverse of L, rather than L directly. To avoid non-biological matrix inverse computations, the vector of interneuron activities h is computed with a Taylor series for L,

(19) $$\bar{h}(t)=\left({L^{\prime }}_{d}^{-1}-{L^{\prime }}_{d}^{-1}L_{o}^{\prime }{L^{\prime }}_{d}^{-1}\right)Ms(t)\approx LMs\;,$$

where $L_{\;d}^{\prime }$ contains the diagonal of $L^{\prime }$, and $L_{\;d}^{\prime }$ contains the off-diagonal terms. This approximation is accurate at the fixed point where $L^{\prime }$ is diagonal ($L_{o}^{\prime }\to 0$). The BioPCA dynamical update rules converging to this PCA decomposition are

(20) $$\frac{dM}{dt}=\mu_{M}\left(\bar{h}\;s^{⊺}-M\right)$$

(21) $$\frac{dL^{\prime }}{dt}=\mu_{L}\left(\bar{h}\;{\bar{h}}^{⊺}-\underline{\Lambda }L^{\prime }\underline{\Lambda }\right)$$

In practice, we set ${\underline{\Lambda }}_{kk}=\Lambda (1-\frac{\lambda_{r}(k-1)}{\left(N_{I}-1\right)})$, where Λ is the scaling factor for M weights described below to make IBCM and BioPCA perform similarly, and $\lambda_{r}$ is the range of Λ values (between 0 and 1). We followed the original paper’s recommendation for the linear decrease of $\Lambda_{kk}$ with k and for setting $\mu_{L}=2\mu_{M}$ For further comparison with the original paper [47], note the following equivalence between our notation ↔ theirs: M↔W, $L↔M^{-1}$, s↔x, and $\bar{h}↔y$.

In our simulations, we added an extra interneuron applying the average subtraction mechanism described in Eq. (14), with β=0, upstream of the BioPCA model. This way, the BioPCA network learned the decomposition of the covariance matrix rather than of $\langle ss^{⊺}\rangle$, which still includes the average 〈s〉. This choice did not change the model performance, but made it more interpretable.

To measure the convergence of M’s columns to the PCA vectors, in Fig. 4D, we computed, at each time point, the subspace alignment error proposed by [47],

(22) $$E_{\text{Pro}}(M)={\text{min}}_{Q\in O_{N_{S}}}\frac{{\left\|FQ-U_{\text{PCA}}\right\|}_{\text{Frob}}^{2}}{{\left\|U_{\text{PCA}}\right\|}_{\text{Frob}}^{2}}$$

where columns of $U_{\text{PCA}}$ contains the $N_{B}$ PCA vectors with non-zero eigenvalues, $F={\left({\underline{\Lambda }}^{-1}LM\right)}^{⊺}$ contains the eigenvectors learned in the network’s projection weights, and ${\left\|U\right\|}_{\text{Frob}}^{2}=\text{Tr}(U^{⊺}U)$ is the Frobenius matrix norm. The Q matrix minimizing the distance to give the alignment error solves the so-called orthogonal Procrustes problem and is $Q=U_{F}V^{⊺}$ where $U_{F}$, V come from the SVD of $U_{\text{PCA}}F^{⊺}=U_{F}\Sigma V^{⊺}$ [73].

### Scaling parameter Λ for M weights.

In the BioPCA model, the scale parameter Λ in the $\underline{\Lambda }$ matrix (see below eq. 21) controls the magnitude of weights M. This scale influences the strength of habituation: larger M~Λ weights allow smaller W~1∕Λ weights that are less constrained by regularization (β term in eq. 13) and thus further reduce PN activity. We set Λ to the value necessary to achieve the same background reduction level as the IBCM network, as predicted by our analytical calculations for the IBCM fixed points and post-habituation PN activity; see Supp. Materials, sec. 8 for detailed expressions. Of note, we rescaled the $\mu_{L}$ rate to $\mu_{L}∕\Lambda^{2}$ in the BioPCA model (eq. 21) to preserve exactly the same learning dynamics for any Λ, just with M weights scaled up or down.

For comparison, we introduced a similar scale parameter $\Lambda_{\text{IBCM}}$ in the IBCM model, but we generally kept it equal to 1 (its implicit value by default), since that was sufficient to achieve complete background manifold projection (Fig. S11). Similar to BioPCA, scaling of the learning rate μ was required for $\Lambda_{\text{IBCM}}\neq 1$ (Supp. Materials, sec. 8 for details).

### Numerical simulations and model parameter values.

We integrated the stochastic differential equations of the network, with the background processes simulated as described above, using an Euler scheme with time step Δt=10ms. Below, we give rates in scaled units where this time step = 1.

By default, we performed simulations lasting 360, 000 time steps (1 hour) with $N_{S}=25$ dimensions, $N_{K}=40N_{S}$ Kenyon cells, $N_{B}=6$ background odors, $N_{I}=24$ IBCM neurons or $N_{I}=N_{B}$ BioPCA neurons. For W Hebbian learning, we used $\alpha =10^{-4}$ and $\beta =2\times 10^{-5}$. However, for Fig. 3 and Fig. S6, we used $N_{B}=3$, $N_{I}=6$ (IBCM), $\alpha =2.5\times 10^{-4}$, and $\beta =5\times 10^{-5}$.

For the IBCM weights, we used by default $\mu =1.25\times 10^{-3}$, $\tau_{\Theta }=1600$, $\eta =0.6∕N_{I}$, $k_{\Theta }=0.1$, ϵ=0.005, and A=50 as the maximum amplitude of the nonlinearity ϕ. For the simple background in Fig. 3, we used $\mu =1.5\times 10^{-3}$, $\tau_{\Theta }=200$, $\eta =0.5∕N_{I}$, we did not apply the ϕ nonlinearity or divide the learning rate by $k_{\Theta }+\Theta$. Also, for the simulations in higher dimensions in Fig. 5, we used a slower learning $\mu =7.5\times 10^{-4}$ and $\tau_{\Theta }=2000$. For the BioPCA model, we used by default $\mu =10^{-4}$, $\mu_{L}=2\mu$, and $\lambda_{r}=0.5$ (the range of ${\underline{\Lambda }}_{kk}$ entries). For Fig. 4, we fixed $\Lambda_{\text{PCA}}=8$ instead of the exact value making BioPCA and IBCM inhibit the background equivalently (described above). A full list of parameter definitions and values is provided in Tables S1 and S2.

The background process was initialized to a random sample from its stationary distribution. We initialized the W weights to zero, and the M weights to random i.i.d. normal samples with standard deviation 0.2 (or 0.3 for Fig. 4) for IBCM, or standard deviation $\Lambda_{\text{PCA}}∕\sqrt{N_{S}}$ for BioPCA. For the latter model, we initialized L to the identity matrix (as recommended in the original paper); for IBCM, we initialized $\bar{\Theta }$ to the value of $\bar{h}$ with the initial weights and background.

## Supplementary Material

1
