# Supplementary material for: Manifold learning for olfactory habituation to strongly fluctuating backgrounds
Source: bioRxiv. 2025 May 30:2025.05.26.656161. Preprint. [Version 1] doi: 10.1101/2025.05.26.656161 (PMC12154897; doi:10.1101/2025.05.26.656161)
Supplement: 1 [file NIHPP2025.05.26.656161v1-supplement-1.pdf]

bioRxiv preprint doi: https://doi.org/10.1101/2025.05.26.656161; this version posted May 30, 2025. The copyright holder for this preprint (which was not certified by peer review) is the author/funder, who has granted bioRxiv a license to display the preprint in perpetuity. It is made available under aCC-BY 4.0 International license.

Supplementary Materials

1. Optimal models of manifold learning and predictive filtering

In this section, we detail the optimization problem we solved to delineate regimes of predictive filtering and manifold learning, as shown in Fig. 1E and Fig. S1.

A. Definition of the loss function.

We want to minimize the loss function

$$\mathcal{L}_{v,P} = \left\langle \left\| \mathbf{b}_t - \sum_{l=1}^{t-1} v_l \mathbf{b}_{t-l} - P(\mathbf{b}_t + \mathbf{x}) \right\|^2 \right\rangle_{\mathbf{b}\sim\mathcal{P},\, \mathbf{x}\sim\mathcal{Q}} \tag{23}$$

as a function of the scalar coefficients v_l (predictive filtering) and of the matrix P (manifold learning). In this section, we call $\mathbf{b}_{t'}$ the background OSN input vector at time t', and $\mathbf{x}$ the new odor (appearing at time t), instead of $\mathbf{s}_{\mathrm{b}}$ and $\mathbf{s}_{\mathrm{new}}$. The background is a linear combination of pre-defined odor vectors, $\hat{\mathbf{y}}_\rho$, weighted by stochastic concentrations, $\tilde{c}_{\rho,t'}$, so $\mathbf{b}_{t'} = \sum_{\rho=1}^{N_{\mathrm{B}}} \tilde{c}_{\rho,t'} \hat{\mathbf{y}}_\rho$. For simplicity, the concentrations are assumed i.i.d. and stationary with mean zero, variance $\langle \tilde{c}_{\rho,t'} \tilde{c}_{\lambda,t'} \rangle = \sigma^2 \delta_{\rho\lambda}$, and autocorrelation function $\langle \tilde{c}_{\rho,t'} \tilde{c}_{\lambda,t'\pm s} \rangle = C(s)\delta_{\rho\lambda}$, with $C(0) = \sigma^2$; together, these concentrations statistics define the background vector distribution $\mathcal{P}$. The new odor $\mathbf{x}$ comes from some distribution $\mathcal{Q}$ we assume has zero mean and finite covariance matrix $\langle \mathbf{x}\mathbf{x}^\intercal \rangle$.

A.1. Remarks on notation.

In this calculation, sums and matrix products are applied over three different indices, denoting olfactory dimensions, time, and background odors, *e.g.*, $(P\mathbf{x})_i = \sum_j P_{ij} x_j$. To make notation more concise, we rewrite several sums as dot products. To clarify the indices on which these products are, we use boldface $\mathbf{x}$ on vectors in olfactory dimensions, and underlines $\underline{\ }$ ($\underline{\underline{\ }}$) for $\underline{\text{vectors}}$ ($\underline{\underline{\text{matrices}}}$) in time dimensions. We write out explicitly sums with Greek indices on background odor indices, $\sum_{\rho=1}^{N_{\mathrm{B}}}$.

A.2. Expanding the loss function terms.

With the above assumptions on the background and new odor statistics, we can expand the square and write out the different terms in the loss function. First, using the statistical independence and zero mean property of $\mathbf{x}$ and $\mathbf{b}_{t'}$, the terms to evaluate are

$$\begin{aligned}\mathcal{L}_{v,P} = &\langle \mathbf{b}_t{}^\intercal \mathbf{b}_t \rangle + \sum_{l,m} v_l v_m \langle \mathbf{b}_{t-l}{}^\intercal \mathbf{b}_{t-m} \rangle + \langle \mathbf{b}_t{}^\intercal P^\intercal P \mathbf{b}_t \rangle + \langle \mathbf{x}^\intercal P^\intercal P \mathbf{x} \rangle \\ &- 2\sum_{l=1}^{t-1} v_l \langle \mathbf{b}_t{}^\intercal \mathbf{b}_{t-l} \rangle - 2\langle \mathbf{b}_t{}^\intercal P \mathbf{b}_t \rangle + 2\sum_{l=1}^{t-1} v_l \langle \mathbf{b}_{t-l}{}^\intercal P \mathbf{b}_t \rangle \ .\end{aligned}$$

We compute these terms more explicitly by using the background statistics defined above. The loss function is thus

$$\begin{aligned}\mathcal{L} = &N_{\mathrm{B}}\sigma^2 + N_{\mathrm{B}} \sum_{l,m=1}^{t-1} v_l v_m C(l-m) + \sigma^2 \sum_{\rho=1}^{N_{\mathrm{B}}} \hat{\mathbf{y}}_\rho^\intercal P^\intercal P \hat{\mathbf{y}}_\rho + \langle \mathbf{x}^\intercal P^\intercal P \mathbf{x} \rangle \\ &- 2N_{\mathrm{B}} \sum_{l=1}^{t-1} v_l C(l) - 2\sigma^2 \sum_{\rho=1}^{N_{\mathrm{B}}} \hat{\mathbf{y}}_\rho^\intercal P \hat{\mathbf{y}}_\rho + 2\left(\sum_{l=1}^{t-1} v_l C(l)\right)\left(\sum_{\rho=1}^{N_{\mathrm{B}}} \hat{\mathbf{y}}_\rho^\intercal P \hat{\mathbf{y}}_\rho\right) \ .\end{aligned} \tag{24}$$

We did not need to assume that background odors were orthogonal to get this answer; the statistical independence of their concentrations $\tilde{c}_{\rho,t}$ removed cross-odor terms. Most terms involve only P or v; only the last term couples the two strategies together.

B. Solving for the optimal P and v.

B.1. Loss function derivatives and resulting optimum equations.

We can now take the derivative of this loss function with respect to the parameters v_j and P_{ij}. After working out the derivatives of the different terms, the result is

$$\begin{aligned}\frac{\partial \mathcal{L}}{\partial v_j} &= 2N_{\mathrm{B}} \sum_{l=1}^{t-1} v_l C(l-j) - 2N_{\mathrm{B}} C(j) + 2\left(\sum_\rho \hat{\mathbf{y}}_\rho^\intercal P \hat{\mathbf{y}}_\rho\right) C(j) \\ \frac{\partial \mathcal{L}}{\partial P_{ij}} &= 2\sigma^2 \sum_{l=1}^{N_{\mathrm{S}}} P_{il} \hat{\mathbf{y}}_{\rho,l} \hat{\mathbf{y}}_{\rho,j} + 2\sum_{l=1}^{N_{\mathrm{S}}} P_{il} \langle x_l x_j \rangle - 2\left(\sigma^2 - \sum_{l=1}^{t-1} v_l C(l)\right) \hat{\mathbf{y}}_{\rho,i} \hat{\mathbf{y}}_{\rho,j}\end{aligned}$$

bioRxiv preprint doi: https://doi.org/10.1101/2025.05.26.656161; this version posted May 30, 2025. The copyright holder for this preprint (which was not certified by peer review) is the author/funder, who has granted bioRxiv a license to display the preprint in perpetuity. It is made available under aCC-BY 4.0 International license.

To shorten the notation of terms involving the autocorrelation function $C(s)$, we introduce the vectors $\underline{c} = (C(1), \ldots, C(t-1))$ and $\underline{v} = (v_1, \ldots, v_{t-1})$, and the matrix $\underline{\underline{C}}_{ij} = C(i-j)$. We note that $\underline{\underline{C}}$ is a Toeplitz matrix (*i.e.*, C_{ij} only depends on $i-j$), symmetric since $C(i-j) = C(j-i)$. These properties help to express its inverse explicitly in some cases [74].

Setting the derivatives to zero to find the optimum parameters, we thus have a set of vector and matrix equations for $\underline{v}$ and P, respectively:

$$0 = N_{\mathrm{B}} \underline{\underline{C}} \underline{v} - \left(N_{\mathrm{B}} - \sum_{\rho=1}^{N_{\mathrm{B}}} \hat{\mathbf{y}}_\rho^\intercal P \hat{\mathbf{y}}_\rho \right) \underline{c} \tag{25}$$

$$0 = P \left(\sigma^2 \sum_\rho \hat{\mathbf{y}}_\rho \hat{\mathbf{y}}_\rho^\intercal + \langle \mathbf{x}\mathbf{x}^\intercal \rangle \right) - (\sigma^2 - \underline{v}^\intercal \underline{c}) \sum_{\rho=1}^{N_{\mathrm{B}}} \hat{\mathbf{y}}_\rho \hat{\mathbf{y}}_\rho^\intercal \tag{26}$$

B.2. Solving for P in terms of $\underline{v}$. The best solution path is to first solve for P in terms of $\underline{v}^\intercal \underline{c}$, then solve for $\underline{u}$. We define the $N_{\mathrm{S}} \times N_{\mathrm{S}}$ symmetric matrix

$$M = \sigma^2 \sum_\rho \hat{\mathbf{y}}_\rho \hat{\mathbf{y}}_\rho^\intercal + \langle \mathbf{x}\mathbf{x}^\intercal \rangle \tag{27}$$

which admits a spectral decomposition $M = U \Sigma U^\intercal$ and a Moore-Penrose pseudo-inverse $M^+ = U \Sigma^+ U^\intercal$, which is the actual inverse M^{-1} when M is invertible (*i.e.*, no zero eigenvalue in Σ). Equation 26 thus takes the form $PM = (\sigma^2 - \underline{v}^\intercal \underline{c}) \sum_\rho \hat{\mathbf{y}}_\rho \hat{\mathbf{y}}_\rho^\intercal$, which can be inverted for $P_M = PU\Sigma\Sigma^+ U^\intercal$, the component of P in the subspace spanned by M's eigenvectors of non-zero eigenvalues. The P component in the null space of M, if any, is not constrained by this optimization problem, so we set it to zero, and take $P = P_M$. Hence,

$$P = (\sigma^2 - \underline{v}^\intercal \underline{c}) \sum_\rho \hat{\mathbf{y}}_\rho \hat{\mathbf{y}}_\rho^\intercal M^+ \ . \tag{28}$$

B.3. Solving for $\underline{v}$. We can now insert the implicit solution for P in equation 25 for $\underline{v}$. We first evaluate the term

$$\sum_\rho \hat{\mathbf{y}}_\rho^\intercal P \hat{\mathbf{y}}_\rho = \sum_\rho \hat{\mathbf{y}}_\rho^\intercal \left[(\sigma^2 - \underline{v}^\intercal \underline{c}) \sum_\lambda \hat{\mathbf{y}}_\lambda \hat{\mathbf{y}}_\lambda^\intercal M^+ \right] \hat{\mathbf{y}}_\rho = (\sigma^2 - \underline{v}^\intercal \underline{c}) N_{\mathrm{B}} m_y$$

where we have defined

$$m_y = \frac{1}{N_{\mathrm{B}}} \sum_{\rho,\lambda=1}^{N_{\mathrm{B}}} (\hat{\mathbf{y}}_\rho^\intercal \hat{\mathbf{y}}_\lambda) \hat{\mathbf{y}}_\lambda^\intercal M^+ \hat{\mathbf{y}}_\rho \ . \tag{29}$$

that background odor directions $\hat{\mathbf{y}}_\rho$ were orthogonal, but if that were the case, m_y would simplify to $\frac{1}{N_{\mathrm{B}}} \sum_\rho \hat{\mathbf{y}}_\rho^\intercal M^+ \hat{\mathbf{y}}_\rho$. Inserting in eq. 25, dividing by N_{B}, and isolating $\underline{v}$ by assuming that the autocorrelation matrix $\underline{\underline{C}}$ is invertible, we have

$$\underline{v} = \underline{\underline{C}}^{-1} \underline{c} - \sigma^2 m_y \underline{\underline{C}}^{-1} \underline{c} + m_y (\underline{v}^\intercal \underline{c}) \underline{\underline{C}}^{-1} \underline{c} \ .$$

This is still an implicit expression because $\underline{v}^\intercal \underline{c}$ appears on the right; taking the dot product of this expression with $\underline{c}$, we can isolate $\underline{v}^\intercal \underline{c}$, then reinsert in the implicit equation for $\underline{v}$ to arrive at an explicit solution (*i.e.*, in terms of $\mathcal{P}$, $\mathcal{Q}$ parameters),

$$\underline{v} = \frac{1 - \sigma^2 m_y}{1 - \gamma m_y} \underline{\underline{C}}^{-1} \underline{c} \tag{30}$$

where we have defined

$$\gamma = \underline{c}^\intercal \underline{\underline{C}}^{-1} \underline{c} \ . \tag{31}$$

B.4. Replacing $\underline{v}$ in the P solution. Having solved for $\underline{v}$, we can put it back in 28 to obtain an explicit solution for P,

$$P = \frac{\sigma^2 - \gamma}{1 - \gamma m_y} \sum_{\rho=1}^{N_{\mathrm{B}}} \hat{\mathbf{y}}_\rho \hat{\mathbf{y}}_\rho^\intercal M^+ \ . \tag{32}$$

C. Evaluating the loss function at the optimum. For simplicity, we first rewrite the loss function in eq. 24 using the underlined vector notation for $\underline{c}, \underline{v}, \underline{\underline{C}}$, giving

$$\mathcal{L}_{v,P} = N_{\mathrm{B}} \sigma^2 + N_{\mathrm{B}} \underline{v}^\intercal \underline{\underline{C}} \, \underline{v} + \sigma^2 \sum_\rho \hat{\mathbf{y}}_\rho^\intercal P^\intercal P \hat{\mathbf{y}}_\rho + \langle \mathbf{x}^\intercal P^\intercal P \mathbf{x} \rangle - 2 N_{\mathrm{B}} \underline{v}^\intercal \underline{c} - 2\sigma^2 \sum_\rho \hat{\mathbf{y}}_\rho^\intercal P \hat{\mathbf{y}}_\rho + 2 \underline{v}^\intercal \underline{c} \sum_\rho \hat{\mathbf{y}}_\rho^\intercal P \hat{\mathbf{y}}_\rho \ . \tag{33}$$

bioRxiv preprint doi: https://doi.org/10.1101/2025.05.26.656161; this version posted May 30, 2025. The copyright holder for this preprint (which was not certified by peer review) is the author/funder, who has granted bioRxiv a license to display the preprint in perpetuity. It is made available under aCC-BY 4.0 International license.

We need to evaluate multiple terms to insert the solutions for $\underline{v}$ and P in $\mathcal{L}$. We find several simplifications by using the fact that M and thus M^+ and P are symmetric (eqs. 27 and 32), commuting scalars resulting from intermediate dot products, and renaming indices when appropriate. We find the following terms,

$$\underline{v}^\intercal \underline{\underline{C}}\, \underline{v} = \left(\frac{1-\sigma^2 m_y}{1-\gamma m_y}\right)^2 \underline{c}^\intercal \underline{\underline{C}}^{-1} \underline{\underline{C}}\,\underline{\underline{C}}^{-1} \underline{c} = \left(\frac{1-\sigma^2 m_y}{1-\gamma m_y}\right)^2 \gamma$$

$$\sigma^2 \sum_\rho \hat{\mathbf{y}}_\rho^\intercal P^\intercal P \hat{\mathbf{y}}_\rho = \left(\frac{\sigma^2-\gamma}{1-\gamma m_y}\right)^2 \sum_{\lambda,\mu} \hat{\mathbf{y}}_\mu^\intercal M^+ \left\{\sigma^2 \sum_\rho \hat{\mathbf{y}}_\rho \hat{\mathbf{y}}_\rho^\intercal\right\} M^+ \hat{\mathbf{y}}_\lambda \hat{\mathbf{y}}_\lambda^\intercal \hat{\mathbf{y}}_\mu$$

$$\langle \mathbf{x}^\intercal P^\intercal P \mathbf{x}\rangle = \left(\frac{\sigma^2-\gamma}{1-\gamma m_y}\right)^2 \sum_{\lambda,\mu} \hat{\mathbf{y}}_\mu^\intercal M^+ \left\{\langle \mathbf{x}\mathbf{x}^\intercal\rangle\right\} M^+ \hat{\mathbf{y}}_\lambda \hat{\mathbf{y}}_\lambda^\intercal \hat{\mathbf{y}}_\mu$$

$$\sum_\rho \hat{\mathbf{y}}_\rho^\intercal P \hat{\mathbf{y}}_\rho = \frac{\sigma^2-\gamma}{1-\gamma m_y} \sum_\rho \hat{\mathbf{y}}_\rho^\intercal \sum_\lambda \hat{\mathbf{y}}_\lambda \hat{\mathbf{y}}_\lambda^\intercal M^+ \hat{\mathbf{y}}_\rho = \frac{\sigma^2-\gamma}{1-\gamma m_y} N_\mathrm{B} m_y \ .$$

The second and third terms can be combined by noticing they have the same form with sums over odor indices λ, μ, and combining the bracketed terms to find $\sigma^2 \sum_\rho \hat{\mathbf{y}}_\rho \hat{\mathbf{y}}_\rho^\intercal + \langle \mathbf{x}\mathbf{x}^\intercal\rangle = M$. Then, using the definition of the pseudo-inverse, we have $M^+ M M^+ = M^+$, resulting in

$$\sigma^2 \sum_\rho \hat{\mathbf{y}}_\rho^\intercal P^\intercal P \hat{\mathbf{y}}_\rho + \langle \mathbf{x}^\intercal P^\intercal P \mathbf{x}\rangle = \left(\frac{\sigma^2-\gamma}{1-\gamma m_y}\right)^2 N_\mathrm{B} m_y \ .$$

Combining these expressions in $\mathcal{L}$, we can cancel out a few terms with further algebra and factorize common expressions, finding a surprisingly simple form,

$$\mathcal{L}_{v,P} = N_\mathrm{B} \frac{(\sigma^2-\gamma)(1-\sigma^2 m_y)}{1-\gamma m_y} \ . \tag{34}$$

We notice that the loss seems to scale proportionally with the background subspace dimensions, N_B. Terms σ^2, γ do not depend on N_B but only on the autocorrelation and variance of odor concentrations. The only term that could depend on N_B is $m_y = \frac{1}{N_\mathrm{B}} \sum_{\rho,\lambda=1}^{N_\mathrm{B}} (\hat{\mathbf{y}}_\rho^\intercal \hat{\mathbf{y}}_\lambda) \hat{\mathbf{y}}_\lambda^\intercal M^+ \hat{\mathbf{y}}_\rho$, but we generally expect $N_\mathrm{B} m_y \sim N_\mathrm{B}$. This is especially clear if we assume orthogonality of the $\hat{\mathbf{y}}$, such that $m_y = \frac{1}{N_\mathrm{B}} \sum_{\rho=1}^{N_\mathrm{B}} \hat{\mathbf{y}}_\rho^\intercal M^+ \hat{\mathbf{y}}_\rho \sim \mathcal{O}(1)$.

Hence, there is no obvious tradeoff between the two strategies – predictive filtering and manifold learning – as a function of the background dimension. This makes sense *a posteriori*. Predictive filtering tries to anticipate N_B independent, identically distributed odors, hence the squared errors committed on each background component add up in variance. Meanwhile, the error in manifold learning increase with N_B because a fraction $\sim N_\mathrm{B}$ of the new odor, on average, will lie in the background subspace.

However, there is a tradeoff between the strategies as a function of the autocorrelation time, encoded in the parameter γ, and the dimensionality of the olfactory space, which enters m_y through the new odor statistics $\langle \mathbf{x}\mathbf{x}^\intercal\rangle$ in M. We expect γ to increase with the autocorrelation time scale, and m_y to increase with the olfactory space dimension N_S. Hence, as the autocorrelation time increases, γ diminishes the relative efficacy of manifold learning by reducing the denominator $1-\gamma m_y$, while increasing the importance of predictive filtering by reducing the numerator factor $\sigma^2-\gamma$. This tradeoff will be clearer in the special case studied in section F.

D. Summary of the general optimal solution. We recapitulate the optimization results here. The optimal $\underline{v}$ and P are

$$\underline{v} = \frac{1-\sigma^2 m_y}{1-\gamma m_y} \underline{\underline{C}}^{-1} \underline{c} \tag{30}$$

$$P = \frac{\sigma^2-\gamma}{1-\gamma m_y} \sum_{\rho=1}^{N_\mathrm{B}} \hat{\mathbf{y}}_\rho \hat{\mathbf{y}}_\rho^\intercal M^+ \tag{32}$$

and they give a minimum loss of

$$\mathcal{L}_{v,P} = N_\mathrm{B} \frac{(\sigma^2-\gamma)(1-\sigma^2 m_y)}{1-\gamma m_y} \tag{34}$$

bioRxiv preprint doi: https://doi.org/10.1101/2025.05.26.656161; this version posted May 30, 2025. The copyright holder for this preprint (which was not certified by peer review) is the author/funder, who has granted bioRxiv a license to display the preprint in perpetuity. It is made available under aCC-BY 4.0 International license.

where N_B is the number of i.i.d. background odors, σ^2 is the variance of each odor concentration $\tilde{c}_{\rho,t}$, and where we have defined background parameters

$$\gamma = \underline{c}^\intercal \underline{\underline{C}}^{-1} \underline{c} \tag{31}$$

$$m_y = \frac{1}{N_\mathrm{B}} \sum_{\rho,\lambda=1}^{N_\mathrm{B}} (\hat{\mathbf{y}}_\rho^\intercal \hat{\mathbf{y}}_\lambda) \hat{\mathbf{y}}_\lambda^\intercal M^+ \hat{\mathbf{y}}_\rho \tag{29}$$

$$\text{in which } M = \sigma^2 \sum_\rho \hat{\mathbf{y}}_\rho \hat{\mathbf{y}}_\rho^\intercal + \langle \mathbf{x}\mathbf{x}^\intercal \rangle \tag{27}$$

$$\underline{c}_i = C(i) = \langle \tilde{c}_{\rho,t'} \tilde{c}_{\rho,t' \pm i} \rangle \quad (i \in \{1, 2, \ldots, t-1\})$$
$$\underline{\underline{C}}_{ij} = C(i-j) = \langle \tilde{c}_{\rho,t' \pm i} \tilde{c}_{\rho,t' \pm j} \rangle \tag{35}$$

E. Limiting cases: $P = 0$ and $\underline{v} = 0$.

E.1. Predictive filtering only: $P = 0$. When $P = 0$, we can directly solve eq. 25 for $\underline{v}$, finding

$$\underline{v}_{\,P=0} = \underline{\underline{C}}^{-1} \underline{c}$$

which yields a loss of

$$\mathcal{L}_v = N_\mathrm{B}(\sigma^2 - \gamma) \ . \tag{36}$$

As long as $\gamma < \sigma^2$ – which should be the case if the autocorrelation function decays with time – we have $\mathcal{L}_{v,P} < \mathcal{L}_v$ since $\frac{1-\sigma^2 m_y}{1-\gamma m_y} < 1$ in that case.

E.2. Manifold learning only: $\underline{v} = 0$. When $\underline{v} = 0$, we can directly solve eq. 26 in terms of M^+, resulting in

$$P_{v=0} = \sigma^2 \sum_{\rho=1}^{N_\mathrm{B}} \hat{\mathbf{y}}_\rho \hat{\mathbf{y}}_\rho^\intercal M^+$$

which yields a loss of

$$\mathcal{L}_P = N_\mathrm{B} \sigma^2 (1 - \sigma^2 m_y) \, . \tag{37}$$

As long as $m_y < \frac{1}{\sigma^2}$ – which should be the case since $M^+ \sim 1/\sigma^2$ and m_y is some projection of it on the background subspace – then $\mathcal{L}_{v,P} < \mathcal{L}_P$, since $\frac{1-\gamma/\sigma^2}{1-\gamma m_y} < 1$ in that case.

F. Special case: exponential kernel, $\hat{\mathbf{x}}$ uniform on hypersphere.

To make these results more concrete, we now consider a simple case of background statistics where expressions such as γ, m_y, etc. can be computed analytically in terms of interpretable parameters. We consider an exponential autocorrelation function and new odors uniformly sampled on a hypersphere. This is the case plotted in Figs. 1E and S1.

F.1. Exponential autocorrelation kernel, to evaluate γ. We suppose that each odor concentration $\tilde{c}_{\rho,t'}$ is independent of other odors and forms a Gaussian process with exponential autocorrelation kernel $C(s) = \langle \tilde{c}_{\rho,t'} \tilde{c}_{\rho,t' \pm s} \rangle = \sigma^2 e^{-|s|/\tau}$ with auto-correlation time τ (*i.e.*, the Ornstein-Uhlenbeck process). A small τ corresponds to fast fluctuations compared to the time scale of learning. In this case, the symmetric Toeplitz matrix $\underline{\underline{C}}_{ij} = \sigma^2 (e^{-1/\tau})^{|i-j|}$ is a Kac-Murdock-Szegö matrix (form $A_{ij} = r^{|i-j|}$, $r \neq 1$), which has an explicit inverse, provided in [74, sec. 1.3]. This inverse is the tridiagonal matrix

$$\underline{\underline{C}}^{-1} = \frac{1}{\sigma^2} \frac{1}{1 - e^{-2\tau}} \begin{pmatrix} 1 & -e^{-1/\tau} & 0 & \cdots & 0 \\ -e^{-1/\tau} & 1 + e^{-2/\tau} & -e^{-1/\tau} & \cdots & 0 \\ \vdots & \ddots & \ddots & \ddots & \vdots \\ 0 & \cdots & -e^{-1/\tau} & 1 + e^{-2/\tau} & -e^{-1/\tau} \\ 0 & \cdots & 0 & -e^{-1/\tau} & 1 \end{pmatrix}$$

It is not hard to check that this is indeed the inverse of $\underline{\underline{C}}$. This allows us to evaluate

$$\underline{\underline{C}}^{-1} \underline{c} = \begin{pmatrix} e^{-1/\tau} \\ 0 \\ \vdots \\ 0 \end{pmatrix} \quad \text{thus} \quad \gamma = \underline{c}^\intercal \underline{\underline{C}}^{-1} \underline{c} = \sigma^2 e^{-2/\tau}$$

bioRxiv preprint doi: https://doi.org/10.1101/2025.05.26.656161; this version posted May 30, 2025. The copyright holder for this preprint (which was not certified by peer review) is the author/funder, who has granted bioRxiv a license to display the preprint in perpetuity. It is made available under aCC-BY 4.0 International license.

F.2. New odors $\hat{\mathbf{x}}$ uniform, and $\hat{\mathbf{y}}_\rho$ orthogonal, to evaluate m_y. Moreover, we suppose that new odors take the form $\mathbf{x} = \tilde{c}_x \hat{\mathbf{x}}$, where $\tilde{c}_x$ has variance σ^2_{new} possibly different from the background odors, and where $\hat{\mathbf{x}}$ is uniformly sampled on the N_S-dimensional unit hypersphere. In that case, by symmetry, the new odor covariance matrix is $\langle \mathbf{x}\mathbf{x}^\intercal \rangle = \frac{\sigma^2_{\text{new}}}{N_\text{S}} \mathbb{I}$, with $\mathbb{I}$ the $N_\text{S} \times N_\text{S}$ identity matrix. Additionally, we assume that background odors are orthogonal to each other: $\hat{\mathbf{y}}_\rho^\intercal \hat{\mathbf{y}}_\lambda = \delta_{\rho\lambda}$.

These choices allow us to compute M^+ explicitly. We first note that $M = \langle \mathbf{x}\mathbf{x}^\intercal \rangle + \sigma^2 \sum_\rho \hat{\mathbf{y}}\hat{\mathbf{y}}_\rho$ has full rank, and thus $M^+ = M^{-1}$. We define the rescaled olfactory dimension

$$\tilde{N}_\text{S} = \frac{\sigma^2}{\sigma^2_{\text{new}}} N_\text{S} \tag{38}$$

to simplify expressions (equal to N_S if the background and new odors have the same concentration variance, $\sigma^2_{\text{new}} = \sigma^2$). We can compute that inverse by repeatedly applying the Sherman-Morrison formula to the sequence of matrices $M_k = \frac{1}{\tilde{N}_\text{S}} \mathbb{I} + \sum_{\rho=1}^k \hat{\mathbf{y}}_\rho \hat{\mathbf{y}}_\rho^\intercal$, $k \leq N_\text{B}$. Proceeding by induction, we eventually find the inverse of the full matrix $M^{-1} = \sigma^{-2} M^{-1}_{N_\text{B}}$,

$$M^+ = M^{-1} = \frac{\tilde{N}_\text{S}}{\sigma^2} \left(\mathbb{I} - \frac{\tilde{N}_\text{S}}{\tilde{N}_\text{S} + 1} \sum_{\rho=1}^{N_\text{B}} \hat{\mathbf{y}}_\rho \hat{\mathbf{y}}_\rho^\intercal \right) .$$

We can thus evaluate the matrix product appearing in the optimal P solution,

$$\sum_\lambda \hat{\mathbf{y}}_\lambda \hat{\mathbf{y}}_\lambda^\intercal M^+ = \frac{\tilde{N}_\text{S}}{\sigma^2} \sum_\lambda \hat{\mathbf{y}}_\lambda \hat{\mathbf{y}}_\lambda^\intercal - \frac{\tilde{N}_\text{S}^2}{\sigma^2(\tilde{N}_\text{S} + 1)} \sum_\lambda \hat{\mathbf{y}}_\lambda \hat{\mathbf{y}}_\lambda^\intercal = \frac{\tilde{N}_\text{S}}{\tilde{N}_\text{S} + 1} \frac{1}{\sigma^2} \sum_\rho \hat{\mathbf{y}}_\rho \hat{\mathbf{y}}_\rho^\intercal ,$$

using $\hat{\mathbf{y}}_\lambda^\intercal \hat{\mathbf{y}}_\rho = \delta_{\lambda\rho}$, which also allows us to compute

$$m_y = \frac{1}{N_\text{B}} \sum_{\rho,\mu} \hat{\mathbf{y}}_\rho^\intercal \hat{\mathbf{y}}_\mu \hat{\mathbf{y}}_\mu^\intercal M^+ \hat{\mathbf{y}}_\rho = \frac{\tilde{N}_\text{S}}{\tilde{N}_\text{S} + 1} \frac{1}{\sigma^2} \frac{1}{N_\text{B}} \sum_{\rho,\lambda} \hat{\mathbf{y}}_\rho^\intercal \hat{\mathbf{y}}_\lambda \hat{\mathbf{y}}_\lambda^\intercal \hat{\mathbf{y}}_\rho = \frac{1}{\sigma^2} \frac{\tilde{N}_\text{S}}{\tilde{N}_\text{S} + 1} .$$

F.3. Optimal $\underline{v}, P$ and loss function in this background choice. Inserting the above expressions for γ, M^+, m_y, etc. into the general optimal solution, we find

$$v_1 = \frac{1}{1 + \tilde{N}_\text{S}(1 - e^{-2/\tau})} e^{-1/\tau} \; ; \quad v_{j>1} = 0$$

$$P = \frac{\tilde{N}_\text{S}(1 - e^{-2/\tau})}{1 + \tilde{N}_\text{S}(1 - e^{-2/\tau})} \sum_{\rho=1}^{N_\text{B}} \hat{\mathbf{y}}_\rho \hat{\mathbf{y}}_\rho^\intercal$$

and a minimum loss of

$$\mathcal{L}_{v,P} = N_\text{B} \sigma^2 \frac{1 - e^{-2/\tau}}{1 + \tilde{N}_\text{S}(1 - e^{-2/\tau})} \tag{39}$$

Here, we see clearly that there is no tradeoff as a function of N_B: both strategies have an error that increases proportionally to N_B. At least, we clearly see the transition from predictive filtering to manifold learning as $\tilde{N}_\text{S}$ decreases or τ decreases. For small correlation times, $1 - e^{-2/\tau} \approx 1$, so the main reduction of the loss comes from the $\frac{1}{\tilde{N}_\text{S}+1}$ factor (also the case if $\tilde{N}_\text{S}$ is large). Meanwhile, for large correlation τ, the reduction comes from the numerator $1 - e^{-2/\tau} \approx 0$, while the $\tilde{N}_\text{S}$ term in the denominator is rendered ineffective – the same is true if $\tilde{N}_\text{S}$ is small.

F.4. Special cases $P = 0$ and $\underline{v} = 0$ in this background choice. For further comparison, the optimal solutions and loss function in the pure predictive filtering case ($P = 0$) with our specific background choice are:

$$v_{1,P=0} = e^{-1/\tau} \; ; \quad v_{j\geq 2,P=0} = 0$$

$$\mathcal{L}_v = N_\text{B} \sigma^2 (1 - e^{-2/\tau}) . \tag{40}$$

In the pure manifold learning case ($\underline{v} = 0$), these are rather

$$P_{v=0} = \sigma^2 \sum_{\rho=1}^{N_\text{B}} \hat{\mathbf{y}}_\rho \hat{\mathbf{y}}_\rho^\intercal M^+ = \frac{\tilde{N}_\text{S}}{\tilde{N}_\text{S} + 1} \sum_{\rho=1}^{N_\text{B}} \hat{\mathbf{y}}_\rho \hat{\mathbf{y}}_\rho^\intercal \tag{41}$$

$$\mathcal{L}_P = \frac{N_\text{B} \sigma^2}{\tilde{N}_\text{S} + 1} . \tag{42}$$

bioRxiv preprint doi: https://doi.org/10.1101/2025.05.26.656161; this version posted May 30, 2025. The copyright holder for this preprint (which was not certified by peer review) is the author/funder, who has granted bioRxiv a license to display the preprint in perpetuity. It is made available under aCC-BY 4.0 International license.

F.5. Plots of the loss function versus $\tilde{N}_\mathrm{S}$ and τ for the different strategies. We notice that the loss is proportional to $N_\mathrm{B}\sigma^2$ in all strategies for our special background choice, hence we can illustrate the relative efficacy of predictive filtering and manifold learning by plotting $\mathcal{L}/(N_\mathrm{B}\sigma^2)$ as a function of $\tilde{N}_\mathrm{S}$ and τ, the two background parameters for which there is actually a transition between the two strategies. Fig. S1A-B shows the single-strategy losses $\mathcal{L}_v$ (eq. 40) and $\mathcal{L}_P$ (eq. 42) compared to the loss for both strategies applied simultaneously, $\mathcal{L}_{v,P}$ (eq. 39).

We see that for even a small olfactory space dimension, $\tilde{N}_\mathrm{S} = 50$ as in the fruit fly, manifold learning performs much better than predictive filtering even for moderately long correlation time scales, as discussed in the main text. When $\mathcal{L}_{v,P}$ is close to either $\mathcal{L}_P$ or $\mathcal{L}_v$, it means that the corresponding strategy contributes most of the loss reduction. We can thus draw a "phase" diagram of where habituation is dominated by manifold learning (large $\tilde{N}_\mathrm{S}$, small τ) or by predictive filtering (small $\tilde{N}_\mathrm{S}$, large τ). The (smooth) transition occurs when $\mathcal{L}_P = \mathcal{L}_v$, which happens at $1 - e^{-2/\tau} = \frac{1}{\tilde{N}_\mathrm{S}+1}$, which is at $\tau \approx 2(\tilde{N}_\mathrm{S} + 1)$ for large $\tilde{N}_\mathrm{S}$. This is shown in Fig. 1E. There is a large region (in red) where manifold learning is the most effective strategy.

2. Simulating background odor fluctuations

A. Turbulent statistics. We use the statistics of whiff durations $p_{t_\mathrm{w}}(t_\mathrm{w})$, blank durations $p_{t_\mathrm{b}}(t_\mathrm{b})$, and whiff concentrations $p_c(c)$ derived in [9], with the exponents and statistics for an atmospheric boundary layer. We simulate each background odor concentration as a telegraph-like process, as illustrated in Fig. 1, by drawing a next blank duration at the end of a whiff, a next whiff duration at the end of a blank, and a concentration for each whiff from p_c. As the simulation advances per time steps $\Delta t = 10$ ms, we keep track of how much time is left in the current whiff or blank as well as of the current concentration, and update when that time runs out. This method neglects intra-whiff concentration fluctuations, as well as correlations between successive whiff and blank durations, but captures the main challenges of varying whiff concentrations and power-law (long-tailed) distributions of durations. We now describe these distributions and how we numerically sample from them.

For the durations of whiffs and blanks, the distribution is a power law with exponent $-3/2$ and a lower cutoff at τ_b, τ_w. For numerical stability, we prevent abnormally large durations by imposing also an upper cutoff at $T_\mathrm{max,b}$, $T_\mathrm{max,w}$, and we use sharp cutoffs, corresponding to a probability density function

$$p_{t_\mathrm{x}}(t_\mathrm{x}) = \begin{cases} 0 & \text{if } t_\mathrm{x} < \tau_\mathrm{x} \text{ or } t_\mathrm{x} > T_\mathrm{max,x} \\ \dfrac{1}{A_\mathrm{x}}\left(\dfrac{t_\mathrm{x}}{\tau_\mathrm{x}}\right)^{-3/2} & \text{if } \tau_\mathrm{x} \le t_\mathrm{x} \le T_\mathrm{max,x} \end{cases} \qquad (\mathrm{x} = \mathrm{b \text{ or } w}) \ , \tag{43}$$

where $A_\mathrm{x} = 2\tau_\mathrm{x}\left(1 - (T_\mathrm{max,x}/\tau_\mathrm{x})^{-1/2}\right)$ is a normalization constant. From these distributions, the average duration of a whiff or blank is the geometric average of the limits, since

$$\langle t_\mathrm{x} \rangle = \int_{\tau_\mathrm{x}}^{T_\mathrm{max,x}} \left(\frac{t}{\tau_\mathrm{x}}\right)^{-3/2} \frac{t}{A_\mathrm{x}} \mathrm{d}t = \sqrt{\tau_\mathrm{x} T_\mathrm{max,x}} \quad (\mathrm{x} = \mathrm{b \text{ or } w}) \ ,$$

so the probability χ to be in a whiff is

$$\chi = \frac{\langle t_\mathrm{w} \rangle}{\langle t_\mathrm{w} \rangle + \langle t_\mathrm{b} \rangle} = \left(1 + \sqrt{\frac{\tau_\mathrm{b} T_\mathrm{max,b}}{\tau_\mathrm{w} T_\mathrm{max,w}}}\right)^{-1} .$$

The probability distribution of whiff concentrations c is therefore 0 with probability $1 - \chi$ (illustrated by the point at $c = 0$ in Fig. 1C, left) or, with probability χ, the conditional distribution p_c given there is a whiff. Hence, $p_c(c) = (1 - \chi)\delta(c) + \chi p_c(c|\mathrm{whiff})$. The conditional distribution has a tail $p_c \sim e^{-c/c_0}/c$, where c_0 is a typical concentration scale, and a probability plateau near $c = 0$. As illustrated in Fig. 1C, left, we use a sharp transition at $\alpha_c c_0$ for some $\alpha_c < 1$, with a uniform probability on the range below, $(0, \alpha_c c_0]$, corresponding to a probability density function

$$p_c(c|\mathrm{whiff}) = \begin{cases} \dfrac{1}{A_\alpha} \dfrac{e^{-c/c_0}}{c} & \text{if } c \ge \alpha_c c_0 \\ \dfrac{1}{A_\alpha} \dfrac{e^{-\alpha_c}}{\alpha_c c_0} & \text{if } c < \alpha_c c_0 \end{cases} , \tag{44}$$

where $A_\alpha = e^{-\alpha_c} + E_1(\alpha_c)$ is a normalization constant and E_1 is the exponential integral,

$$E_1(x) = \int_x^\infty \mathrm{d}u \, \frac{e^{-u}}{u} \ .$$

Of note, the average whiff concentration then has the analytical expression

$$\langle c \rangle_\mathrm{whiff} = \int_0^\infty \mathrm{d}c \, c \, p_c(c|\mathrm{whiff}) = \frac{(1 + \alpha_c/2) c_0 e^{-\alpha_c}}{A_\alpha} \ .$$

bioRxiv preprint doi: https://doi.org/10.1101/2025.05.26.656161; this version posted May 30, 2025. The copyright holder for this preprint (which was not certified by peer review) is the author/funder, who has granted bioRxiv a license to display the preprint in perpetuity. It is made available under aCC-BY 4.0 International license.

To sample from these distributions during a simulation, we use the inverse transform method: given a random $\mathrm{uniform}(0,1)$ sample r, we generate a sample of a random variable X following the cumulative distribution function (cdf) F_X as $x = F_X^{-1}(r)$. The cdf for the whiff or blank durations is

$$F_t(t_\mathrm{x}) = \begin{cases} 0 & \text{if } t_\mathrm{x} \leq \tau_\mathrm{x} \\ \dfrac{\left(1 - (t_\mathrm{x}/\tau_\mathrm{x})^{-1/2}\right)}{1 - (T_{\mathrm{max,x}}/\tau_\mathrm{x})^{-1/2}} & \text{if } \tau_\mathrm{x} < t_\mathrm{x} < T_{\mathrm{max,x}} \\ 1 & \text{if } T_{\mathrm{max,x}} \leq t_\mathrm{x} \end{cases} \quad (\mathrm{x} = \mathrm{b \text{ or } w}) \ .$$

Taking the inverse, we generate t_x from uniform samples r as

$$t_\mathrm{x} = \frac{\tau_\mathrm{x}}{\left[1 - r\left(1 - (T_{\mathrm{max,x}}/\tau_\mathrm{x})^{-1/2}\right)\right]^2} \quad (\mathrm{x} = \mathrm{b \text{ or } w}) \ . \tag{45}$$

As a check, notice that $t_\mathrm{x} = \tau_\mathrm{x}$, the lower cutoff, when $r = 0$, and $t_\mathrm{x} = T_{\mathrm{max,x}}$, the upper cutoff, when $r = 1$.

The cdf for the conditional whiff concentrations is

$$F_c(c|\mathrm{whiff}) = \begin{cases} 0 & \text{if } c < 0 \\ \dfrac{c}{\alpha_c A_\alpha c_0 e^{\alpha_c}} & \text{if } 0 \leq c \leq \alpha_c c_0 \\ 1 - \frac{1}{A_\alpha} E_1(c/c_0) & \text{if } c > \alpha_c c_0 \end{cases} \ .$$

Hence, given a random uniform sample r, we generate a sample c as

$$c = F_c^{-1}(r) = \begin{cases} \alpha_c A_\alpha c_0 e^{\alpha_c} r & \text{if } r \leq F_c(\alpha_c c_0) = \frac{1}{e^{\alpha_c} A_\alpha} \\ c_0 E_1^{-1}(A_\alpha(1-r)) & \text{if } \frac{1}{e^{\alpha_c} A_\alpha} < r < 1 \end{cases} \tag{46}$$

where E_1^{-1} is the inverse exponential integral. This inverse function does not have an analytical closed form, so we evaluate $y = E_1^{-1}(x)$ at a given x numerically by solving the equation $E_1(y) - x = 0$ for y, using Brent's method [75] with suitable bounds on the solution. For numerical accuracy, for $x < 1$, we solve in log scale, $\log(E_1(y)) - \log(x) = 0$, to expand the range of $E_1(y)$ values. For larger x, y becomes very small (*e.g.*, $E_1(2) = 0.0489$), so we solve for $z = \log(y)$. For $x > 30$, y is small enough ($y \sim 10^{-13}$) to use the approximation $E_1(y) = -\gamma - \log(y) - \mathcal{O}(y)$ [76, 6.6.1], where $\gamma = 0.577\ldots$ is the Euler-Mascheroni constant, so the equation is inverted directly: $y = e^{-\gamma - x}$.

Hence, overall, for each update when a whiff starts, we use two random $\mathrm{uniform}(0,1)$ samples, one for t_w (using Eq. (45)), one for c (using Eq. (46)); only one sample is needed when a blank starts, for t_w (Eq. (45)). In our simulations, we use the following typical parameter values, the same for all background odors: $\tau_\mathrm{b} = \tau_\mathrm{w} = 10\,\mathrm{ms} = 1$ time step for the lower whiff or blank duration cutoff, $T_{\mathrm{max,w}} = 5000\,\mathrm{ms}$ and $T_{\mathrm{max,b}} = 8000\,\mathrm{ms}$ for the maximum whiff and blank durations respectively, $c_0 = 0.6$ for the (arbitrary) concentration scale, and $\alpha_c = 0.5$ for the lower whiff concentration cutoff.

Moreover, to sample a concentration from the stationary distribution, we draw a first $\mathrm{uniform}(0,1)$ sample r_1 to determine whether there is a whiff, with probability χ (whiff if $r \leq \chi$), or a blank ($c = 0$). Then, if in a whiff, draw a second uniform sample r_2 to generate a concentration c using Eq. (46).

B. Univariate Ornstein-Uhlenbeck process. To simulate a univariate Ornstein-Uhlenbeck (O-U) process $\overline{\nu}(t)$ numerically, we use an exact update rule for finite time steps Δt, derived from the analytical solution of the O-U process. Taking the last time step as a new deterministic initial condition [77, eq. 2.47],

$$\overline{\nu}(t + \Delta t) = \overline{\nu}(t) e^{-\Delta t/\tau_\mathrm{b}} + \sqrt{\sigma^2 \left(1 - e^{-2\Delta t/\tau_\mathrm{b}}\right)}\, \xi(t) \tag{47}$$

where $\xi(t)$ is white noise. The coefficients of $\overline{\nu}(t)$ and $\xi(t)$ can be computed in advance. This rule ensures a steady-state distribution of $\overline{\nu}$ with the desired variance σ^2 even when the simulation time step is on the order of τ_b.

C. Multivariate Ornstein-Uhlenbeck process. The multivariate Langevin equation for the Ornstein-Uhlenbeck process with zero stationary mean, $\overline{\boldsymbol{\nu}}(t)$, is covered in [78, sec. 4.5.6], and can be simulated exactly using the same trick as in the univariate case, Eq. (47). In practice, we used independent and identically distributed background odors in this paper, so the matrices A and B, were diagonal, and the general simulation method effectively reduced to simulating N_B zero-mean univariate processes in parallel (using Eq. (47)), then adding the desired mean vector $\overline{\boldsymbol{\nu}}_0$ to it.

bioRxiv preprint doi: https://doi.org/10.1101/2025.05.26.656161; this version posted May 30, 2025. The copyright holder for this preprint (which was not certified by peer review) is the author/funder, who has granted bioRxiv a license to display the preprint in perpetuity. It is made available under aCC-BY 4.0 International license.

D. Weakly non-Gaussian fluctuating background. We simulate a multivariate Ornstein-Uhlenbeck process, $\mathbf{g}$ as in section 2C, with zero mean and identically distributed variables with stationary variance σ_g^2. Then, we take $c_\gamma = g_0 + g_\gamma + \epsilon g_\gamma^2$, where ϵ should be chosen small and g_0 is the desired zeroths-order mean concentration. Then, the concentrations have the following moments:

$$\begin{aligned}\langle c \rangle &= g_0 + \epsilon\sigma_g^2 \\ \mathrm{Var}\left[c\right] &= \sigma_g^2 + 2\epsilon^2\sigma_g^4 \\ \langle (c - \langle c \rangle)^3 \rangle &= 6\epsilon\sigma_g^4 + 8\epsilon^3\sigma_g^6\end{aligned}$$

These results are straightforward to obtain by expanding c^2 and c^3 and using higher moments of the Gaussian distribution as required, *i.e.*, $\langle g_\gamma^{2k} \rangle = \frac{(2k)!}{2^k k!}\sigma_g^{2k}$. The important outcome is that the third moment is of order ϵ.

E. Log-normal background fluctuations. As above, we simulate identically distributed O-U variables with variance σ_g^2, add a mean g_0 to them, then use them as the $\log_{10}$ of the concentrations, transforming them according to $c_\gamma = 10^{g_\gamma + g_0}$. Then, from the log-normal distribution properties [79], the concentrations themselves have a log-normal distribution with moments

$$\begin{aligned}\langle c \rangle &= 10^{g_0 + \frac{1}{2}\sigma_g^2 \ln 10} \\ \mathrm{Var}\left[c\right] &= \left(10^{\sigma_g^2 \ln 10} - 1\right) 10^{2g_0 + \sigma_g^2 \ln 10} \\ \langle (c - \langle c \rangle)^3 \rangle &= \mathrm{Var}\left[c\right]^{3/2} \left(10^{\sigma_g^2 \ln 10} + 2\right) \sqrt{10^{\sigma_g^2 \ln 10} - 1}\ .\end{aligned}$$

We test habituation to this background with IBCM and BioPCA networks in Fig. S6.

F. Numerical stability. Numerical integration of the W equations displays instabilities when $\bar{\mathbf{h}}$ reaches large magnitudes, *e.g.*, when increasing the scale parameter Λ (Fig. S11 and section 8). To ensure these are numerical errors rather than a true dynamical instability of the fixed points, we perform a nonlinear numerical stability analysis of the Euler integrator. This integrator, applied to the W matrix equation, is effectively a discrete mapping,

$$\begin{aligned}W_{t+1} &= W_t + \Delta t(\alpha \mathbf{y}\bar{\mathbf{h}}^\intercal - \beta W_t) = W_t + \Delta t(\alpha \mathbf{s}\bar{\mathbf{h}}^\intercal - \alpha W_t \overline{\mathbf{h}\mathbf{h}}^\intercal - \beta W_t) \\ &= B\Delta t + W_t(\mathbb{I} + \Delta t J) \quad \text{where } B = \alpha \mathbf{s}\bar{\mathbf{h}}^\intercal\ ,\ J = -\alpha\overline{\mathbf{h}\mathbf{h}}^\intercal - \beta\mathbb{I}\end{aligned}$$

We consider the worst-case scenario, when $\bar{\mathbf{h}}$ reaches its maximal magnitude encountered in a simulation, $\bar{\mathbf{h}} = \mathbf{h}_{\max}$ and $J_{\max} = -\alpha\mathbf{h}_{\max}\mathbf{h}_{\max}^\intercal - \beta\mathbb{I}$, and iterate the map in this case, which gives

$$W_{t+N} = \delta t B \sum_{n=0}^{N-1} (\mathbb{I} + \Delta t J_{\max})^n + W_t(\mathbb{I} + \Delta t J_{\max})^N$$

Consequently, the stability depends on the eigenvalues of $A = \mathbb{I} + \Delta t J_{\max}$, but not on $\Delta t B$ (because the latter is not raised to some power). Indeed, first note that the matrix A is symmetric and diagonalizable as $A = UDU^\dagger$ with $D = \mathrm{diag}(\lambda_i)$. Then, if A has at least one eigenvalue with magnitude $|\lambda_j| > 1$, then $A^N = U\mathrm{diag}(\lambda_i^N)U^\dagger$ will have a diverging component as the mapping is iterated (as N increases). To find the threshold where this happens, we can read out the eigenvalues from the expression of $A = (1 - \beta\Delta t)\mathbb{I} - \alpha\Delta t\|\mathbf{h}_{\max}\|^2\hat{\mathbf{h}}_{\max}\hat{\mathbf{h}}_{\max}^\intercal$,

$$\lambda_i = \begin{cases} 1 - \beta\Delta t - \|\mathbf{h}_{\max}\|^2\alpha\Delta t & \text{once} \\ 1 - \beta\Delta t & N_\mathrm{I} - 1\,\text{times}\end{cases}$$

To see this, consider the rotation matrix R that aligns $\mathbf{h}_{\max}$ with one of the unit vectors, *e.g.*, $(1, 0, \ldots 0)$: $RAR^\intercal$ is then diagonal with $\lambda_1 = 1 - \beta\Delta t - \|\mathbf{h}_{\max}\|^2\alpha\Delta t$ in one row, $\lambda_2 = 1 - \beta\Delta t$ on the others. For large LN activity, λ_1 is first to reach a magnitude > 1, by becoming negative. Hence, we can predict that numerical instabilities arise in the Euler integrator when

$$\lambda_1 < -1 \Rightarrow \|\mathbf{h}_{\max}\|^2 > \frac{2 - \beta\Delta t}{\alpha\Delta t} \quad \text{or} \quad \Delta t > \frac{2}{\beta + \alpha\|\mathbf{h}_{\max}\|^2}\ . \tag{48}$$

Given a simulation of the M weights and $\bar{\mathbf{h}}$ (which are not destabilized), we can thus anticipate whether the W integration should be unstable by extracting $\|h_{\max}\|$ from the simulation. The vertical lines in Fig. S11 indicate the smallest Λ value for which this threshold is reached, in each model, in the turbulent background considered. They coincide well with the observed drops in model performance, confirming these are due to Euler integrator instabilities. A linear stability analysis of the W equations (computing the Jacobian of the ODE near the fixed point, etc.) confirms that the W is linearly stable for any Λ. Hence, the observed divergences are numerical limitations rather than true model performance drops and would be remedied by decreasing the time step, Δt.

bioRxiv preprint doi: https://doi.org/10.1101/2025.05.26.656161; this version posted May 30, 2025. The copyright holder for this preprint (which was not certified by peer review) is the author/funder, who has granted bioRxiv a license to display the preprint in perpetuity. It is made available under aCC-BY 4.0 International license.

3. Average subtraction model

In this short section, we examine the average subtraction model from [36], and explain how it is insufficient against fluctuating backgrounds. In our notation, it corresponds to a vector $\mathbf{w}$ of inhibitory weights learned as

$$\frac{d\mathbf{w}}{dt} = \alpha\mathbf{s}(t) - (\alpha+\beta)\mathbf{w}(t) \tag{49}$$

and a PN response $\mathbf{y}(t) = \mathbf{s}(t) - \mathbf{w}$ (the LN activity is fixed to 1). If this network is exposed to a constant background odor $\mathbf{s}_{\mathrm{b},0}$, the inhibition vector $\mathbf{w}$ converges to $\mathbf{w}_{\mathrm{ss}} = \frac{\alpha}{\alpha+\beta}\mathbf{s}_{\mathrm{b},0}$, so the background is then perfectly subtracted. However, this strategy fails if the background vector $\mathbf{s}_{\mathrm{b}}(t)$ fluctuates randomly over time. In this case, equation 49 amounts to computing the average background over a time window of duration $\frac{1}{\alpha+\beta}$, as seen from the formal solution of the stochastic equation,

$$\mathbf{w}(t) = \alpha \int_0^t dt' e^{-(\alpha+\beta)(t-t')}\mathbf{s}_{\mathrm{b}}(t') \ ,$$

assuming $\mathbf{s}_{\mathrm{b}}(t)$ started at a given initial value $\mathbf{s}_{\mathrm{b},0}$. From the formal solution, assuming the background is a stationary process, the steady-state average value of $\mathbf{w}$ is therefore proportional to the average background, $\langle\mathbf{w}\rangle = \frac{\alpha}{\alpha+\beta}\langle\mathbf{s}_{\mathrm{b}}\rangle$. If the background process has an autocorrelation time scale much faster than the learning rate, $\tau_{\mathrm{b}} \ll \frac{1}{\alpha}$, with its elements approximately obeying $\langle\Delta s_i(t_1)\Delta s_i(t_2)\rangle = \sigma_{s_i}^2 e^{-|t_2-t_1|/\tau_{\mathrm{b}}}$, then to leading order, the inhibitory weights have a small variance

$$\mathrm{Var}\left[w_i\right] = \alpha\tau_{\mathrm{b}}\,\frac{\alpha}{\alpha+\beta}\sigma_{s_i}^2 \ .$$

Hence, the inhibitory weights do not fluctuate much around the constant average background, $\langle s_{\mathrm{b}}\rangle$, since the factor $\alpha\tau_{\mathrm{b}} \ll 1$. Therefore, this model computes the average background, scaled by $\frac{\alpha}{\alpha+\beta}$, and subtracts this fixed quantity from $\mathbf{s}_{\mathrm{b}}(t)$ to obtain the projection neuron activity $\mathbf{y}(t) = \mathbf{s}_{\mathrm{b}}(t) - \mathbf{w}$. Consequently, the variance of the PN activity, $\mathbf{y}(t)$, is not reduced compared to the variance of the background:

$$\begin{aligned}\langle\mathbf{y}\rangle = \langle\mathbf{s}_{\mathrm{b}}\rangle - \langle\mathbf{w}\rangle = \frac{\beta}{\alpha+\beta}\langle\mathbf{s}_{\mathrm{b}}\rangle \ \ &\text{(reduction of the average)}\\ \text{but} \ \ \mathrm{Var}\left[s_i\right] \sim \sigma_{s_i}^2 \ \ \forall i \quad &\text{(no reduction of the fluctuations)} \ .\end{aligned} \tag{50}$$

These large remaining fluctuations in PN activity mix with new odors appearing in the landscape and thus hinder their recognition; this effect explains why the average subtraction model does not provide a significant reduction in PN response to the background, or improvement in new odor recognition compared to the absence of habituation in Figures 2, 5, S2, and others.

4. Analytical solution of the IBCM model's average fixed points

To understand what projections are learned by IBCM neurons, we derive analytical expressions for the synaptic weights $\mathbf{m}$ of an IBCM neuron at stationary state. We first establish approximate fixed point equations for these weights averaged over fast fluctuations of the background process $\mathbf{s}_{\mathrm{b}}(t)$, assuming perfect separation of time scales between $\mathbf{s}$, Θ, and $\mathbf{m}$. Then, we obtain exact solutions to these approximate equations.

A. Establishing the IBCM fixed point equations. Let's first recall the stochastic differential equations describing the synaptic weights learning of an IBCM neuron in a network with feedforward lateral coupling (Methods). For tractability, we assume the activation function ϕ is the identity function (instead of a nonlinearity like $\tanh$), such that $\overline{\mathbf{h}} = LM\mathbf{s}$. We also set the decay term $-\varepsilon\mu\mathbf{m}_i$ to zero. Then, for each neuron i,

$$\frac{\mathrm{d}\mathbf{m}_i}{\mathrm{d}t} = \mu_{\overline{\Theta}_i}\overline{h}_i\left(\overline{h}_i - \overline{\Theta}_i\right)\mathbf{s}(t) - \eta\sum_{j\neq i}\mu_{\overline{\Theta}_j}\overline{h}_j\left(\overline{h}_j - \overline{\Theta}_j\right)\mathbf{s}(t) \tag{51}$$

$$\frac{\mathrm{d}\overline{\Theta}_i}{\mathrm{d}t} = \frac{1}{\tau_\Theta}((\overline{h}_i)^2 - \overline{\Theta}_i) \ . \tag{52}$$

The reduced activity of interneuron i is $\overline{h}_i = \overline{\mathbf{m}}_i \cdot \mathbf{s}(t)$, where the reduced weights $\overline{\mathbf{m}}_i = \mathbf{m}_i - \eta\sum_{j\neq i}\mathbf{m}_j$. Hence, $\overline{h}_i$ varies in time on two separate scales: rapidly with sensory inputs $\mathbf{s}(t)$ fluctuating on time scale τ_{b}, and gradually as the synaptic weights $\overline{\mathbf{m}}_i$ are learned.

To make analytical progress, we focus on averages over fast background fluctuations, denoted by brackets $\langle\cdot\rangle$, while the slow dynamical variables (M, $\overline{\Theta}$) remain unchanged. Moreover, we make a quasi-static approximation on the thresholds: we assume τ_Θ is slow enough to average over background fluctuations, yet fast enough that $\overline{m}_i$ remains unchanged over the averaging time

bioRxiv preprint doi: https://doi.org/10.1101/2025.05.26.656161; this version posted May 30, 2025. The copyright holder for this preprint (which was not certified by peer review) is the author/funder, who has granted bioRxiv a license to display the preprint in perpetuity. It is made available under aCC-BY 4.0 International license.

window (*i.e.*, we neglect correlations between $\overline{m}$ and $\overline{\Theta}$). Hence, as in the original IBCM models [43], we assume a perfect separation between the background, threshold, and synaptic weight time scales: $\tau_{\mathrm{b}} \ll \tau_{\Theta} \ll \frac{1}{\mu}$.

Thus, averaging equation 52, and setting $\frac{\mathrm{d}\langle\overline{\Theta}_i\rangle}{\mathrm{d}t} = 0$, we find

$$\langle\overline{\Theta}_i\rangle = \langle\overline{h}_i^2\rangle$$

and we replace $\overline{\Theta}_i$ in the $\mathbf{m}$ equation 51 with this average. Averaging that equation as well, and setting $\frac{\mathrm{d}\langle\overline{\mathbf{m}}_i\rangle}{\mathrm{d}t} = 0$, we find the IBCM fixed point equations:

$$0 = \mu_{\langle\overline{\Theta}_i\rangle} \left\langle \overline{h}_i \left(\overline{h}_i - \langle\overline{\Theta}_i\rangle\right) \mathbf{s}(t)\right\rangle - \eta \sum_{j\neq i} \mu_{\langle\overline{\Theta}_j\rangle} \left\langle \overline{h}_j \left(\overline{h}_j - \langle\overline{\Theta}_j\rangle\right) \mathbf{s}(t)\right\rangle \quad i \in \{1, 2, \ldots, N_{\mathrm{I}}\} \;. \tag{53}$$

Defining terms $\varphi_i = \mu_{\langle\overline{\Theta}_i\rangle} \left\langle \overline{h}_i \left(\overline{h}_i - \langle\overline{\Theta}_i\rangle\right) \mathbf{s}(t)\right\rangle$ and combining them in a N_{I}-dimensional vector φ, this system of equations can be written in matrix form

$$0 = L\varphi$$

where L is the $N_{\mathrm{I}} \times N_{\mathrm{I}}$ matrix of feedforward inhibitory coupling between interneurons, with 1 on the diagonal and $-\eta$ everywhere else. This L is a circulant matrix (each row is a cyclic permutation of the previous row by one element to the right); hence, except in the pathological cases $\eta = -1$ or $\eta = \frac{1}{N_{\mathrm{I}}-1}$ (for which some of its eigenvalues are zero), L is invertible and the unique solution is $\varphi = L^{-1}0 = 0$. Therefore, in general, the fixed points of the IBCM network are found by setting each φ_i term to zero individually

$$0 = \mu_{\langle\overline{\Theta}_i\rangle} \left\langle \overline{h}_i \left(\overline{h}_i - \langle\overline{\Theta}_i\rangle\right) \mathbf{s}(t)\right\rangle \;\; \forall i \in \{1, 2, \ldots, N_{\mathrm{I}}\} \;. \tag{54}$$

Hence, in terms of the reduced synaptic weights $\overline{\mathbf{m}}_i$ and activities $\overline{h}_i$, the fixed point equations for the network of IBCM neurons decouple to take the same form as that of a single IBCM neuron.

Before proceeding to solve this set of equations, we note that the actual synaptic weights $\mathbf{m}_i$ can be found from the $\overline{\mathbf{m}}_i$ solutions by inverting the matrix equation

$$\overline{M} = LM \Rightarrow M = L^{-1}\overline{M}$$

where $\overline{M}$ contains the reduced $\overline{\mathbf{m}}_i$ in its rows. From the eigenvectors and eigenvalues of the circulant matrix L, the inverse matrix elements are

$$L^{-1}_{ij} = \begin{cases} \frac{(N_{\mathrm{I}}-2)\eta-1}{(N_{\mathrm{I}}-1)\eta^2+(N_{\mathrm{I}}-2)\eta-1} & \text{on the diagonal} \\ \frac{-\eta}{(N_{\mathrm{I}}-1)\eta^2+(N_{\mathrm{I}}-2)\eta-1} & \text{off-diagonal} \end{cases}$$

so the synaptic weights $\mathbf{m}_i$ can be recovered from the reduced ones, if necessary, as

$$\mathbf{m}_i = \frac{[(N_{\mathrm{I}}-2)\eta - 1]\overline{\mathbf{m}}_i - \eta \sum_{k\neq j} \overline{\mathbf{m}}_k}{(N_{\mathrm{I}}-1)\eta^2 + (N_{\mathrm{I}}-2)\eta - 1} \quad \text{if } \eta \neq \frac{1}{N_{\mathrm{I}}-1} \text{ or } -1 \;. \tag{55}$$

B. IBCM fixed point equations for i.i.d. background concentrations. We will express the solutions to the fixed point equations in terms of the alignments, or dot products, of the IBCM synaptic weights with the background odors,

$$\overline{h}_{i\gamma} = \overline{\mathbf{m}}_i \cdot \hat{\mathbf{s}}_\gamma \;. \tag{56}$$

and we imply these are averaged over fast background fluctuations (*i.e.*, we really look at $\langle\overline{\mathbf{m}}_i\rangle \cdot \hat{\mathbf{s}}_\gamma$), The odor vectors $\hat{\mathbf{s}}_\gamma$ do not have to be orthogonal, but we assume they form a linearly independent set. Specifying these dot products is sufficient to obtain the complete solution, since the IBCM dynamics only update weights in the background subspace of $\mathbf{s}(t)$ (eq. 51). The other directions are reduced to zero by the slow decay term, $-\delta\mu\mathbf{m}_i$, that we have added to the full dynamics (Methods). For the rest of this section, we work with reduced variables and drop the overlines on $\overline{\Theta}_i$, $\overline{\mathbf{m}}_i$, and $\overline{h}_{i\gamma}$ to simplify notation. We also drop the IBCM neuron index i since the fixed point equations eq. 54 are identical and solved independently for each neuron.

Since we have decoupled the Θ and $\mathbf{m}$ fluctuations in Eq. (53), we can divide by the learning rate and write

$$0 = \langle h(t)^2 \mathbf{s}(t)\rangle - \langle\Theta\rangle \langle h\mathbf{s}(t)\rangle$$

We replace $\langle\Theta\rangle = \langle h^2\rangle$, using our quasi-static approximation. We now assume that the odor concentrations $h_\gamma(t)$ are independent, identically distributed stationary processes. We write $h_\gamma(t) = \langle c\rangle + \tilde{c}_\gamma(t)$ where $\langle\tilde{c}_\gamma\rangle = 0$. The concentrations have mean $\langle c\rangle$, variance $\langle\tilde{c}_\gamma^2\rangle = \sigma^2$, and third moment $\langle\tilde{c}^3\rangle = m_3$. We also let $\mathbf{s}(t) = \mathbf{s}_{\mathrm{d}} + \sum_{\gamma=1}^{N_{\mathrm{B}}} c_\gamma(t)\hat{\mathbf{s}}_\gamma$ and $h(t) = h_{\mathrm{d}} + \sum_\gamma h_\gamma\tilde{c}_\gamma(t)$,

bioRxiv preprint doi: https://doi.org/10.1101/2025.05.26.656161; this version posted May 30, 2025. The copyright holder for this preprint (which was not certified by peer review) is the author/funder, who has granted bioRxiv a license to display the preprint in perpetuity. It is made available under aCC-BY 4.0 International license.

where we have defined $\mathbf{s}_\mathrm{d} = \langle c \rangle \sum_\gamma \hat{\mathbf{s}}_\gamma$ and $h_\mathrm{d} = \mathbf{m} \cdot \mathbf{s}_\mathrm{d}$. We proceed to compute the averages appearing in the fixed point equation:

$$\begin{aligned}
\langle h(t)^2 \rangle &= \langle (\mathbf{m} \cdot \mathbf{s})^2 \rangle = \sum_{\gamma,\rho} \langle c_\gamma(t) c_\rho(t) \rangle h_\gamma h_\rho = h_\mathrm{d}^2 + \sigma^2 u^2 \\
\langle h(t) \mathbf{s}(t) \rangle &= h_\mathrm{d} \langle c \rangle \sum_\gamma \hat{\mathbf{s}}_\gamma + \sum_{\gamma,\rho} \langle \tilde{c}_\gamma \tilde{c}_\rho \rangle h_\rho \hat{\mathbf{s}}_\gamma = \sum_\gamma (\langle c \rangle h_\mathrm{d} + \sigma^2 h_\gamma) \hat{\mathbf{s}}_\gamma \\
\langle h(t)^2 \mathbf{s}(t) \rangle &= h_\mathrm{d}^2 \mathbf{s}_\mathrm{d} + 2 h_\mathrm{d} \sum_{\rho\gamma} \langle \tilde{c}_\rho \tilde{c}_\gamma \rangle h_\rho \hat{\mathbf{s}}_\gamma + \mathbf{s}_\mathrm{d} \sum_{\rho,\gamma} h_\rho h_\gamma \langle \tilde{c}_\rho \tilde{c}_\gamma \rangle + \sum_{\rho,\lambda,\gamma} \langle \tilde{c}_\rho \tilde{c}_\lambda \tilde{c}_\gamma \rangle h_\rho h_\lambda \hat{\mathbf{s}}_\gamma \\
&= h_\mathrm{d}^2 \mathbf{s}_\mathrm{d} + 2 h_\mathrm{d} \sigma^2 \sum_\gamma h_\gamma \hat{\mathbf{s}}_\gamma + \sigma^2 u^2 \mathbf{s}_\mathrm{d} + m_3 \sum_\gamma h_\gamma^2 \hat{\mathbf{s}}_\gamma
\end{aligned}$$

where we have defined

$$h_\mathrm{d} = \mathbf{m} \cdot \mathbf{s}_\mathrm{d} = \langle c \rangle \sum_\gamma h_\gamma \quad \text{and} \quad u^2 = \sum_{\gamma=1}^{N_\mathrm{B}} h_\gamma^2 \tag{57}$$

Combining and expanding $\mathbf{s}_\mathrm{d} = \langle c \rangle \sum_\gamma \hat{\mathbf{s}}_\gamma$, we have

$$0 = \sum_{\gamma=1}^{N_\mathrm{B}} \left[\langle c \rangle (h_\mathrm{d}^2 + \sigma^2 u^2)(1 - h_\mathrm{d}) - \sigma^2 h_\gamma (h_\mathrm{d}^2 + \sigma^2 u^2 - 2 h_\mathrm{d}) + m_3 h_\gamma^2 \right] \hat{\mathbf{s}}_\gamma$$

Since the $\hat{\mathbf{s}}_\gamma$ odors are linearly independent, we have, for each IBCM neuron, a set of N_B equations specifying the neuron's alignment with each odor, h_γ. These are the fixed point equations to solve for the h_γs:

$$0 = \langle c \rangle (h_\mathrm{d}^2 + \sigma^2 u^2)(1 - h_\mathrm{d}) - \sigma^2 h_\gamma (h_\mathrm{d}^2 + \sigma^2 u^2 - 2 h_\mathrm{d}) + m_3 h_\gamma^2 \qquad \forall \gamma \in \{1, 2, \ldots, N_\mathrm{B}\}, \forall \, \text{neuron} \tag{58}$$

These equations are cubic polynomials in the h_γs, since $h_\mathrm{d} = \langle c \rangle \sum_\gamma h_\gamma$ and $u^2 = \sum_\gamma h_\gamma^2$. There is always a fixed point at $h_\gamma = 0 \, \forall \gamma$, but it is unstable; trajectories initialized near the origin move away from it during habituation.

C. Solution for a zero third moment background processes. We first examine solutions when $m_3 = 0$, *e.g.*, Gaussian backgrounds, since the solutions simplify greatly in that case. The equations then have two terms,

$$0 = \langle c \rangle (h_\mathrm{d}^2 + \sigma^2 u^2)(1 - h_\mathrm{d}) - \sigma^2 h_\gamma (h_\mathrm{d}^2 + \sigma^2 u^2 - 2 h_\mathrm{d})$$

which can be made individually zero by setting $h_\mathrm{d} = 1$ and $\sigma^2 u^2 = 1$. Thus, these fixed points are defined by two constraints,

$$\sum_\gamma \bar{h}_\gamma = \frac{1}{\langle c \rangle} \tag{59}$$

$$\sum_\gamma \bar{h}_\gamma^2 = \frac{1}{\sigma^2} \tag{60}$$

which correspond, geometrically, to the intersection of a hyperplane where coordinates sum to one, and a hypersphere of radius $1/\sigma^2$, respectively. All $\mathbf{m}$ weights on the resulting $(N_\mathrm{B} - 2)$-dimensional surface in the background subspace are non-isolated fixed points when $N_\mathrm{B} > 2$; Fig. S5A shows a three-dimensional background example, with each neuron converging to a point on the ring defined by these equations. For a two-dimensional background, there can be zero, one, or two isolated fixed points, while for a one-dimensional background, these equations do not apply.

The fixed point equation for a Gaussian background admits another set of solutions, where the two terms are not individually zero. Isolating h_γ, we find it must have the same value for every odor, $h_\gamma = h_0$. Then, $h_\mathrm{d} = N_\mathrm{B} h_0$, $u^2 = N_\mathrm{B} h_0^2$, and we can solve to find

$$h_\gamma = h_0 = \frac{\langle c \rangle + 2\sigma^2/(N_\mathrm{B} + \sigma^2)}{\sigma^2 + N_\mathrm{B} \langle c \rangle} \quad \forall \gamma \in \{1, 2, \ldots, N_\mathrm{B}\} \,.$$

For a one-odor background, this solution would be the stable fixed point; for higher dimensions, we find in practice that it is unstable (see also D.1).

bioRxiv preprint doi: https://doi.org/10.1101/2025.05.26.656161; this version posted May 30, 2025. The copyright holder for this preprint (which was not certified by peer review) is the author/funder, who has granted bioRxiv a license to display the preprint in perpetuity. It is made available under aCC-BY 4.0 International license.

D. Solution for a general background with non-zero third moment. We return to solving the full fixed point equations, eq. 58, when $m_3 \neq 0$. Now, because of the $m_3 h_\gamma$ term, setting $h_{\mathrm{d}} = 1$ and $u^2 = 1/\sigma^2$ does not satisfy the equations, so we must solve for individual h_γs. This leads to a finite number of isolated fixed points: as shown in Fig. S5B, the third moment of the background breaks the degeneracy seen in the Gaussian case.

First, we notice that the dot products $\overline{h}_\gamma$ of an IBCM neuron can only take at most two different values at steady-state. To show this, we take the difference between the equation for h_γ and some other h_α, which gives

$$0 = m_3(h_\gamma + h_\alpha)(h_\gamma - h_\alpha) - (h_{\mathrm{d}}^2\sigma^2 + \sigma^4 u^2 - 2h_{\mathrm{d}}\sigma^2)(h_\gamma - h_\alpha) \ .$$

Either $h_\gamma = h_\alpha$, or if they have different values, then they are related by the constraint

$$h_\gamma + h_\alpha = \frac{h_{\mathrm{d}}^2\sigma^2 + \sigma^4 u^2 - 2h_{\mathrm{d}}\sigma^2}{m_3} \ . \qquad \textbf{(61)}$$

Note that constraint 61 is removed for Gaussian backgrounds $m_3 = 0$, explaining the non-isolated fixed points in that special case. For any pair γ, α, the r.h.s. is the same; if we imagine a third dot product h_β, then $h_\gamma + h_\alpha = h_\gamma + h_\beta \Rightarrow h_\alpha = h_\beta$, implying that there cannot be a third distinct value. Therefore, either all h_γs are equal, or they each take one of two possible values.

D.1. All h_γs are equal. First, consider the case where all $h_\gamma = y$, a unique dot product value. Then $u^2 = N_{\mathrm{B}} y^2$ and $h_{\mathrm{d}} = N_{\mathrm{B}} \langle c \rangle y$. Inserting in Eq. (58), we can factor out y^2, giving

$$0 = y^2 \left[N_{\mathrm{B}}^2 \langle c \rangle^3 + 3\sigma^2 N_{\mathrm{B}} \langle c \rangle + m_3 - y(N_{\mathrm{B}}^3 \langle c \rangle^4 + 2\sigma^2 N_{\mathrm{B}}^2 \langle c \rangle^2 + \sigma^4 N_{\mathrm{B}}) \right] \ .$$

So, either $y = 0$, which is an unstable fixed point, or

$$y = \frac{N_{\mathrm{B}}^2 \langle c \rangle^3 + 3\sigma^2 N_{\mathrm{B}} \langle c \rangle + m_3}{N_{\mathrm{B}}^3 \langle c \rangle^4 + 2\sigma^2 N_{\mathrm{B}}^2 \langle c \rangle^2 + \sigma^4 N_{\mathrm{B}}} \ . \qquad \textbf{(62)}$$

We conjecture that this fixed point is always unstable, based on the linear stability analysis of section 4F below. Fig. S3 shows that in the background process examples we considered, it is a saddle point, approached before the IBCM neuron becomes selective for a background odor.

D.2. Two different h_γ values (general case. Second, consider the case where $\mathbf{m}$ has a dot product equal to y_1 with k_1 odors, and equal to y_2 with the remaining $k_2 = N_{\mathrm{B}} - k_1$ odors. We let $y_1 > y_2$ by convention. The values y_1 and y_2 will depend on the repartition k_1, k_2, but there will be a unique pair y_1, y_2 for each choice of k_1, k_2. Moreover, in this case,

$$h_{\mathrm{d}} = \langle c \rangle (k_1 y_1 + k_2 y_2) \qquad \textbf{(63)}$$

$$u^2 = k_1 y_1^2 + k_2 y_2^2 \ . \qquad \textbf{(64)}$$

Then, the set of N_{B} equations 58 really reduces to two equations, one for all the $h_\gamma = y_1$ and the other for y_2, which are symmetric under $1 \leftrightarrow 2$.

We start from Eq. (61) – the difference between the two values of h_γ. We rewrite the equation in terms of y_1 and y_2 as

$$0 = h_{\mathrm{d}}^2 - 2h_{\mathrm{d}} + \sigma^2 u^2 - \frac{m_3}{\sigma^2}(y_1 + y_2) \qquad \textbf{(65)}$$

by letting one of the h_γs be equal to y_1 and the other, to y_2 . We use this equation to replace, where appropriate, the following term in the fixed point equation 58,

$$h_{\mathrm{d}}^2 + \sigma^2 u^2 = 2h_{\mathrm{d}} + \frac{m_3}{\sigma^2}(y_1 + y_2)$$

for, say, $h_\gamma = y_1$ (using y_2 would not make a difference), resulting in

$$0 = \sigma^2 u^2 - h_{\mathrm{d}}^2 - \frac{m_3}{\sigma^2} h_{\mathrm{d}}(y_1 + y_2) - \frac{m_3}{\langle c \rangle} y_1 y_2 \qquad \textbf{(66)}$$

Together, equations 65 and 66 form our system of equations to solve for y_1 and y_2. Obtaining the latter was the crucial simplification to make, because it eliminates terms linear or cubic in y_i, allowing us to easily isolate y_2 in terms of y_1 (or vice-versa)[1]. Indeed, writing h_{d} and u^2 in terms of the y_i (equations 63-64), we find it takes the simple, symmetric form

$$0 = a_1 y_1^2 - b y_1 y_2 + a_2 y_2^2$$

[1] We have managed to reduce the degree from cubic to quadratic because the substitutions eliminated one root $y_1 = y_2 = 0$, which was not interesting.

bioRxiv preprint doi: https://doi.org/10.1101/2025.05.26.656161; this version posted May 30, 2025. The copyright holder for this preprint (which was not certified by peer review) is the author/funder, who has granted bioRxiv a license to display the preprint in perpetuity. It is made available under aCC-BY 4.0 International license.

where

$$a_i = \sigma^2 k_i - \langle c \rangle^2 k_i^2 - \frac{m_3 \langle c \rangle}{\sigma^2} k_i \quad (i \in \{1, 2\})$$
$$b = 2 \langle c \rangle^2 k_1 k_2 + \frac{m_3 \langle c \rangle}{\sigma^2}(k_1 + k_2) + \frac{m_3}{\langle c \rangle} \ . \tag{67}$$

This equation makes clear the symmetry of solutions under exchange of labels $1 \leftrightarrow 2$, confirming that we can keep only the roots where $y_1 > y_2$, knowing that other roots would be found by exchanging k_1 and k_2; in other words, the roots y_1', y_2' for $k_1' = k_2$, $k_2' = k_1$, are the solutions for k_1, k_2 with $y_2 > y_1$ (this can be checked explicitly with the solution below). For now, we write y_2 in terms of y_1,

$$y_2 = \left(\frac{b \pm \sqrt{b^2 - 4a_1 a_2}}{2a_2} \right) y_1 = \alpha_\pm y_1 \ . \tag{68}$$

Formally, the numerator should be $by_1 \pm \sqrt{b^2 - 4a_1 a_2}|y_1|$, but we can absorb the absolute value into $\pm$, compute the solution for both α values, and keep the one with $y_1 > y_2$ at the end. We now insert $y_2 = \alpha y_1$ into Eq. (65); another root $y_1 = y_2 = 0$ can be factored out, and we find the non-trivial solution

$$y_1 = \frac{2 \langle c \rangle (k_1 + \alpha k_2) + \frac{m_3}{\sigma^2}(1 + \alpha)}{\langle c \rangle^2 (k_1 + \alpha k_2)^2 + \sigma^2 (k_1 + \alpha^2 k_2)} \ . \tag{69}$$

Equations 67, 68, and 69 form our analytical solution for the (approximate) fixed points of IBCM neurons in terms of the dot products h_γ taking values y_1 and y_2.

Condition of existence of non-trivial fixed points The fixed points with y_1 and y_2 given by equations 68-69 will only exist in $\mathbb{R}$ if the discriminant $b^2 - 4a_1 a_2$ in α is non-negative. Writing this discriminant explicitly,

$$b^2 - 4a_1 a_2 = 4\sigma^2 \langle c \rangle^2 k_1 k_2 \left(N_\mathrm{B} - \frac{\sigma^2}{\langle c \rangle^2} \right) + 12 m_3 \langle c \rangle k_1 k_2 + m_3^2 \left(\frac{\langle c \rangle^2}{\sigma^4}(k_1 - k_2)^2 + \frac{1}{\langle c \rangle^2} + \frac{2N_\mathrm{B}}{\sigma^2} \right) \ . \tag{70}$$

In general, this expression will be > 0, unless there is very high variance and low average concentration, $\sigma^2 > N_\mathrm{B} \langle c \rangle^2$, and vanishing third moment $m_3 \to 0$. This would be an unnaturalistic setting corresponding to Gaussian, zero-average background fluctuations.

E. Summary of the general fixed point solutions. The fixed point solution for i.i.d. odor concentrations with mean $\langle c \rangle$, variance σ^2, third moment m_3, is summarized here. The fixed points of $\overline{\mathbf{m}}$ are characterized by their dot products with the N_B background odors, $\overline{h}_\gamma = \overline{\mathbf{m}} \cdot \hat{\mathbf{s}}_\gamma$. There are 2^{N_B} fixed points in total: one with all $h_\gamma = 0$, one with all h_γs equal to (subsection D.1)

$$\overline{h}_\gamma = \frac{N_\mathrm{B}^2 \langle c \rangle^3 + 3\sigma^2 N_\mathrm{B} \langle c \rangle + m_3}{N_\mathrm{B}^3 \langle c \rangle^4 + 2\sigma^2 N_\mathrm{B}^2 \langle c \rangle^2 + \sigma^4 N_\mathrm{B}} \ , \tag{62}$$

and $2^{N_\mathrm{B}} - 2$ where k_1 dot products are equal to y_1, and $k_2 = N_\mathrm{B} - k_1$ are equal to y_2, with $\binom{N_\mathrm{B}}{k_1}$ choices for each possible $k_1 \in \{1, \ldots, N_\mathrm{B} - 1\}$. The values y_1 and y_2, where by convention $y_1 > y_2$, are calculated as follows:

$$a_i = \sigma^2 k_i - \langle c \rangle^2 k_i^2 - \frac{m_3 \langle c \rangle}{\sigma^2} k_i \quad (i \in \{1, 2\})$$
$$b = 2 \langle c \rangle^2 k_1 k_2 + \frac{m_3 \langle c \rangle}{\sigma^2}(k_1 + k_2) + \frac{m_3}{\langle c \rangle} \tag{67}$$
$$\alpha = \frac{b \pm \sqrt{b^2 - 4a_1 a_2}}{2a_2} \tag{68}$$

Compute solutions for each sign in α

$$y_1 = \frac{2 \langle c \rangle (k_1 + \alpha k_2) + \frac{m_3}{\sigma^2}(1 + \alpha)}{\langle c \rangle^2 (k_1 + \alpha k_2)^2 + \sigma^2 (k_1 + \alpha^2 k_2)} \tag{69}$$
$$y_2 = \alpha y_1$$

Keep the pair where $y_1 > y_2$

Our linear stability analysis, below, suggests that the *stable* fixed points of an IBCM neuron have one dot product equal to y_1 and all others equal to y_2. This means the neuron becomes selective: it is specifically responding to one background odor.

bioRxiv preprint doi: https://doi.org/10.1101/2025.05.26.656161; this version posted May 30, 2025. The copyright holder for this preprint (which was not certified by peer review) is the author/funder, who has granted bioRxiv a license to display the preprint in perpetuity. It is made available under aCC-BY 4.0 International license.

There are N_B such fixed points. We can therefore call $\mathbf{m}\cdot\hat{\mathbf{s}}_\gamma = y_1 \equiv h_\mathrm{sp}$ (specific) for that odor, and $y_2 \equiv h_\mathrm{ns}$ (non-specific) for all other odors. We observe close quantitative agreement between these fixed point predictions and numerical simulations in the weakly non-Gaussian background, Fig. 3C. For log-normal (Fig. S6D-E) and turbulent backgrounds (Fig. 4A-B), we also observe that IBCM neurons align with individual background odors, but due to stronger correlations between $\mathbf{m}$ and Θ, the exact dot product values do not exactly match equations 67-69.

F. Linear stability analysis of IBCM fixed points. To support our empirical results on IBCM neuron selectivity, we linearize the dynamical equations 51-52 around a fixed point and compute the Jacobian matrix. Then, we evaluate its eigenvalues, at least numerically, for every fixed point, and check which fixed points are stable in a number of examples. Here, we perform this analysis for a single neuron, and assume that weak coupling with other neurons in the network does not fundamentally affect the stability of single-neuron fixed points. We also assume a constant learning rate $\mu_\Theta = \mu$, as would approximately be the case at steady-state in the Law and Cooper variant used for full simulations. Hence, the dynamical equations are

$$\left\langle \frac{\mathrm{d}\mathbf{m}}{\mathrm{d}t} \right\rangle = \mu \left\langle h^2 \mathbf{s}_\mathrm{b} \right\rangle - \mu \left\langle \Theta \right\rangle \left\langle h \mathbf{s}_\mathrm{b} \right\rangle$$
$$\left\langle \frac{\mathrm{d}\Theta}{\mathrm{d}t} \right\rangle = \frac{1}{\tau_\Theta} \left(\left\langle h^2 \right\rangle - \left\langle \Theta \right\rangle \right)$$

Computing the Jacobian entries, recalling that $h = \mathbf{m}\cdot\mathbf{s}$ and thus $\frac{\partial h}{\partial m_i} = s_i$, we find

$$\frac{\partial}{\partial m_i} \left\langle \frac{\mathrm{d}m_j}{\mathrm{d}t} \right\rangle = 2\mu \left\langle h s_i s_j \right\rangle - \mu\Theta \left\langle s_i s_j \right\rangle$$
$$\frac{\partial}{\partial \Theta} \left\langle \frac{\mathrm{d}m_j}{\mathrm{d}t} \right\rangle = -\mu \left\langle h s_j \right\rangle$$
$$\frac{\partial}{\partial m_i} \left\langle \frac{\mathrm{d}\Theta}{\mathrm{d}t} \right\rangle = \frac{2}{\tau_\Theta} \left\langle h s_i \right\rangle$$
$$\frac{\partial}{\partial \Theta} \left\langle \frac{\mathrm{d}\Theta}{\mathrm{d}t} \right\rangle = -\frac{1}{\tau_\Theta} \ .$$

These derivatives form the different blocks of the Jacobian matrix, which is, in vector notation,

$$D\mathbf{f}(\mathbf{m},\Theta) = \left(\begin{array}{c|c} 2\mu \left\langle h \mathbf{s}_\mathrm{b} \mathbf{s}_\mathrm{b}^\intercal \right\rangle & \\ -\mu\Theta \left\langle \mathbf{s}_\mathrm{b} \mathbf{s}_\mathrm{b}^\intercal \right\rangle & \frac{2}{\tau_\Theta} \left\langle h \mathbf{s}_\mathrm{b} \right\rangle \\ \hline -\mu \left\langle h \mathbf{s}_\mathrm{b}^\intercal \right\rangle & -\frac{1}{\tau_\Theta} \end{array} \right) . \tag{71}$$

This expression is general. Now, computing more explicitly the expectation values for i.i.d. concentrations as in previous subsections,

$$\left\langle h \mathbf{s}_\mathrm{b} \right\rangle = h_\mathrm{d} \mathbf{s}_\mathrm{d} + \sigma^2 \sum_\gamma h_\gamma \hat{\mathbf{s}}_\gamma$$
$$\left\langle \mathbf{s}_\mathrm{b} \mathbf{s}_\mathrm{b}^\intercal \right\rangle = \mathbf{s}_\mathrm{d} \mathbf{s}_\mathrm{d}^\intercal + \sigma^2 \sum_\gamma \hat{\mathbf{s}}_\gamma \hat{\mathbf{s}}_\gamma^\intercal$$
$$\left\langle h \mathbf{s}_\mathrm{b} \mathbf{s}_\mathrm{b}^\intercal \right\rangle = h_\mathrm{d} \left\langle \mathbf{s}_\mathrm{b} \mathbf{s}_\mathrm{b}^\intercal \right\rangle + \sigma^2 \sum_\gamma h_\gamma (\mathbf{s}_\mathrm{d} \hat{\mathbf{s}}_\gamma^\intercal + \hat{\mathbf{s}}_\gamma \mathbf{s}_\mathrm{d}^\intercal) + 2m_3 \sum_\gamma h_\gamma \hat{\mathbf{s}}_\gamma \hat{\mathbf{s}}_\gamma^\intercal$$

The last two lines, along with $\Theta = h_\mathrm{d}^2 + \sigma^2 u^2$, means that the main block of the matrix is

$$2\mu \left\langle h \mathbf{s}_\mathrm{b} \mathbf{s}_\mathrm{b}^\intercal \right\rangle - \mu\Theta \left\langle \mathbf{s}_\mathrm{b} \mathbf{s}_\mathrm{b}^\intercal \right\rangle = \mu \left[(2h_\mathrm{d} - h_\mathrm{d}^2 - \sigma^2 u^2)(\mathbf{s}_\mathrm{d} \mathbf{s}_\mathrm{d}^\intercal + \sigma^2 \sum_\gamma \hat{\mathbf{s}}_\gamma \hat{\mathbf{s}}_\gamma^\intercal) + 2m_3 \sum_\gamma h_\gamma \hat{\mathbf{s}}_\gamma \hat{\mathbf{s}}_\gamma^\intercal + 2\sigma^2 \sum_\gamma h_\gamma (\mathbf{s}_\mathrm{d} \hat{\mathbf{s}}_\gamma^\intercal + \hat{\mathbf{s}}_\gamma \mathbf{s}_\mathrm{d}^\intercal) \right]$$

We have defined here $\mathbf{s}_\mathrm{d} = \langle c \rangle \sum_\gamma \hat{\mathbf{s}}_\gamma$. We see that these moments depend on the specific odor components $\hat{\mathbf{s}}_\gamma$, making an analytical calculation of eigenvalues hard in general. However, these expressions can be evaluated easily at the analytical fixed points in several examples to check the stability in these cases; we show the eigenvalues for weakly non-Gaussian, log-normal, and turbulent background statistics in Fig. S3. In all these examples, we find that the only stable fixed points are those where the neuron has one dot product $h_\gamma = h_\mathrm{sp}$ (specific) and the $N_\mathrm{B} - 1$ others are equal to h_ns (non-specific). This property is robust against OSN noise, as shown in Fig. S8. In that case, the IBCM and BioPCA models still perform habituation to the true background subspace and new odor recognition, until the OSN noise becomes comparable in magnitude with odor signals (Fig. S8H).

bioRxiv preprint doi: https://doi.org/10.1101/2025.05.26.656161; this version posted May 30, 2025. The copyright holder for this preprint (which was not certified by peer review) is the author/funder, who has granted bioRxiv a license to display the preprint in perpetuity. It is made available under aCC-BY 4.0 International license.

G. Analytical W weights with IBCM neurons. We can also derive an analytical expression for the average, steady-state values of the inhibitory weights W when the projection weights M converge to the IBCM fixed point derived above. We assume there is at least one neuron per odor, $N_{\mathrm{I}} \geq N_{\mathrm{B}}$. We call γ_j the background odor to which IBCM neuron j is specific; thus, $\mathbf{m}_j \cdot \hat{\mathbf{s}}_\gamma = h_{\mathrm{sp}}$ if $\gamma = \gamma_j$, h_{ns} otherwise. There will be in general some number n_γ of neurons specific to odor γ.

We start by working on the W equation in matrix form,

$$\frac{\mathrm{d}W}{\mathrm{d}t} = \alpha \mathbf{y}(t)\overline{\mathbf{h}}(t)^\intercal - \beta W \ . \tag{72}$$

We define the (constant) matrix Γ, whose columns are the $\hat{\mathbf{s}}_\gamma$, and $\mathbf{c}$ the vector of odor concentrations. Then, $\mathbf{s}_{\mathrm{b}}(t) = \Gamma\mathbf{c}$. We also define the matrix $H = LM\Gamma$, in which row j gives the alignment of IBCM neuron j with each odor, $\overline{h}_{j\gamma}$. Since each neuron is selective for one odor, each row contains h_{sp} once and h_{ns} $N_{\mathrm{B}} - 1$ times. Averaging the W equation over fast c fluctuations, and neglecting correlations between c, W, and H, we have

$$\left\langle \frac{\mathrm{d}W}{\mathrm{d}t} \right\rangle = \alpha(\Gamma - \langle W \rangle \langle H \rangle) \langle \mathbf{c}\mathbf{c}^\intercal \rangle \langle H \rangle^\intercal - \beta \langle W \rangle \ .$$

Average signs on W and H are implied below. We define $N = \langle \mathbf{c}\mathbf{c}^\intercal \rangle$ and evaluate it for i.i.d. odors,

$$N = \langle \mathbf{c}\mathbf{c}^\intercal \rangle = \langle c \rangle^2 O_{N_{\mathrm{B}}} + \sigma^2 \mathbb{I}_{N_{\mathrm{B}}} \ , \tag{73}$$

where $\mathbb{I}$ is the $N_{\mathrm{B}} \times N_{\mathrm{B}}$ identity matrix and $O_{N_{\mathrm{B}}}$ is a $N_{\mathrm{B}} \times N_{\mathrm{B}}$ matrix filled with ones. We now set $\mathrm{d}W/\mathrm{d}t = 0$ and focus on single columns of the equation, with the notation $\mathbf{w}_j$ for column j of W, and $\mathbf{h}_j$ for column j of $H^\intercal$ ($\mathbf{h}_j^\intercal$ is the row j of H). The set of equations to solve for the $\mathbf{w}_j$ is then

$$\frac{\beta}{\alpha}\mathbf{w}_j + WHN\mathbf{h}_j = \Gamma N \mathbf{h}_j \tag{74}$$

We notice here that all IBCM neurons with the same specificity γ_j will have the same $\mathbf{h}_j$, thus all columns $\mathbf{w}_j$ with the same γ_j will be identical, and we can denote them by $\mathbf{w}_{\gamma_j}$. This allows to rewrite sums over columns as sums over components, for instance $\sum_j \mathbf{w}_j = \sum_\gamma n_\gamma \mathbf{w}_\gamma$. We moreover notice that

$$\mathbf{h}_k^\intercal \mathbf{h}_j = \begin{cases} h_{\mathrm{sp}}^2 + (N_{\mathrm{B}} - 1)h_{\mathrm{ns}}^2 = u^2 & \text{if } \gamma_j = \gamma_k \\ 2h_{\mathrm{sp}}h_{\mathrm{ns}} + (N_{\mathrm{B}} - 2)h_{\mathrm{ns}}^2 = h_{\mathrm{ns}}(\overline{h}_{\mathrm{d}}/\langle c \rangle + h_{\mathrm{sp}} - h_{\mathrm{ns}}) & \text{else} \end{cases} \tag{75}$$

and that

$$\mathbf{h}_k^\intercal O_{N_{\mathrm{B}}} \mathbf{h}_j = \mathbf{h}_k^\intercal \mathbf{v}(h_{\mathrm{sp}} + (N_{\mathrm{B}} - 1)h_{\mathrm{ns}}) = (h_{\mathrm{sp}} + (N_{\mathrm{B}} - 1)h_{\mathrm{ns}})^2 = \frac{\overline{h}_{\mathrm{d}}^2}{\langle c \rangle^2} \tag{76}$$

where we defined $\mathbf{v}$, a vector filled with ones, and recognized $\overline{h}_{\mathrm{d}} = \langle c \rangle \sum_\gamma \overline{\mathbf{m}} \cdot \hat{\mathbf{s}}_\gamma = \langle c \rangle^2 (h_{\mathrm{sp}} + (N_{\mathrm{B}} - 1)h_{\mathrm{ns}})$ for all neurons specific to one odor. We also need to compute

$$N\mathbf{h}_j = \sigma^2 \mathbf{h}_j + \langle c \rangle^2 (h_{\mathrm{sp}} + (N_{\mathrm{B}} - 1)h_{\mathrm{ns}})\mathbf{v} = \sigma^2 \mathbf{h}_j + \langle c \rangle \overline{h}_{\mathrm{d}} \mathbf{v}$$

where we used Eq. (73). We use the above results to evaluate the two terms involving N in Eq. (74). With some algebra to combine coefficients efficiently, we find

$$WHN\mathbf{h}_j = \sigma^2 (h_{\mathrm{sp}} - h_{\mathrm{ns}})^2 n_{\gamma_j} \mathbf{w}_{\gamma_j} + A \sum_\gamma n_\gamma \mathbf{w}_\gamma \tag{77}$$

$$\Gamma N \mathbf{h}_j = \sigma^2 (h_{\mathrm{sp}} - h_{\mathrm{ns}}) \hat{\mathbf{s}}_{\gamma_j} + \left(\frac{\sigma^2}{\langle c \rangle} h_{\mathrm{ns}} + \overline{h}_{\mathrm{d}} \right) \mathbf{s}_{\mathrm{d}} \tag{78}$$

where we have used the average background expression, $\mathbf{s}_{\mathrm{d}} = \langle c \rangle \sum_\gamma \hat{\mathbf{s}}_\gamma$, and defined a coefficient independent of j,

$$A = \overline{h}_{\mathrm{d}}^2 + \frac{\sigma^2}{\langle c \rangle} \overline{h}_{\mathrm{d}} h_{\mathrm{ns}} + \sigma^2 h_{\mathrm{ns}} (h_{\mathrm{sp}} - h_{\mathrm{ns}}) \ . \tag{79}$$

We now insert Eq. (77) and Eq. (78) into the equation Eq. (74) for $\mathbf{w}_j$ (or equivalently, $\mathbf{w}_{\gamma_j}$), to find

$$B_{\gamma_j} \mathbf{w}_j + A \sum_\gamma n_\gamma \mathbf{w}_\gamma = \sigma^2 (h_{\mathrm{sp}} - h_{\mathrm{ns}}) \hat{\mathbf{s}}_{\gamma_j} + \left(\frac{\sigma^2}{\langle c \rangle} h_{\mathrm{ns}} + \overline{h}_{\mathrm{d}} \right) \mathbf{s}_{\mathrm{d}} \tag{80}$$

bioRxiv preprint doi: https://doi.org/10.1101/2025.05.26.656161; this version posted May 30, 2025. The copyright holder for this preprint (which was not certified by peer review) is the author/funder, who has granted bioRxiv a license to display the preprint in perpetuity. It is made available under aCC-BY 4.0 International license.

where we have defined another coefficient, different for each j in general, unless all n_{γ_j} are equal,

$$B_{\gamma_j} = \frac{\beta}{\alpha} + n_{\gamma_j}\sigma^2(h_{\mathrm{sp}} - h_{\mathrm{ns}})^2 \; . \tag{81}$$

Now, to solve Eq. (80) for $\mathbf{w}_{\gamma_j}$, we look at the difference between the equations for columns j and k, with $\gamma_k \neq \gamma_j$; this eliminates common terms and allows us to isolate $\mathbf{w}_{\gamma_k}$ in terms of $\mathbf{w}_{\gamma_j}$ and the known vectors $\hat{\mathbf{s}}_\gamma$ only:

$$\begin{aligned} B_{\gamma_j}\mathbf{w}_{\gamma_j} - B_{\gamma_k}\mathbf{w}_{\gamma_k} &= \sigma^2(h_{\mathrm{sp}} - h_{\mathrm{ns}})(\hat{\mathbf{s}}_{\gamma_j} - \hat{\mathbf{s}}_{\gamma_k}) \\ \Rightarrow \mathbf{w}_{\gamma_k} &= \frac{B_{\gamma_j}}{B_{\gamma_k}}\mathbf{w}_{\gamma_j} + \frac{\sigma^2(h_{\mathrm{sp}} - h_{\mathrm{ns}})}{B_{\gamma_k}}(\hat{\mathbf{s}}_{\gamma_k} - \hat{\mathbf{s}}_{\gamma_j}). \end{aligned}$$

Doing this for each $\gamma_k \neq \gamma_j$, and noticing the expression reduces to $\mathbf{w}_{\gamma_j}$ for $\gamma = \gamma_j$, we express $\sum_\gamma n_\gamma \mathbf{w}_\gamma$ in terms of $\mathbf{w}_{\gamma_j}$ only, and insert into eq. Eq. (80) to isolate $\mathbf{w}_{\gamma_j}$. Dividing the result by $B_{\gamma_j}\left(1 + A\sum_\gamma \frac{n_\gamma}{B_\gamma}\right)$, and rearranging with further algebra gives our final expression for columns of the matrix W,

$$\mathbf{w}_{\gamma_j} = \frac{\sigma^2(h_{\mathrm{sp}} - h_{\mathrm{ns}})}{B_{\gamma_j}}\hat{\mathbf{s}}_{\gamma_j} + \frac{\overline{h}_{\mathrm{d}} + \frac{\sigma^2}{\langle c\rangle}h_{\mathrm{ns}}}{B_{\gamma_j}(1+AK)}\mathbf{s}_{\mathrm{d}} - \frac{A\sigma^2(h_{\mathrm{sp}} - h_{\mathrm{ns}})}{B_{\gamma_j}(1+AK)}\sum_\gamma \frac{n_\gamma}{B_\gamma}\hat{\mathbf{s}}_\gamma \; , \tag{82}$$

where we have defined $K = \sum_\rho \frac{n_\rho}{B_\rho}$. These are the analytical expressions that we compare to numerical simulations of the W weights in Figures 3D (weakly non-Gaussian) and S6F (log-normal), reaching close agreement at steady-state. For backgrounds with slower, stronger fluctuations where the correlations between M, W, and $\mathbf{s}(t)$ are not entirely negligible (*e.g.*, turbulent statistics), the agreement would be less accurate. The numbers of neurons selecting each odor, n_{γ_j}, are inferred from the M weights numerical results, then used to evaluate Eq. (82).

Unfortunately, the expression for the instantaneous $\mathbf{y}(t) = \mathbf{s}(t) - WLM\mathbf{s}(t)$ with steady-state M, M weights is quite cumbersome in general, due to the B_γ. However, we find a more compact expression for the average PN response: after simplifying some terms,

$$\langle \mathbf{y}\rangle = \langle c\rangle \sum_\gamma \frac{\hat{\mathbf{s}}_\gamma}{B_\gamma}\frac{1}{1+AK}\left(\frac{\beta}{\alpha} - \sigma^2 h_{\mathrm{ns}}(h_{\mathrm{sp}} - h_{\mathrm{ns}})(n_\gamma N_{\mathrm{B}} - KB_\gamma)\right) \; . \tag{83}$$

The factor $n_\gamma N_{\mathrm{B}} - KB_\gamma$ is zero when all n_γs are equal; hence, the second term represents the bias incurred by having an uneven distribution of IBCM neurons across odor components. The threshold n^* at which $n_\gamma N_{\mathrm{B}} - KB_\gamma = 0$ for some n_γ is $n^* = \frac{\beta/\alpha K}{N_{\mathrm{B}} - K\sigma^2(h_{\mathrm{sp}} - h_{\mathrm{ns}})^2}$; since we can show (using a Lagrange multiplier to enforce $\sum_\rho n_\rho = n_I$) that K is maximized by having a uniform distribution $n_\gamma = n_I/N_{\mathrm{B}}$, the threshold $n^* < n_I/N_{\mathrm{B}}$; hence, all components which have $n_\gamma > n_I/N_{\mathrm{B}}$ surely have a positive factor $(n_\gamma N_{\mathrm{B}} - KB_\gamma)$, and since $h_{\mathrm{ns}} < 0$ in general, they have a negative bias in eq. Eq. (83), i.e. they are suppressed less than other background odor components. Conversely, if some $n_\gamma = 0$ (no neuron specific to that odor), then this odor is still partly subtracted, due to the non-specific response of other neurons, $h_{\mathrm{ns}} < 0$, but at the cost of less efficient inhibition of all other odors, and without overall reduction of fluctuations since the factor $\frac{\beta/\alpha}{B_\gamma} = 1$ if $n_\gamma = 0$.

When all n_γs are equal, the mean PN response to the background, Eq. (83), is minimized for the IBCM network, and it reduces to a simple expression,

$$\langle \mathbf{y}\rangle = \frac{\beta/\alpha}{B + N_{\mathrm{B}}A}\mathbf{s}_{\mathrm{d}} \tag{84}$$

where, recall, A is given by Eq. (79), B by Eq. (81) with all n_γ equal, and $\mathbf{s}_{\mathrm{d}} = \langle c\rangle \sum_\gamma \hat{\mathbf{s}}_\gamma$ is the average background. We will use this simplified expression of the maximal habituation by IBCM neurons determine the Λ factor needed to match the BioPCA and IBCM performances (section 8B).

5. Analytical fixed point solutions of the BioPCA model

We assume that the average background $\langle \mathbf{s}\rangle = \mathbf{s}_{\mathrm{d}} = \langle c\rangle \sum_{\gamma=1}^{N_{\mathrm{B}}} \hat{\mathbf{s}}_\gamma$ is subtracted from $\mathbf{s}$, from the input to the BioPCA inhibitory neurons, and from $\mathbf{y}$; hence, the effective background to consider here is $\tilde{\mathbf{s}} = \sum_{\gamma=1}^{N_{\mathrm{B}}} \tilde{c}_\gamma \hat{\mathbf{s}}_\gamma$, with $\langle \tilde{c}_\gamma\rangle = 0$ and $\langle \tilde{c}_\gamma^2\rangle = \sigma^2$ for all components γ. The LN activity is $\overline{\mathbf{h}}(t) = LM\tilde{\mathbf{s}}(t)$ and the PN response is $\mathbf{y}(t) = \tilde{\mathbf{s}}(t) - W\overline{\mathbf{h}}(t)$. The covariance matrix, from which a PCA with N_{B} components can be computed, is

$$C = \langle \tilde{\mathbf{s}}\tilde{\mathbf{s}}^\intercal\rangle = \sigma^2 \sum_\gamma \hat{\mathbf{s}}_\gamma \hat{\mathbf{s}}_\gamma^\intercal = UDU^\intercal \; ,$$

bioRxiv preprint doi: https://doi.org/10.1101/2025.05.26.656161; this version posted May 30, 2025. The copyright holder for this preprint (which was not certified by peer review) is the author/funder, who has granted bioRxiv a license to display the preprint in perpetuity. It is made available under aCC-BY 4.0 International license.

where U is $N_{\mathrm{s}} \times N_{\mathrm{B}}$, with its columns containing the N_{B} principal component vectors with non-zero eigenvalue, and D is $N_{\mathrm{B}} \times N_{\mathrm{B}}$ and diagonal with the principal values in it, σ_i^2, $i \in \{1, 2, \ldots, N_{\mathrm{B}}\}$. Since the $\hat{\mathbf{s}}_\gamma$ are not orthogonal in general, these eigenvalues are not all equal to the variance σ^2, but should be on the same scale.

Given a background, U and D are known; we can thus express the steady-state solution of the BioPCA model in terms of these PCA matrices. From Lemma 3 in Minden *et al.*, 2018 [47], we expect the BioPCA model with $N_{\mathrm{I}} = N_{\mathrm{B}}$ neurons (one per background component) to have the following stationary solution:

$$L' = D \quad \text{(principal values)} \tag{85}$$

$$LM = \underline{\Lambda} U^{\intercal} \quad \text{(projection on principal components)} \tag{86}$$

where, recall, $L' = L^{-1}$. The input projections give interneuron activities of

$$\overline{\mathbf{h}}(t) = LM\tilde{\mathbf{s}}(t) = \underline{\Lambda} U^{\intercal} \tilde{\mathbf{s}}(t) \ .$$

We insert the BioPCA stationary solution into the W equation 72, where we average over fast $\tilde{\mathbf{s}}$ fluctuations, assuming a separation of time scales between these fluctuations and slow M, L, W learning, as in the IBCM case. Writing the W equation in matrix form, this leads to

$$\begin{aligned}\frac{\mathrm{d}W}{\mathrm{d}t} &= \alpha \left\langle \mathbf{y}(t) \overline{\mathbf{h}}^{\intercal}(t) \right\rangle - \beta W \\ &= \alpha (\mathbb{I} - W \underline{\Lambda} U^{\intercal}) \left\langle \tilde{\mathbf{s}} \tilde{\mathbf{s}}^{\intercal} \right\rangle U \underline{\Lambda}^{\intercal} - \beta W\end{aligned}$$

Setting the W derivative to zero, we can rearrange to isolate W,

$$W(\beta \mathbb{I}/\alpha + \underline{\Lambda} D \underline{\Lambda}^{\intercal}) = UD\underline{\Lambda}^{\intercal} \ .$$

Since D åand $\underline{\Lambda}$ are both diagonal, the matrix in parentheses on the left-hand side is diagonal with entries $\beta/\alpha + \Lambda_{ii}^2 \sigma_i^2$. It is full-rank when $N_{\mathrm{I}} \leq N_{\mathrm{B}}$, so we can invert the equation explicitly. Since $D\underline{\Lambda}^{\intercal}$ is also diagonal, we find

$$W = U \mathrm{diag}\left(\frac{\Lambda_{ii} \sigma_i^2}{\beta/\alpha + \Lambda_{ii}^2 \sigma_i^2} \right) \ . \tag{87}$$

Inserting this W back in the expression for the PN response, $\mathbf{y}(t) = \tilde{\mathbf{s}} - W\overline{\mathbf{h}}$, we find

$$\mathbf{y} = \tilde{\mathbf{s}} - W\underline{\Lambda} U^{\intercal} \tilde{\mathbf{s}} = U \mathrm{diag}\left(\frac{\beta/\alpha}{\beta/\alpha + \Lambda_{ii}^2 \sigma i^2} \right) U^{\intercal} \tilde{\mathbf{s}} \tag{88}$$

where we have used the fact that $UU^{\intercal}$ is a projector on the background subspace to write $\tilde{\mathbf{s}} = UU^{\intercal}\tilde{\mathbf{s}}$, and where $\Lambda_{ii} = \Lambda_{\mathrm{PCA}}(1 - \lambda_r (i+1)/N_{\mathrm{B}})$ for $i = 0, 1, \ldots, N_{\mathrm{I}}$. Hence, we see that the BioPCA network projects the inputs on the principal directions ($U^{\intercal}\tilde{\mathbf{s}}$), reduces the amplitude of each component by a factor $\frac{\beta/\alpha}{\beta/\alpha + \Lambda_{ii}^2 \sigma i^2}$, then reassembles these components (leftmost U). Comparing to equation 84 for the IBCM model, the latter has a better reduction by a factor of approximately $(h_{\mathrm{sp}} - h_{\mathrm{ns}})^2$ in the denominator, hence we need to increase the Λ scale in the BioPCA network to match the performance of these models, as explained in section 8B. We check some of these predictions against numerical simulations in a background with log-normal (Fig. S6B-C) and turbulent (Fig. 4C-D) concentration statistics. We also check that these properties are relatively robust against OSN noise (Fig. S8); the first N_{B} neurons still capture odor directions corresponding to real odors, while additional neurons align with orthogonal OSN noise components (which are part of the full background PCA decomposition).

6. Analytical results for a two-odor simplified background process

To gain further analytical insight into the convergence dynamics of IBCM neurons in particular, we study the simplest non-trivial background, illustrated in Fig. S4A-B. It consists of two odors ($\mathbf{s}_{\mathrm{a}}$, $\mathbf{s}_{\mathrm{b}}$) with fluctuating proportion $\overline{\nu}(t)$ following a Ornstein-Uhlenbeck process (section 2B),

$$\mathbf{s}(t) = \left(\frac{1}{2} + \overline{\nu}(t) \right) \mathbf{s}_{\mathrm{a}} + \left(\frac{1}{2} - \overline{\nu}(t) \right) \mathbf{s}_{\mathrm{b}} \ . \tag{89}$$

We start by calculating the average fixed points of the IBCM neurons synaptic weights, $\overline{\mathbf{m}}_i$. The fixed point equations 54 are identical for all neurons, so we focus on one neuron and omit index i. As in section 4, we assume that time scales are well separated, replace $\Theta = \langle h^2 \rangle$, and neglect correlations between $\overline{\nu}$, Θ, and $\mathbf{m}$. We work with reduced weights and activities $\overline{h}_i$, averaged over fast fluctuations, so overlines and $\langle \, \rangle$ are implied for the rest of the section. Individual neurons' weights $\mathbf{m}$ can be

bioRxiv preprint doi: https://doi.org/10.1101/2025.05.26.656161; this version posted May 30, 2025. The copyright holder for this preprint (which was not certified by peer review) is the author/funder, who has granted bioRxiv a license to display the preprint in perpetuity. It is made available under aCC-BY 4.0 International license.

recovered from equation Eq. (55). Moreover, for this simple background, the learning rate can be chosen constant, $\mu_{\langle\overline{\Theta}_i\rangle} = \mu$. Hence, the fixed point equation to solve here is

$$0 = \mu \left\langle h^2 \mathbf{s}(t) \right\rangle - \langle \Theta \rangle \left\langle h \mathbf{s}(t) \right\rangle$$

Now, we rewrite

$$\mathbf{s}(t) = \mathbf{s}_\mathrm{d} + \overline{\nu}(t)\mathbf{s}_\mathrm{s} \ ,$$

where we have defined $\mathbf{s}_\mathrm{d} = \frac{1}{2}(\mathbf{s}_\mathrm{a} + \mathbf{s}_\mathrm{b})$ (deterministic part) and $\mathbf{s}_\mathrm{s} = \mathbf{s}_\mathrm{a} - \mathbf{s}_\mathrm{b}$ (stochastic part). We examine the dot products of synaptic weights with these components, $h_\mathrm{d} = \mathbf{m} \cdot \mathbf{s}_\mathrm{d}$ and $h_\mathrm{s} = \mathbf{m} \cdot \mathbf{s}_\mathrm{s}$, such that $h(t) = h_\mathrm{d} + \overline{\nu}(t) h_\mathrm{s}$. We can solve for the two dot products h_d and h_s because they specify the fixed points completely for $N_\mathrm{B} = 2$ background components. In term of these quantities, the fixed point equation becomes

$$0 = \left\langle (h_\mathrm{d} + \overline{\nu} h_\mathrm{s})^2 (\mathbf{s}_\mathrm{d} + \overline{\nu}\mathbf{s}_\mathrm{s}) \right\rangle - \left\langle (h_\mathrm{d} + \overline{\nu} h_\mathrm{s})^2 \right\rangle \left\langle (h_\mathrm{d} + \overline{\nu} h_\mathrm{s}) (\mathbf{s}_\mathrm{d} + \overline{\nu}\mathbf{s}_\mathrm{s}) \right\rangle$$
$$0 = (h_\mathrm{d}^2 + \sigma^2 h_\mathrm{s}^2 - h_\mathrm{d}^3 - h_\mathrm{d} h_\mathrm{s}^2 \sigma^2) \mathbf{s}_\mathrm{d} + (2 h_\mathrm{s} h_\mathrm{d} - h_\mathrm{d}^2 h_\mathrm{s} - \sigma^2 h_\mathrm{s}^3) \sigma^2 \mathbf{s}_\mathrm{s}$$

Since $\mathbf{s}_\mathrm{d}$ and $\mathbf{s}_\mathrm{s}$ are linearly independent, both coefficients must be zero, leading to a system of two equations for h_d and h_s,

$$0 = h_\mathrm{d}^3 - h_\mathrm{d}^2 - \sigma^2 h_\mathrm{s}^2 + h_\mathrm{d} h_\mathrm{s}^2 \sigma^2$$
$$0 = \sigma^2 h_\mathrm{s} (h_\mathrm{d}^2 + \sigma^2 h_\mathrm{s}^2 - 2 h_\mathrm{d}) \ .$$

There is a trivial solution $h_\mathrm{s} = h_\mathrm{d} = 0$, which is unstable. The other solutions are, by inspection,

$$h_\mathrm{d} = \mathbf{m} \cdot \mathbf{s}_\mathrm{d} = 1 \text{ and } h_\mathrm{s} = \mathbf{m} \cdot \mathbf{s}_\mathrm{s} = \pm\frac{1}{\sigma} \ ,$$

or, in terms of the dot products with $\mathbf{s}_\mathrm{a}$ and $\mathbf{s}_\mathrm{b}$,

$$\mathbf{m}_\pm \cdot \mathbf{s}_\mathrm{a} = 1 \pm \frac{1}{2\sigma} \text{ and } 1 \mp \frac{1}{2\sigma} \ .$$

Hence, we have two different stable fixed points, which we call $\mathbf{m}_+$ and $\mathbf{m}_-$ to indicate which sign the dot product with $\mathbf{s}_\mathrm{s}$ takes. Figure S4C shows the convergence of a two-neuron network to these fixed points. To interpret these expressions, consider the response at the fixed point to some input sample $\mathbf{s}(t)$:

$$h_\pm(t) = \mathbf{m}_\pm \cdot (\mathbf{s}_\mathrm{s} + \overline{\nu}(t)\mathbf{s}_\mathrm{d}) = 1 \pm \frac{\overline{\nu}(t)}{\sigma} \tag{90}$$

We notice that $h_\pm = 0$ when $\overline{\nu} = \mp\sigma$, that is, the IBCM neuron is non-responsive to an odor component one standard deviation away on one side of the average background, while it responds strongly to odors on the other side of the average. Hence, in this simplified background, the specificity property of IBCM neurons translates into selecting inputs one standard deviation away from the average.

A. Analytical results: PN inhibition. From the steady-state solution for $\mathbf{m}$, we can also compute the steady-state inhibitory weights $\mathbf{w}$. We assume there are $N_\mathrm{I} = 2$ neurons, one at each fixed point $\pm$.

Averaging the W equation 72 over background fluctuations, writing out $\mathbf{y} = \mathbf{s} - WLM\mathbf{s}$, and focusing on the column for one neuron j, we have

$$\frac{\mathrm{d}\left\langle \mathbf{w}_j \right\rangle}{\mathrm{d}t} = \alpha \left\langle \overline{h}_j (\mathbf{s} - WLM\mathbf{s}) \right\rangle - \beta \left\langle \mathbf{w}_j \right\rangle \ \ \forall j \ . \tag{91}$$

To solve for $\mathbf{w}_j$, we set the derivative to zero, and we assume there are $N_\mathrm{I} = 2$ IBCM neurons, one at each fixed point $\pm$. We still assume a separation of time scales, and assume the $\overline{\mathbf{m}}$ are equal to their average fixed point values, so at any time t, the IBCM neuron activity $\overline{h}(t)$ is given by Eq. (90). We assume the two neurons converge to fixed points $+$ and $-$, respectively. We thus establish equations to solve for the $\mathbf{w}_j$ weights fixed point values, $\mathbf{w}_+$ and $\mathbf{w}_-$. We have

$$\frac{\mathrm{d}\mathbf{w}_\pm}{\mathrm{d}t} = 0 = \alpha \left\langle \left(1 \pm \frac{\overline{\nu}(t)}{\sigma}\right) \left[\mathbf{s}_\mathrm{d} + \overline{\nu}(t)\mathbf{s}_\mathrm{s} - \mathbf{w}_+ \left(1 + \frac{\overline{\nu}(t)}{\sigma}\right) - \mathbf{w}_- \left(1 - \frac{\overline{\nu}(t)}{\sigma}\right) \right] \right\rangle - \beta \mathbf{w}_\pm \ .$$

Solving for $\mathbf{w}_+$ and $\mathbf{w}_-$, we find answers summarized as

$$\mathbf{w}_\pm = \frac{\alpha}{2\alpha + \beta} (\mathbf{s}_\mathrm{d} \pm \sigma \mathbf{s}_\mathrm{s}) \tag{92}$$

bioRxiv preprint doi: https://doi.org/10.1101/2025.05.26.656161; this version posted May 30, 2025. The copyright holder for this preprint (which was not certified by peer review) is the author/funder, who has granted bioRxiv a license to display the preprint in perpetuity. It is made available under aCC-BY 4.0 International license.

Hence, each IBCM neuron inhibits the off-average component for which it is selective, $\mathbf{s}(\overline{\nu} = \pm\sigma)$. Combining the two IBCM neurons, the instantaneous PN activity is reduced to

$$\begin{aligned}\mathbf{s}(t) &= \mathbf{s}_{\mathrm{d}} + \overline{\nu}(t)\mathbf{s}_{\mathrm{s}} - \overline{h}_{+}\mathbf{w}_{+} - \overline{h}_{-}\mathbf{w}_{-} \\ &= \mathbf{s}_{\mathrm{d}} - \frac{\alpha}{2\alpha+\beta}\left(1 + \frac{\overline{\nu}}{\sigma}\right)(\mathbf{s}_{\mathrm{d}} + \sigma\mathbf{s}_{\mathrm{s}}) - \frac{\alpha}{2\alpha+\beta}\left(1 - \frac{\overline{\nu}}{\sigma}\right)(\mathbf{s}_{\mathrm{d}} - \sigma\mathbf{s}_{\mathrm{s}}) \\ &= \frac{\beta/\alpha}{2+\beta/\alpha}\mathbf{s}(t)\ . \end{aligned} \tag{93}$$

Figure S4F-G show close agreement at steady-state between numerical simulations and equations 93 and 92. Hence, by learning $N_{\mathrm{B}} = 2$ linearly independent components $\mathbf{w}_{\pm}$ that are one standard deviation away from the average background, the network is able to suppress any $\mathbf{s}(t)$ from that background, in real time, to a fraction $\frac{\beta}{2\alpha+\beta}$ of its original amplitude. Therefore, not only the average, but also the variance of the background is reduced: background fluctuations are actively suppressed by the IBCM-inhibitory neuron pairs. However, new odors would not be suppressed in the same way, because they have a component orthogonal to the vector space of learnt background.

B. Analytical results: convergence time. The convergence time of the $\mathbf{m}$ weights of an IBCM neuron can be estimated analytically in the two-odor simplified background; this analysis reveals the main parameters influencing how long it takes to habituate to a fluctuating background. Numerically, we observe that with the α, β rates chosen, W weights converge at a similar pace.

We again make a quasi-static approximation on the threshold Θ, assuming it averages over the fast background fluctuations but also converges fast enough to track the slow variations of $\mathbf{m}$,

$$\Theta = \langle(\mathbf{m}\cdot\mathbf{s}_{\mathrm{d}} + \overline{\nu}\mathbf{m}\cdot\mathbf{s}_{\mathrm{s}})^2\rangle = h_{\mathrm{d}}^2 + \sigma^2 h_{\mathrm{s}}^2$$

where we made use of $\langle\overline{\nu}\rangle = 0$ and $h(t) = h_{\mathrm{d}} + \overline{\nu}(t)h_{\mathrm{s}}$. Then, we derive dynamical equations for the slow variables h_{d} and h_{d}, by taking the dot product of $\frac{\mathrm{d}\mathbf{m}}{\mathrm{d}t}$ with $\mathbf{s}_{\mathrm{s}}$ and $\mathbf{s}_{\mathrm{d}}$, averaging over fast time scales of $\overline{\nu}(t)$, and using the quasi-static Θ above. To simplify calculations, we assume that the two odor vectors, $\mathbf{s}_{\mathrm{a}}$ and $\mathbf{s}_{\mathrm{b}}$, have the same norm (like the $\hat{\mathbf{s}}_{\gamma}$ in the general case are unit normed); in this case, the vectors $\mathbf{s}_{\mathrm{d}}$ and $\mathbf{s}_{\mathrm{s}}$ are orthogonal. Making use of these properties, we calculate for instance, for h_{d},

$$\begin{aligned}\frac{\mathrm{d}h_{\mathrm{d}}}{\mathrm{d}t} &= \left\langle \frac{\mathrm{d}\mathbf{m}}{\mathrm{d}t}\cdot\mathbf{s}_{\mathrm{d}}\right\rangle = \mu\left\langle(h_{\mathrm{d}} + \overline{\nu}h_{\mathrm{s}})(h_{\mathrm{d}} + \overline{\nu}h_{\mathrm{s}} - \Theta)\right\rangle \mathbf{s}_{\mathrm{d}}^2 \\ &= \mu\left(h_{\mathrm{d}}^2 + \sigma^2 h_{\mathrm{s}}^2 - h_{\mathrm{d}}\left(h_{\mathrm{d}}^2 + \sigma^2 h_{\mathrm{s}}^2\right)\right)\mathbf{s}_{\mathrm{d}}^2 \\ &= \mu\mathbf{s}_{\mathrm{d}}^2(1 - h_{\mathrm{d}})\left(h_{\mathrm{d}}^2 + \sigma^2 h_{\mathrm{s}}^2\right)\ . \end{aligned} \tag{94}$$

By a similar calculation, we find for h_{s}

$$\frac{\mathrm{d}h_{\mathrm{s}}}{\mathrm{d}t} = \mu\sigma^2\mathbf{s}_{\mathrm{s}}^2 h_{\mathrm{s}}\left(2h_{\mathrm{d}} - \left(h_{\mathrm{d}}^2 + \sigma^2 h_{\mathrm{s}}^2\right)\right)\ . \tag{95}$$

From equations Eq. (94) and Eq. (95), we can conclude there will be two phases to the dynamics if the initial values of $h_{\mathrm{s}}(0) = \epsilon_{\mathrm{s}}$ and $h_{\mathrm{d}}(0) = \epsilon_{\mathrm{d}}$ are small, and σ^2 is small also. The only positive term in $\frac{\mathrm{d}h_{\mathrm{s}}}{\mathrm{d}t}$ contains h_{d}; hence, as long as h_{d} is small, h_{s} will remain close to zero. The first phase therefore consists in the growth of h_{d} to its steady-state value of 1, while h_{s} remains approximately equal to its initial value, ϵ_{s}. We call t_{d} its duration. After h_{d} has converged, the second phase consists in the growth of h_{s}. We call t_{s} the duration of that phase. Hence, h_{s} reaches steady-state after a total time of $t_{\mathrm{d}} + t_{\mathrm{s}}$.

We compute t_{d} (first phase duration) by integrating equation Eq. (94) from 0 to some fraction ξ (close to unity; we use $\xi = 0.9$ in practice) of the steady-state $h_{\mathrm{d}} = 1$, with the assumption that h_{s}^2 is approximately constant and sub-dominant in that phase, i.e. $h_{\mathrm{s}}^2 \approx \epsilon_{\mathrm{s}}^2 \approx 0$. We find

$$\begin{aligned}\int_{\epsilon_{\mathrm{d}}}^{\xi}\frac{\mathrm{d}h_{\mathrm{d}}}{h_{\mathrm{d}}^2(1 - h_{\mathrm{d}})} &= \int_0^{t_{\mathrm{d}}}\mu s_{\mathrm{d}}^2\mathrm{d}t \\ \Rightarrow t_{\mathrm{d}} &= \frac{1}{\mu s_{\mathrm{d}}^2}\left[\frac{1}{\epsilon_{\mathrm{d}}} - \frac{1}{\xi} + \ln\left(\frac{\xi(1 - \epsilon_{\mathrm{d}})}{\epsilon_{\mathrm{d}}(1 - \xi)}\right)\right] \end{aligned} \tag{96}$$

where $s_{\mathrm{d}}^2 = \|\mathbf{s}_{\mathrm{d}}\|^2$.

Then, once $h_{\mathrm{d}} \approx 1$, the second phase starts. We neglect sub-dominant terms and we integrate from t_{d} (time at which $h_{\mathrm{d}} \approx 1$ but $h_{\mathrm{s}} \approx \epsilon_{\mathrm{s}}$ still) to $t_{\mathrm{d}} + t_{\mathrm{s}}$. We integrate h_{s} from ϵ_{s} to $\pm\xi/\sigma$: depending on the sign of the initial value ϵ_{s}, the system goes to

bioRxiv preprint doi: https://doi.org/10.1101/2025.05.26.656161; this version posted May 30, 2025. The copyright holder for this preprint (which was not certified by peer review) is the author/funder, who has granted bioRxiv a license to display the preprint in perpetuity. It is made available under aCC-BY 4.0 International license.

either fixed point $\pm 1/\sigma$ (same sign as the initial value). Hence,

$$\frac{\mathrm{d}h_{\mathrm{s}}}{\mathrm{d}t} \approx \mu \mathbf{s}_{\mathrm{s}}^2 \sigma^2 h_{\mathrm{s}}$$
$$\Rightarrow \int_{\epsilon_{\mathrm{s}}}^{\pm\xi/\sigma} \frac{\mathrm{d}h_{\mathrm{s}}}{h_{\mathrm{s}}} = \mu \mathbf{s}_{\mathrm{s}}^2 \sigma^2 h_{\mathrm{s}} \int_{t_{\mathrm{d}}}^{t_{\mathrm{s}}+t_{\mathrm{d}}} \mathrm{d}t$$
$$\Rightarrow t_{\mathrm{s}} = \frac{1}{\mu \mathbf{s}_{\mathrm{s}}^2 \sigma^2} \ln \left| \frac{\xi}{\sigma \epsilon_{\mathrm{s}}} \right| \; . \tag{97}$$

Fig. S4D and E show that the approximations Eq. (96) and Eq. (97) hold well in a range of initial values ϵ_{s} between 0.005 and 0.05, and ϵ_{d} between 0.01 and 0.1. Importantly, these analytical expressions show that convergence time is faster if initial conditions are larger (note inverse ϵ terms) and the noise σ^2 is larger. It means that initial conditions must be chosen to avoid very small dot products with inputs initially. Then, larger background fluctuations trigger faster convergence too, at least in this simple background model. This is a surprising property of the IBCM model: fluctuations drive the dynamics.

C. BioPCA neuron on the two-odor simplified background. We also characterized the behavior of the BioPCA model in this simplified background. Since the background effectively has one principal direction, $\mathbf{s}_{\mathrm{s}}$ (Fig. S7A), a single BioPCA neuron is needed to capture it. Then, the matrix M is only a row vector $\mathbf{m}^{\intercal}$ and the matrix L is only a scalar ℓ, so $L' = \ell' = 1/\ell$. Likewise, W is a column vector $\mathbf{w}$ and the matrix $\underline{\Lambda}$ is just the scalar parameter Λ. Since we assume BioPCA receives the input with the average subtracted, the background we consider is $\tilde{\mathbf{s}} = \overline{\nu}(t)\mathbf{s}_{\mathrm{s}}$. We illustrate how to solve the BioPCA steady-state equations in this simple case. With $\overline{h} = \overline{\nu}(t)\mathbf{m}^{\intercal}\mathbf{s}_{\mathrm{s}}/\ell'$, the dynamical equations simplify to

$$\frac{1}{\mu_M}\frac{\mathrm{d}\mathbf{m}^{\intercal}}{\mathrm{d}t} = (\overline{\nu}(t)\mathbf{m}^{\intercal}\mathbf{s}_{\mathrm{s}}/\ell')\overline{\nu}(t)\mathbf{s}_{\mathrm{s}}^{\intercal} - \mathbf{m}^{\intercal}$$
$$\frac{1}{\mu_L}\frac{\mathrm{d}\ell'}{\mathrm{d}t} = \overline{\nu}(t)\mathbf{m}^{\intercal}\mathbf{s}_{\mathrm{s}}/\ell')^2 - \Lambda^2 \ell'$$

Averaging over $\overline{\nu}(t)$ and setting the derivatives equal to zero, we have two fixed point equations,

$$0 = \sigma^2 h_{\mathrm{s}}/\ell' \mathbf{s}_{\mathrm{s}} - \mathbf{m}$$
$$0 = \sigma^2 h_{\mathrm{s}}^2/\ell'^2 - \Lambda^2 \ell'$$

where we have defined $h_{\mathrm{s}} = \mathbf{m}^{\intercal}\mathbf{s}_{\mathrm{s}}$. The first equation shows that $\mathbf{m}$ is parallel to the first principal component: $\mathbf{m} = \|\mathbf{m}\|\mathbf{s}_{\mathrm{s}}/\|\mathbf{s}_{\mathrm{s}}\|$. To find its magnitude, we take the dot product of that equation with $\mathbf{s}_{\mathrm{s}}$, which allows to factor out h_{s} and solve for ℓ'. We find that ℓ' does converge to the first principal eigenvalue, which is $\sigma^2\|\mathbf{s}_{\mathrm{s}}\|^2$ in this simplified background,

$$L_{11} = 1/L'_{11} = \frac{1}{\sigma^2 \|\mathbf{s}_{\mathrm{s}}\|^2} \; . \tag{98}$$

From the second equation, we then find $h_{\mathrm{s}} = \Lambda\sigma^2\|\mathbf{s}_{\mathrm{s}}\|^3$, which means that $\mathbf{m}$ has a norm $\|\mathbf{m}\| = \Lambda\sigma^2\|\mathbf{s}_{\mathrm{s}}\|^2$. Hence, we have $\mathbf{m}$ parallel to the first principal component,

$$\mathbf{m} = \sigma^2 \|\mathbf{s}_{\mathrm{s}}\|\mathbf{s}_{\mathrm{s}} \; . \tag{99}$$

Also, the instantaneous response of this neuron to $\tilde{\mathbf{s}}$ is $\overline{h}(t) = \ell\mathbf{m}\cdot\tilde{\mathbf{s}}(t) = \Lambda\|\mathbf{s}_{\mathrm{s}}\|\overline{\nu}(t)$. Inserting these expressions in the $\mathbf{w}$ equation for this single neuron,

$$\frac{\mathrm{d}\mathbf{w}}{\mathrm{d}t} = \alpha(\Lambda\|\mathbf{s}_{\mathrm{s}}\|\overline{\nu}(t))(\overline{\nu}(t)\mathbf{s}_{\mathrm{s}} - \mathbf{w}(\Lambda\|\mathbf{s}_{\mathrm{s}}\|\overline{\nu}(t))) - \beta\mathbf{w} \; .$$

Averaging over $\overline{\nu}(t)$ and solving for $\mathbf{w}$, we find that it is also a vector parallel to $\mathbf{s}_{\mathrm{s}}$,

$$\mathbf{w} = \frac{\sigma^2\Lambda\|\mathbf{s}_{\mathrm{s}}\|}{\sigma^2\|\mathbf{s}_{\mathrm{s}}\|^2\Lambda^2 + \beta/\alpha}\mathbf{s}_{\mathrm{s}} \; . \tag{100}$$

Lastly, computing the PN instantaneous activity once the BioPCA neuron has reached its learning fixed point, we find

$$\mathbf{y}(t) = \tilde{\mathbf{s}}(t) - WLM\tilde{\mathbf{s}}(t) = \frac{\beta/\alpha}{\beta/\alpha + \sigma^2\Lambda^2\|\mathbf{s}_{\mathrm{s}}\|^2}\overline{\nu}(t)\mathbf{s}_{\mathrm{s}} \; . \tag{101}$$

Fig. S7 shows that these analytical predictions for $\mathbf{m}$, L, $\mathbf{w}$, and $\mathbf{y}(t)$ match numerical simulations very well. Hence, by learning the direction of fluctuations along the first principal component, the BioPCA neuron reduces the mean and standard deviation of fluctuations by a factor $\frac{\beta/\alpha}{\beta/\alpha+\sigma^2\Lambda^2\|\mathbf{s}_{\mathrm{s}}\|^2}$, to be compared with the reduction achieved by the IBCM network, in equation 93.

bioRxiv preprint doi: https://doi.org/10.1101/2025.05.26.656161; this version posted May 30, 2025. The copyright holder for this preprint (which was not certified by peer review) is the author/funder, who has granted bioRxiv a license to display the preprint in perpetuity. It is made available under aCC-BY 4.0 International license.

7. Testing different L-norms for the W Hebbian learning rule

The Hebbian learning rule for W used in the main text (derived in *Methods*) causes our proposed models to subtract the entire component of the input lying in the background subspace. It gives them a sub-optimal performance, limited to the similarity between the new odor and its orthogonal component (Figures 5, S10). To start exploring alternative W rules that could better exploit the odor-specific projections learnt by IBCM neurons, we considered the effect of using different L^p-norms in the cost function from which the W dynamics are derived. We write a cost function for W weights based on the L^p-norm of PN activity and the entry-wise L^q-norm of W (generalization of the Frobenius norm, which corresponds to $q = 2$), defined as

$$\|\mathbf{y}\|_p = \left(\sum_{i=1}^{N_\mathrm{S}} |y_i|^p \right)^{1/p}$$

$$\|W\|_q = \left(\sum_{i=1}^{N_\mathrm{S}} \sum_{j=1}^{N_\mathrm{I}} \|W_{ij}\|^q \right)^{1/q} .$$

In the cost function, we square terms to preserve direct comparisons with the default L^2-norm cost function:

$$\mathcal{L}_W = \frac{1}{2}\|\mathbf{y}\|_p^2 + \frac{\beta}{2\alpha}\|W\|_q^2 .$$

Taking gradient descent dynamics on this loss function gives a generalized Hebbian learning rule,

$$\frac{\mathrm{d}W_{ij}}{\mathrm{d}t} = \alpha\|\mathbf{y}\|_p^{2-p}|y_i|^{p-1}\mathrm{sgn}(y_i)\overline{h}_j - \beta\|W\|_q^{2-q}|W_{ij}|^{q-1}\mathrm{sgn}(W_{ij}) . \quad \textbf{(102)}$$

Notice that it reduces to the main text rule when $p = q = 2$. We performed numerical experiments of habituation and new odor recognition, analogous to Fig. 2, for various p, q choices in this generalized Hebbian rule. For each (p, q) choice, we optimized the performance with a grid search over a few α and β learning rate values, centered on relevant windows (*e.g.*, a smaller p requires a smaller α to prevent numerical instabilities). Unfortunately, different p, q choices did not fundamentally alter the model performance for habituation (Fig. S9A-C) or new odor recognition (Fig. S9D-F); in fact, the default L^2-norm provided the best results. Hence, more strongly nonlinear versions of manifold learning, such as online manifold tiling [57], or learning rules with positive feedbacks to learn the optimal matrix P of section 1, would be needed to further improve new odor recognition performance.

8. Projection weights scale factor, Λ

As explained in *Methods*, we defined a parameter Λ controlling the scale of M weights and compensating for the regularization on W. Here, we explain how to introduce Λ in the IBCM model, and how to set this parameter to make BioPCA and IBCM perform equivalently.

A. Introducing a scale factor Λ in the IBCM model. We start with the equations for a single neuron. We seek to introduce Λ where appropriate in the equations to maintain the exact same dynamics, only with the numerical values of $\mathbf{m}$ weights (including their initial values) scaled by some factor Λ. By definition, as we scale $\mathbf{m} \sim \Lambda$, then $h = \mathbf{m} \cdot \mathbf{s} \sim \Lambda$ as well. The IBCM equation contains terms of the form $h - \Theta$, with $\Theta \sim h^2$; to keep these terms matched, we need $\Theta \sim \Lambda$, which we achieve by letting $\Theta \to h^2/\Lambda$. Hence, we start by modifying the threshold equation to

$$\frac{\mathrm{d}\Theta}{\mathrm{d}t} = \frac{1}{\tau_\Theta}(h^2/\Lambda - \Theta) . \quad \textbf{(103)}$$

We do not need to rescale the learning rate here, because both sides have a homogeneous scaling $\sim \Lambda$. However, in the $\mathbf{m}$ equation, we need to rescale the learning rate to preserve the dynamics, because the right-hand side has terms $\sim h^2$. To keep both sides scaling as $\frac{\mathrm{d}\mathbf{m}}{\mathrm{d}t} \sim \Lambda$, we modify the $\mathbf{m}$ equation to

$$\frac{\mathrm{d}\mathbf{m}}{\mathrm{d}t} = \frac{\mu_\Theta}{\Lambda}h(h-\Theta)\mathbf{s}(t) - \varepsilon\frac{\mu_\Theta}{\Lambda}\mathbf{m}, . \quad \textbf{(104)}$$

Moreover, since the scale of $\Theta \sim \Lambda$, we need to rescale it in the Θ-dependent learning rate (from our variant of the Law and Cooper version of IBCM),

$$\mu_\Theta = \frac{\mu_0}{\Theta/\Lambda + k_\Theta} . \quad \textbf{(105)}$$

bioRxiv preprint doi: https://doi.org/10.1101/2025.05.26.656161; this version posted May 30, 2025. The copyright holder for this preprint (which was not certified by peer review) is the author/funder, who has granted bioRxiv a license to display the preprint in perpetuity. It is made available under aCC-BY 4.0 International license.

The generalization to a network of IBCM neurons is straightforward, since Λ is a unique scale parameter for all neurons. Each $\overline{\Theta}_i$ equation has the rescaled term $\overline{h}_i^2/\Lambda$ as in 103. All terms in neuron i's $\mathbf{m}_i$ equation, including those from coupling with other neurons j, have their learning rates $\mu_{\overline{\Theta}_j}$ divided by Λ, as in 104. Also, $\overline{\Theta}_i$ is divided by Λ in the denominator of each learning rate $\mu_{\overline{\Theta}_j}$ as in eq. 105.

These are the IBCM equations we use for general Λ values. In Fig. S11, we characterize the performance of the network for habituation and new odor recognition as as function of the scale Λ, and we observe that $\Lambda_{\mathrm{IBCM}} = 1$ is large enough to maximize the performance.

B. Scaling the BioPCA model for performance equivalent to IBCM. As explained in *Methods*, the scale Λ is already built into the BioPCA model, in the matrix $\underline{\Lambda}$ intervening in the L equation. Λ_{PCA} is set to 1 by default [47], but this leads to a smaller M weights magnitude than by default in the IBCM model. For this reason, Fig. S11 shows that the BioPCA model requires a $\Lambda_{\mathrm{PCA}} > 1$ to achieve the same performance.

For other simulations where we compare the two models, we use our analytical results on the IBCM and BioPCA models to estimate beforehand what Λ_{PCA} value should yield a comparable performance from both models. As derived in Eq. (84), the PN response is reduced, in an IBCM network, by a factor

$$f_{\mathrm{IBCM}} = \frac{\beta/\alpha}{\beta/\alpha + \sigma^2 (h_{\mathrm{sp}} - h_{\mathrm{ns}})^2 \Lambda_{\mathrm{IBCM}}^2 + N_{\mathrm{B}} \left(\overline{h}_{\mathrm{d}}^2 + \sigma^2 \overline{h}_{\mathrm{d}} h_{\mathrm{ns}} / \langle c \rangle + \sigma^2 h_{\mathrm{ns}} (h_{\mathrm{sp}} - h_{\mathrm{ns}}) \right) \Lambda_{\mathrm{IBCM}}^2}$$

where we have explicited how Λ_{IBCM} controls this factor by multiplying the default-scale LN activities, $h_{\mathrm{sp}}, h_{\mathrm{ns}}, \overline{h}_{\mathrm{d}}$ – recall that these are the specific, non-specific, and average alignments of the IBCM fixed points, derived in section 4.

In comparison, Eq. (88) shows that the PN activity in a BioPCA network is reduced along each principal direction by a factor of approximately

$$f_{\mathrm{PCA}} = \frac{\beta/\alpha}{\beta/\alpha + \sigma^2 \Lambda_{\mathrm{PCA}}^2} \ .$$

We set Λ_{PCA} to make these two factors equal, which occurs at

$$f_{\mathrm{IBCM}} = f_{\mathrm{PCA}} \;\; \Rightarrow \;\; \Lambda_{\mathrm{PCA}} = \frac{\beta}{\alpha \sigma^2} \frac{1 - f_{\mathrm{IBCM}}}{f_{\mathrm{IBCM}}} \ . \qquad \textbf{(106)}$$

So, to set up parameters for a numerical simulation, we compute the statistics of the chosen background ($\langle c \rangle$, σ^2, m_3), the analytical predictions for the IBCM fixed points (h_{sp}, h_{ns}, $\overline{h}_{\mathrm{d}}$), and we set Λ_{PCA} to these parameter values inserted in Eq. (106).

bioRxiv preprint doi: https://doi.org/10.1101/2025.05.26.656161; this version posted May 30, 2025. The copyright holder for this preprint (which was not certified by peer review) is the author/funder, who has granted bioRxiv a license to display the preprint in perpetuity. It is made available under aCC-BY 4.0 International license.

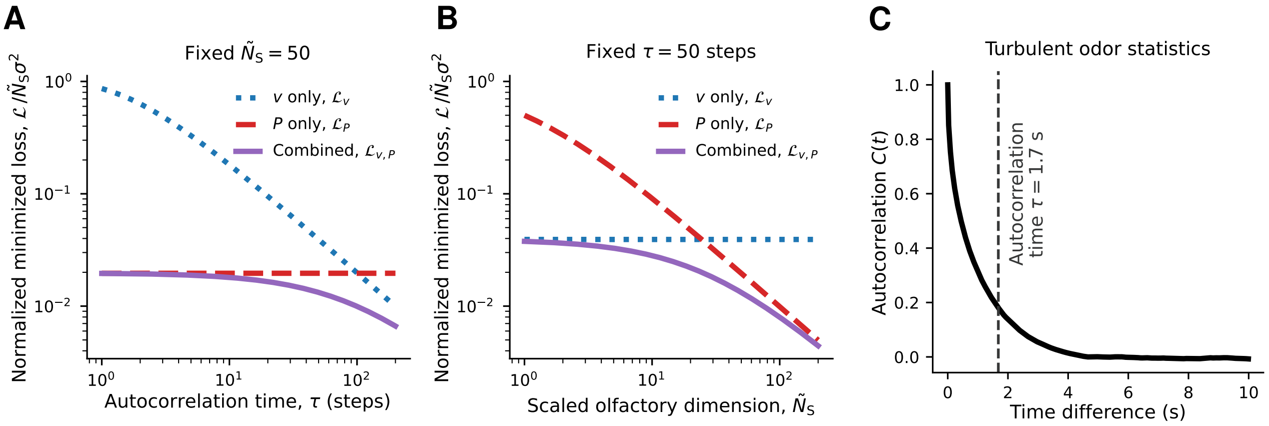

Fig. S1. Comparing manifold learning and predictive filtering. Supplement to Fig. 1E. (**A**) Minimized loss function for new odor recognition in a simple background (Ornstein-Uhlenbeck), for the combined strategies (purple) and either single strategy (blue, red), as a function of the autocorrelation time τ, for fixed olfactory space dimensionality. The combined strategy is always better, but manifold learning explains essentially all the loss reduction at low autocorrelation times ($\mathcal{L}_{P,v} \approx \mathcal{L}_P$). (**B**) Same, as a function of the scaled olfactory space dimension, $\tilde{N}_S = N_S\sigma^2/\sigma^2_{\text{new}}$, for fixed autocorrelation time $\tau = 50$ steps. Most of the loss reduction comes from one strategy or the other on either side of the crossover region; manifold learning dominates in high dimensions. (**C**) Autocorrelation function of the concentration fluctuations in the turbulent background of Fig. 1B-C, showing an autocorrelation time of ~ 1.7 s.
